# Saltations of *cis*-regulatory modules in Canidae and Hominidae

**DOI:** 10.1038/s41598-025-13034-y

**Published:** 2025-08-06

**Authors:** Jianhui Shi, Linting Wang, Lei M. Li

**Affiliations:** 1 State Key Laboratory of Mathematical Science, Academy of Mathematics and Systems Science, Chinese Academy of Sciences, Beijing, 100190 China; 2 School of Mathematical Sciences, University of Chinese Academy of Sciences, Beijing, 100049 China; 3 Center for Excellence in Animal Evolution and Genetics, Chinese Academy of Sciences, Kunming, 650223 China

**Keywords:** *Cis*-regulatory module, Saltation, Can-SINE element, Cognition, Emergence, Singular value decomposition, Evolution, Data integration, Data mining, Genome informatics, Comparative genomics, Genome evolution, Modularity

## Abstract

Dogs, which were segregated from wolves about thirty thousand years ago, show unique human-similar social-cognitive abilities. However, the genomic basis accounting for the phenotypic saltation between dog and wolf remains unclear. We performed a comparative analysis of genome-wide *cis*-regulatory element frequencies (CREF) for five canids: dog, dingo, red fox, dhole, and wolf, along with four hominids. For each species, genome-wide CREFs are organized into a matrix. The species-specific CREF matrix is stratified into multiple dual eigen-modules through robust singular value decomposition. Cross-species comparisons of dual eigen-modules demonstrated that the top three eigen-modules are highly conserved while the fourth and fifth ones underwent a saltation in dogs. The red fox is closest to the degenerate point characterizing the onset of saltation. Gene enrichment analysis and motif analysis revealed that myelination, long-term memory, and cochlear development are significantly enhanced at level four in both humans and dogs, but not in wolves. Cross-family comparisons revealed a more similar cognition-memory module between humans and dogs than between humans and chimpanzees. Not only the presence of *cis*-elements but also their frequencies are crucial for deciphering the regulatory saltations that characterize a striking convergent evolution of dogs and humans in proximal regulatory sequences.

## Introduction

Dogs have been humans’ best friends since their domestication. As domesticated descendants segregated from wolves about thirty thousand years ago^1,2^, dogs show unique human-similar abilities^3,4^. Advanced memory is a key ability they possess. In addition, they are able to understand human voice commands, to read and to react appropriately to human body language such as pointing and gesturing. Surprisingly, these unique social-cognitive abilities were found neither in wolves nor in other close relatives of humans such as great apes^5,6^. In other words, dogs have evolved cognitive abilities more similar to humans than our closest genetic relatives defined on protein-coding DNA sequences^7^.

It is important to understand the genomic basis accounting for the phenotypic differences between dogs and wolves as well as for the cognition similarity between dogs and humans. This similarity in social-cognitive phenotype cannot be explained by protein-coding DNA sequences, as comprehensive molecular phylogenetic analysis showed that humans and dogs are in different primary superordinal clades^8^. Of note, recently, the similarity between dogs and humans at the molecular level was demonstrated in the study of the spatial and temporal expression pattern of proteins in the brain^9^. To resolve the paradox, in this article, we switch from protein sequences to their regulatory sequences.

King and Wilson proposed that the major biological differences between humans and chimpanzees might be accounted for by mutations in regulatory sequences^10^. Moreover, it has been postulated that changes in *cis*-regulatory sequences constitute an essential part of the genetic basis for evolution and adaptation^11,12^. Indeed, we have reported that the phenotypic saltation between humans and apes, which could not be fully understood by protein sequences otherwise, could be explained by the *cis*-regulatory element frequencies (CREF) modules defined by regulatory sequences^13^. The fourth and fifth CREF modules underwent a saltation during the evolution from apes to humans^14^.

The phenomenon in physics analogous to evolutionary saltation is typically referred to as a phase transition, exemplified by the ferromagnetic transition at the Curie point. In the last century, physicists have employed statistical models and ensembles to characterize phase transitions, with the Ising model standing as a paradigm. Rigorous analyses have shown that phase transitions can occur in 2- or 3-dimensional Ising models, but not in the 1-dimensional case. Furthermore, such transitions necessitate a thermodynamically large particle ensemble. These insights inspired us to use the genome-wide CREF matrix as a basis for investigating regulatory saltation. The CREF matrix is 2-dimensional with tens of thousands of genes along the rows and over one thousand *cis*-elements as columns. Among the various mathematical approaches applied to the 2D Ising model, we adopted the degenerate eigenvalue method to characterize saltation phenomena in the CREF matrix^15,16^.

We are interested to know whether a similar saltation occurred in Canidae; if so, it is interesting to know whether dogs and humans share certain common genetic changes in the saltations. By an approach similar to the Hominidae study, we examine if the *cis*-regulatory sequences of Canidae species could explain their phenotypic differences.

In order to perform a comprehensive comparison in Canidae, we consider five canids: domestic dog, dingo, red fox, dhole, and gray wolf. Dingoes are wild canids native to Australia and originated from domestic dogs. Since the ancestors of dingoes went through a process of domestication followed by certain feralization, the dingo is a special model for studying the evolutionary and genomic mechanisms. Red foxes in Russian farm-fox experiments have been reported to be domesticated to some extent and become eager to interact with humans like dogs rapidly after over 40 generations^17^. The dhole is a canid genetically close to species within the genus Canis and native to Central, South, and Southeast Asia^18^.

Among the sources of *cis*-regulatory element variations, mutations related to transposable elements are a certain factor. However, the quantification of their influences is a challenging problem. Using the motifs present on Alu elements (MPA), we demonstrated that one key genetic driving force underlying the saltation of CREF modules is mutations related to Alu. A complete account of human-specific Alu insertions into proximal regulatory regions has been provided^13,15^. Inspired by the role of Alu in Hominidae, in this article, we examine the short interspersed nuclear elements (SINEs) in Canidae, namely Can-SINE elements. We introduce the notion of motifs present on Can-SINE elements and use their counts as an indirect yet robust measure of the transposons’ influence on CREFs. In addition, we further develop the CREF theory by introducing the notion of the polarization degree of an eigenvector. We demonstrate that the CREF module is a general model applicable to the study of regulatory evolution from Canidae to Hominidae.

## Results

### CREF matrix—a systematic representation of genome-wide *cis*-regulatory element frequencies

The focus of this study is the *cis–trans* regulation around the proximal regulatory regions of protein-coding genes. Instead of relying on the condition-specific binding data obtained from ChIP-seq experiments, we consider the binding strengths that involve solely DNA sequences. To quantitatively measure the *cis–trans* binding strength, we count CREFs. That is, we count the occurrences of *cis*-elements that can bind to a specific transcription factor in the proximal regulatory region of a protein-coding gene^13^. There are several motivations behind this definition (Supplementary Text, subsection "Motivations behind the definition of *cis*-regulatory element frequency"). We specifically consider the transcription factors whose binding motifs are available in the TRANSFAC database^19^.

For a certain species, the collection of the CREFs of all protein-coding genes can be arranged in a matrix, referred to as the CREF matrix. The rows and columns of the CREF matrix are the genes and motifs for each species, respectively. Here we construct species-specific CREF matrices for five canids: domestic dog, dingo, red fox, dhole, and gray wolf. For the sake of convenience, we will refer to the domestic dog as “dog” and the gray wolf as “wolf” hereafter. In the case of the dog, the CREF matrix includes 15,746 rows and 1403 columns.

In each row of a CREF matrix, the frequencies of all motifs cover the potential combinatorial interactions of all transcription factors. In other words, all the quantitative combinations of *cis*-regulatory elements are summed in the CREF matrix. An appropriate decomposition of the matrix was expected to reveal the quantitative *cis*-regulatory modules.

### Decomposition of CREF matrices into multiple dual eigen-modules

**Fig. 1:**
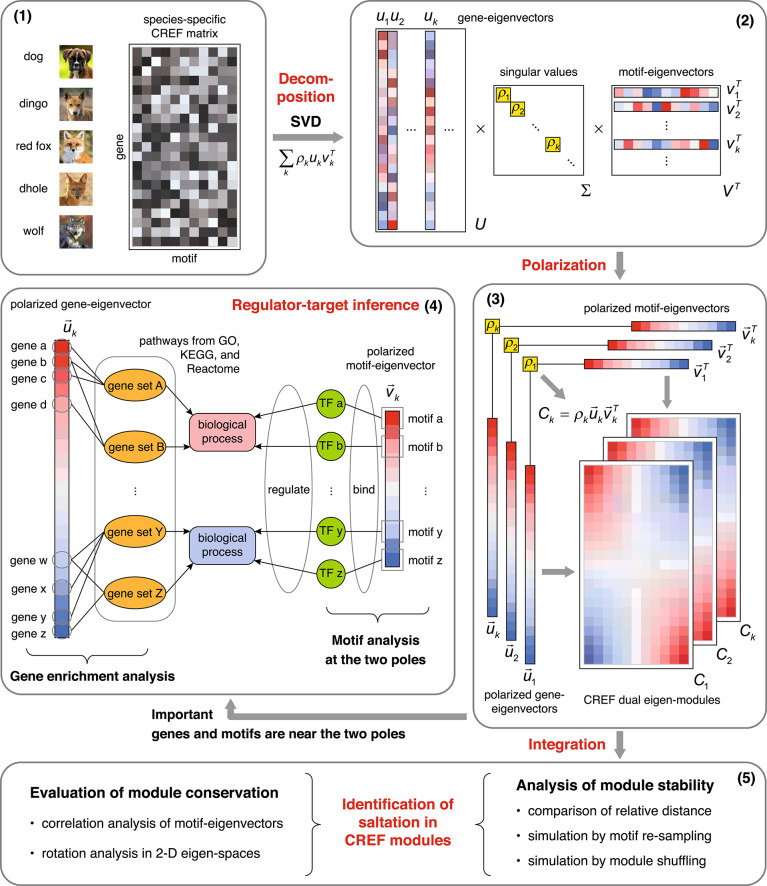
**The scheme of the CREF dual eigen-analysis.** (**1**) The species-specific *cis*-regulatory elements frequency (CREF) matrices of five canids: dog, dingo, red fox, dhole, and wolf. The CREF matrix is a systematic representation of the *cis–trans* binding strengths in the proximal regulatory region of all protein-coding genes. (**2**) The singular value decomposition (SVD) of the CREF matrix. The CREF matrix is stratified into multiple levels. Each level has a singular value, a gene-eigenvector, and a motif-eigenvector. (**3**) Polarization of eigenvectors by sorting their loadings. Each pair of polarized gene- and motif-eigenvectors, together with the singular value comprise a CREF dual eigen-module. (**4**) Inferring the regulator-target of each CREF dual eigen-module by enrichment analysis on the polarized gene-eigenvectors and motif analysis on the polarized motif-eigenvectors. (**5**) Identification of the saltation by the integration of CREF modules. The integration mainly consists of the evaluation of module conservation and analysis of module stability.

To dissect the underlying regulatory structure of the high-dimensional CREF matrices, we apply the dual eigen-analysis, as illustrated in Fig. 1. This approach enables us to identify functional modules and evaluate their conservation in three major steps shown below.

First, we compute the robust singular value decompositions (SVD) of the CREF matrices^20^. The robust version of SVD is adopted to remove or reduce the influence of any possible outliers in the matrix. This process yields a singular value, a gene-eigenvector, and a motif-eigenvector at each level. The gene-eigenvector is composed of gene loadings that represent the weights of the genes; the motif-eigenvector is composed of motif loadings, or weights.

Second, we polarize each pair of gene- and motif-eigenvectors by sorting their loadings. Each pair of polarized gene- and motif-eigenvectors, together with the singular value, comprises a dual eigen-module of CREFs, referred to as a CREF dual eigen-module. They stratify the system of CREFs into different levels from high to low, as indicated by singular values. In the CREF dual eigen-module at a certain level, the most prominent genes and their corresponding regulatory *cis*-motifs can be found near the two poles of the eigenvectors.

Third, we compare each CREF eigen-module across five canids and integrate the analyses. We first evaluate the conservation of modules by analyzing the correlation between the motif-eigenvectors and their rotations in subspaces. Next, we assess the stability of modules by comparing relative distances and conducting mathematical simulations. Furthermore, we infer the regulator-target relationships for each CREF eigen-module. Through gene enrichment analysis, we identify the biological processes enriched at the two ends of the polarized gene-eigenvectors. Subsequently, the dual analysis finds the *cis*-elements that regulate these biological processes at the two ends of the coupled polarized motif-eigenvectors. Finally, by integrating these analyses, we identify the saltation in CREF modules.

### Comparison of singular values of CREF dual eigen-modules across five canids

The *i*-th singular value of a CREF matrix is the Frobenius norm of the *i*-th CREF eigen-module. It reflects the frequency level of the *cis*-regulatory element at the *i*-th level. We compare the singular values of CREF dual eigen-modules across five canids in the following two ways.

First, we compare the normalized singular values, obtained by dividing the total sum, at different levels. Fig. S1A and Table S1 show the top nine singular values, their percentages, and the cumulative percentages. For all five canids, the distributions of their singular values exhibit long tails. The top CREF eigen-modules are expected to capture the principal information of the CREF matrix. In this article, we focus on the top nine modules.

Second, we compare the relative distance between adjacent singular values. This is needed in the sensitivity analysis of eigenvectors (Supplementary Text, subsection "Sensitivity of eigenvalues and eigenvectors"). Fig. S1B and Table S2 display the relative distance between adjacent singular values up to the ninth level. We observe that in dogs (Boxer), red foxes, and dholes, the relative distance between the fourth and fifth levels is remarkably small. According to the matrix perturbation theory, the fourth and fifth CREF eigen-modules are not as stable as those at other levels. This is further supported by the subsequent simulations. As shown later, the fourth and fifth modules underwent a saltation in Canidae.

### Conserved and divergent modules identified by correlation analysis of motif-eigenvectors

Along the motif-eigenvectors, we use the Pearson correlation coefficient (PCC) as a quantitative measure to evaluate the conservation of the CREF eigen-modules of two species at a certain level. It is noted that the square of the PCC, denoted by ${r}^{2}$, equals the coefficient of determination, denoted by ${R}^{2}$. The latter quantifies the predictability of one eigenvector from the other. As the PCC approaches one, the coefficient of determination also approaches one, indicating that the two eigenvectors are more conserved, as they can better predict each other.

**Fig. 2:**
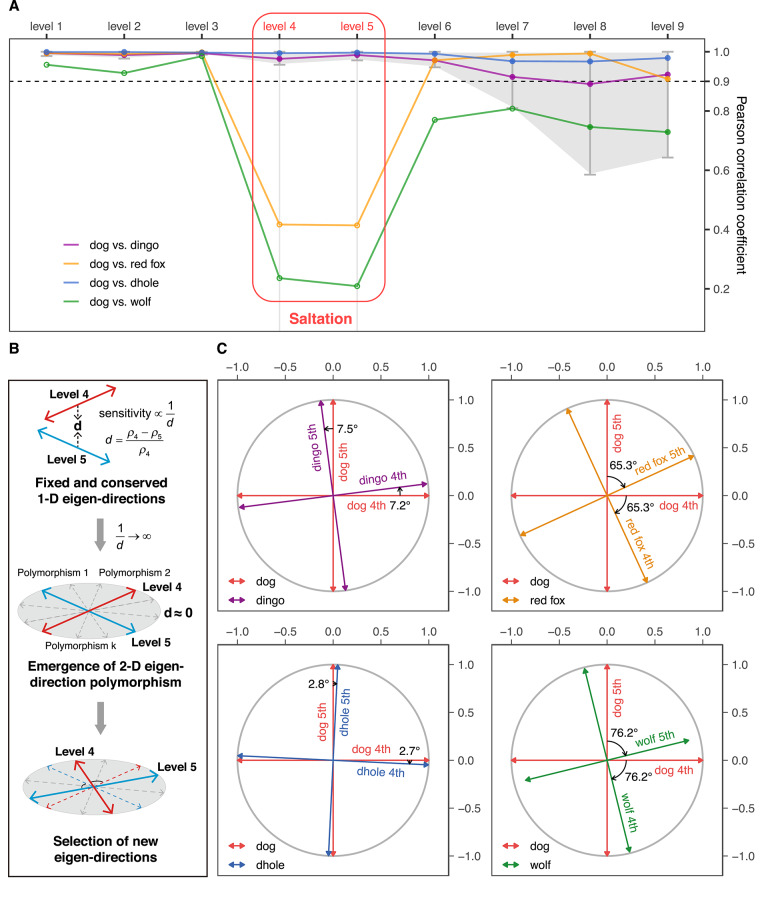
(**A**) **The line chart of PCCs (Pearson correlation coefficients) between dogs’ top nine motif-eigenvectors and those of the other four canids. **The thresholds of conservation at each level are indicated by gray shading. The top nine motif-eigenvectors of dingoes and dholes are highly correlated with those of dogs, suggesting their top nine modules are highly conserved. In contrast, the PCCs between red foxes, wolves and dogs are significantly decreased to below 0.5 at the fourth and fifth levels, indicating a divergence in these two modules. This divergence in the fourth and fifth modules characterizes a saltation in Canidae. (**B**) **Schematic representation of the saltation in the CREF modules.** As mutations accumulate in the regulatory sequences, the relative distance between adjacent singular values approaches zero, and the sensitivity approaches infinity. Consequently, a 2-D eigen-direction polymorphism—represented by the shaded disk—emerges, as shown in the middle. (**C**) **Rotations between the fourth and fifth motif-eigenvectors from dogs to the other four canids. **The direction and degree of rotation are marked beside each angle. Except for wolves, the other three canids and dogs have almost identical 2-D eigenspaces. In the cases of dingoes, red foxes, and dholes, the rotation angle of the fourth motif-eigenvector is approximately equal to that of the fifth one. Large rotations in red foxes and wolves indicate a saltation in the evolution of *cis*-regulatory modules in Canidae.

Figure 2A shows the PCCs between the top nine motif-eigenvectors of dogs and those of the other four canids, together with thresholds of conservation. These thresholds are set up to determine whether CREF eigen-modules are highly conserved or divergent (Supplementary Text, subsection "PCC thresholds of conservation when comparing motif-eigenvectors" and Fig. S2). When comparing the PCCs against the thresholds, a similar pattern is observed in dingoes and dholes. That is, all top nine modules between each of them and dogs are highly conserved. In the case of red foxes, although the other seven modules exhibit high conservation, the fourth and fifth levels display significantly lower PCCs than the threshold. Therefore, the fourth and fifth modules demonstrate the divergence between red foxes and dogs. In the case of wolves, the PCC is greater than 0.90 at the top three levels. Therefore, we consider the top three modules to be relatively conserved between wolves and dogs. Their PCCs at the fourth and fifth levels are significantly lower than the threshold compared to those between red foxes and dogs.

The reliability of this comparison of motif-eigenvectors is enhanced by the results of multiple breeds of dogs and two dingoes (Supplementary Text, subsection "Comparison along motif-eigenvectors including multiple breeds of dogs", Fig. S3, Fig. S4, and Fig. S5).

The contrast of the motif-eigenvector PCCs at the fourth/fifth levels and those at the top three together with the sixth levels, reveals the evolution of the CREF modules among canids. The divergence in the fourth and fifth modules characterizes a saltation in Canidae, similar to that reported in the Hominidae study^13^.

Figure 2B depicts a schematic representation of the saltation in the CREF modules. The sensitivity of a CREF eigen-module is inversely proportional to the relative distance between adjacent singular values. Take the dog/wolf and the fourth/fifth CREF modules as an example. Before the split from the wolf lineage, the fourth and fifth motif eigen-directions of the dog’s ancestor were relatively fixed and conserved compared to those of the wolf’s ancestor, as shown at the top. As mutations accumulated in the regulatory sequences, the distance between the two singular values approached zero, driving the sensitivity to approach infinity. This triggered the emergence of a 2-D degenerate eigenspace—represented by the shaded disk, exhibiting 2-D eigen-direction polymorphism, as shown in the middle. In other words, any eigen-direction within this 2-D space represented a polymorphism. Eventually, a new direction was selected and fixed in the lineage leading to the dog, as shown at the bottom.

### Rotation in the 2-D eigenspace spanned by the fourth and fifth motif-eigenvectors

To investigate the relationships between the fourth and fifth motif-eigenvectors in the five canids, we analyze the 2-D eigenspace spanned by these two vectors. We orthogonally project the fourth and fifth motif-eigenvectors of the other four canids onto the 2-D eigenspace of dogs.

As depicted in Fig. 2C, except for wolves, the 2-D eigenspaces of the other three canids and dogs are almost identical. This can be seen from two observations. First, the projections of both the fourth and fifth motif-eigenvectors almost reach the circumference, thereby no leakage to other eigenvectors. Second, the angle between the projections is roughly 90°, namely, the rotation angle of the fourth motif-eigenvector is approximately equal to that of the fifth one.

The rotation angles and directions of the four canids are not the same. The rotation angles of red foxes and wolves (more than 65°) are significantly larger than those of dingoes and dholes (less than 8°). In the cases of red foxes and wolves, the fourth and fifth motif-eigenvectors are closer to the fifth and fourth ones of dogs, respectively. This suggests that a jump between eigen-modules occurred during their evolution. Moreover, if we consider the rotation direction and take dogs as the reference position as before, the fourth and fifth motif-eigenvectors of dingoes rotate counterclockwise in the first subplot of Fig. 2C, in the opposite direction to those of red foxes, dholes, and wolves, shown in the other three subplots. This implies that the evolution of dingoes somehow took a different path from the other three canids. These results are further supported by the eigenspaces of multiple breeds of dogs and two dingoes (Supplementary Text, subsection "Rotations in the 2-D eigenspaces including multiple breeds of dogs", Fig. S6, and Fig. S7).

Large rotations in red foxes and wolves do imply a saltation in the evolution of *cis*-regulatory modules in Canidae.

### Important genes and motifs are near the two poles

We proceed to biologically interpret the genes and motifs in CREF eigen-modules. In each module, the loadings in the polarized eigenvectors reflect the biological importance of genes and motifs. We define the square of these loadings as the energy associated with genes and motifs. A larger loading or energy indicates a more important role of a gene or a motif. This definition takes into account two things. On the one hand, taking the square eliminates the sign of the loading. On the other hand, each eigenvector is normalized, and so is the energy.

Fig. S11 illustrates the cumulative distributions of the energy of the genes and motifs in the top nine polarized eigenvectors of five canids. In both genes and motifs, the majority of the energy is concentrated at the two poles of the polarized eigenvectors, albeit with varying degrees. This uneven pattern demonstrates that in each CREF dual eigen-module, genes and motifs with important biological functions are near the two poles of the eigenvectors. In the subsequent sections, we will describe the biological functions and corresponding regulatory relationships among these genes and motifs at the poles.

### A relatively small number of motifs control the transcription of many genes

With a close look at Fig. S11, we found that compared with genes, the cumulative distribution curve of motifs exhibits a steeper slope at the two poles. This observation indicates that the motifs have higher energy concentrations than genes do.

Quantitatively, we define a metric, polarization degree, to measure the unevenness or concentration degree of each eigenvector. The mathematical definition of polarization degree is given in the section "Polarization degree of eigenvectors", Supplementary Text. We compute the polarization degree of the top nine gene- and motif-eigenvectors for five canids (Table S3). The range of the polarization degree of gene-eigenvectors is from 0.669 to 0.807, whereas that of motif-eigenvectors is from 0.792 to 0.915. The distribution pattern shown in Figure S17 indicates that in general, the polarization degree of motif-eigenvectors is higher than that of gene-eigenvectors.

The higher concentration of motifs at the poles reveals that in each CREF dual eigen-module, a small portion of *cis*-regulatory elements control the transcription of a relatively larger portion of genes.

### Genes relating to long-term memory cluster near the positive pole of the human and dog fourth gene-eigenvectors

Dogs, known as man’s best friends, fulfill a variety of essential roles for humans, such as pets, protection, herding, hunting, searching, and assistance. These important roles cannot be achieved without the dogs’ loyalty, social cognition, and ability to learn. Long-term memory, as the foundation of these special abilities, enables dogs to remember, accompany, and remain loyal to their owners for a long time, making them the dearest friends of humans. Moreover, experimental evidence from a study involving two dogs demonstrated their capability of learning by inference and advanced memory skills^21,22^.

**Fig. 3:**
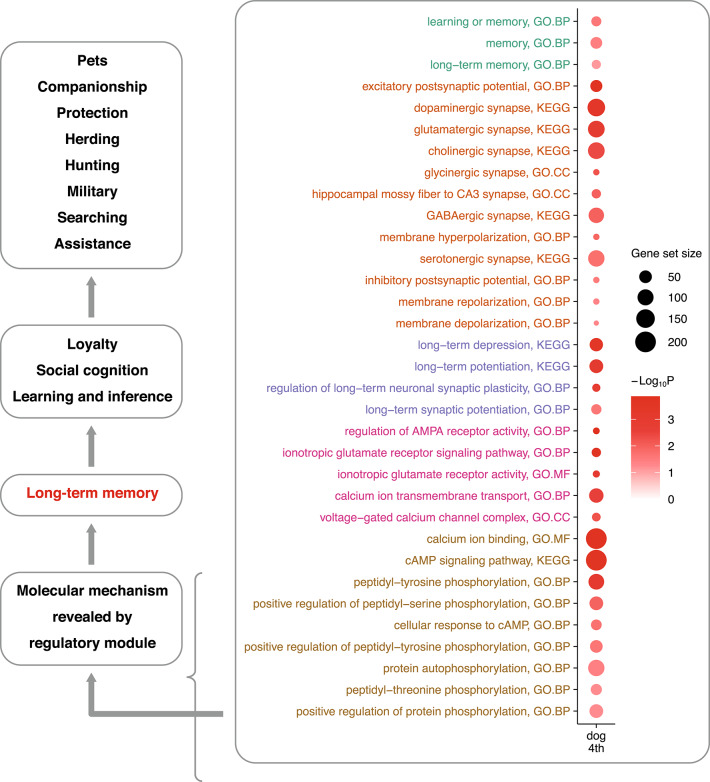
**Gene enrichment at the positive pole of the dog fourth eigenvector. **The up-regulated genes are integrated into molecular pathways centering around long-term memory that can explain, to a great extent, the phenotypical behaviors of dogs. According to their biological relevance, the enriched gene subsets are grouped from top to bottom by long-term memory, neurotransmission, synaptic plasticity, LTP induction, and other LTP mechanisms, shown in different colors.

In this study, enrichment analysis reveals an up-regulation of long-term memory at the positive pole of the dog fourth gene-eigenvector. This aligns closely with the up-regulation of long-term memory reported in humans^13^. The method of the enrichment analysis is explained in detail in the Supplementary Text, subsection "Enrichment analysis by the Wilcoxon rank sum scoring method". Several specific biological pathways related to long-term memory are displayed in Fig. 3. A detailed list of enriched gene subsets is shown in Table S4. The biological explanation of these enriched gene subsets can be found in the Supplementary Text, subsection "Details of the long-term memory-related pathways significantly enriched at the positive pole of the fourth gene-eigenvector".

These molecular discoveries can explain some remarkable social-cognitive abilities observed in dogs, such as understanding human language, gestures, and facial expressions, as well as communicating with us. These abilities are neither possessed by dogs’ closest canine relatives nor by other highly intelligent mammals including great apes^5,6^. The differences in their cognitive phenotypes are consistent with the discoveries of the biological pathways in the fourth CREF module. On the one hand, long-term memory-related pathways at the poles of the fourth gene-eigenvector are less significant in the other four canids than those in dogs. On the other hand, as a special phenotype that lays the foundation for human uniqueness, long-term memory is up-regulated at the positive pole of the human fourth gene-eigenvector, but not along the fourth gene-eigenvectors of two apes, chimpanzees and orangutans^13^.

### Myelination genes cluster near the poles of human and dog fourth gene-eigenvectors

Myelin is critical for the proper function of the vertebrate nervous system. It is recently reported that myelination also contributes to memory consolidation and recall^23^. Composed of sheath segments, it serves as a lipid-rich and multilamellar insulating structure of nerve cell axons in both the central (CNS) and peripheral nervous systems (PNS). The myelin sheath enables faster and energetically more efficient conduction of action potentials along axonal fibers, while also providing physical and trophic support to neurons. White matter, which mainly consists of myelinated axons, is reported to be relatively abundant in the brains of both humans and dogs in a recent study^9^. Furthermore, it was reported that dogs have more myelin proteins than mice in the hippocampus and prefrontal cortex, and there is a significant correlation in myelin proteins between humans and dogs^9^.

**Fig. 4:**
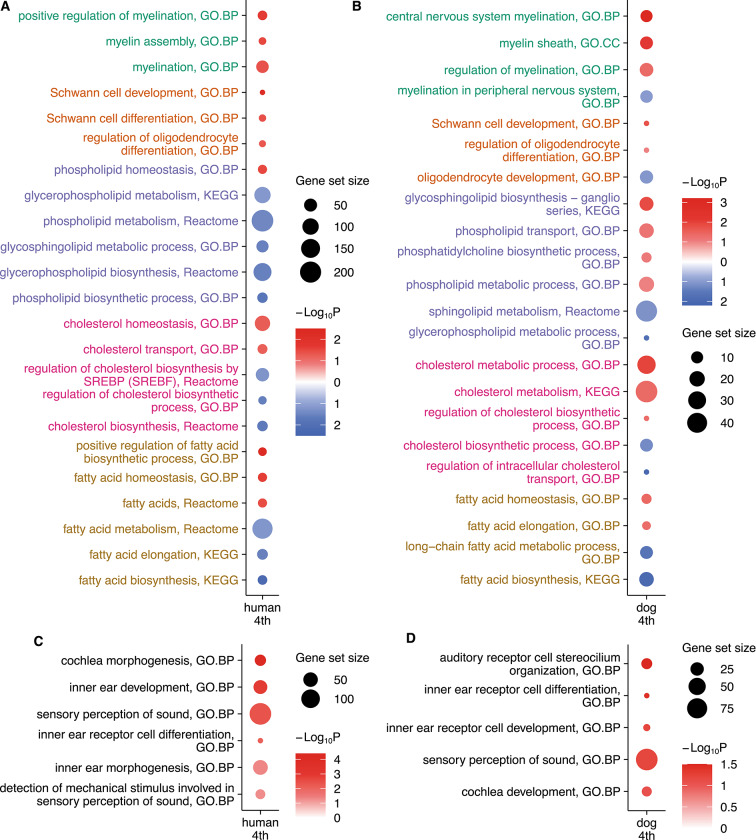
**The comparison of the human and dog gene-eigenvectors at the fourth and fifth levels.** (**A**) Significant myelination-related gene subsets enriched at the poles of the human fourth polarized gene-eigenvector. According to their biological relevance, the enriched gene subsets are grouped from top to bottom by myelination, myelin-forming cell, myelin lipid metabolism, cholesterol metabolism, and fatty acid metabolism, shown in different colors. (**B**) Significant myelination-related gene subsets enriched at the poles of the dog fourth polarized gene-eigenvector. The grouping categories and colors are the same as in (**A**). (**C**) Significant cochlea development-related gene subsets enriched at the poles of the human fourth polarized gene-eigenvector. (**D**) Significant cochlea development-related gene subsets enriched at the poles of the dog fourth polarized gene-eigenvector.

In this study, we observed an enhancement of myelination at the poles of both human and dog fourth gene-eigenvectors. Figure 4A,B illustrate several specific biological pathways related to myelination, including CNS and PNS myelination, regulation of myelination, myelin assembly, and myelin sheath. Detailed lists of enriched gene subsets in humans and dogs are respectively shown in Table S5 and Table S6. The biological explanation of these enriched gene subsets can be found in the Supplementary Text, subsection "Details of the myelination-related pathways significantly enriched at the poles of the fourth gene-eigenvector". Besides, we found the *MBP* gene ranking 271/15,746 at the pole of the ninth gene-eigenvector in dogs. The protein encoded by the *MBP* gene serves as a primary component of the myelin sheath in the CNS and PNS.

### Cochlea development genes cluster near the poles of the human and dog fourth gene-eigenvectors

The communication between humans and dogs involves the utilization of various modalities, including hand signals, body posture, and vocalization. Trained dogs display an impressive capability to comprehend basic human languages such as simple commands and spoken words. The comprehension of these diverse auditory signals relies on dogs’ ability to sense and distinguish sounds. In mammals, the transduction of auditory signals, such as acoustic waves, takes place in the cochlea, which serves as a vital component of the inner ear.

Our enrichment analysis reveals a significant enhancement of cochlea development at the poles of both human and dog fourth gene-eigenvectors. Several significant biological pathways related to cochlea development are shown in Fig. 4C,D, respectively for dogs and humans. Cochlea morphogenesis, inner ear morphogenesis, and inner ear development are enriched in humans; cochlea development is enriched in dogs. Detailed lists of enriched gene subsets in humans and dogs are shown in Table S7 and Table S8, respectively. The biological explanation of these enriched gene subsets can be found in the Supplementary Text, subsection "Details of the cochlea development-related pathways significantly enriched at the poles of the fourth gene-eigenvector".

### Long-term memory regulators stand around the positive pole of the human and dog fourth polarized motif-eigenvectors

According to the CREF dual eigen-module structure, the *cis*-elements located at the positive pole of the polarized motif-eigenvector and their binding factors are in the positions that regulate the biological processes enhanced at the positive pole of the polarized gene-eigenvector; likewise, the *cis*-elements located at the negative pole of the polarized motif-eigenvector and their binding factors are in the positions that regulate the biological processes enhanced at the negative pole of the polarized gene-eigenvector. The correspondence is illustrated by some examples as follows.

**Fig. 5:**
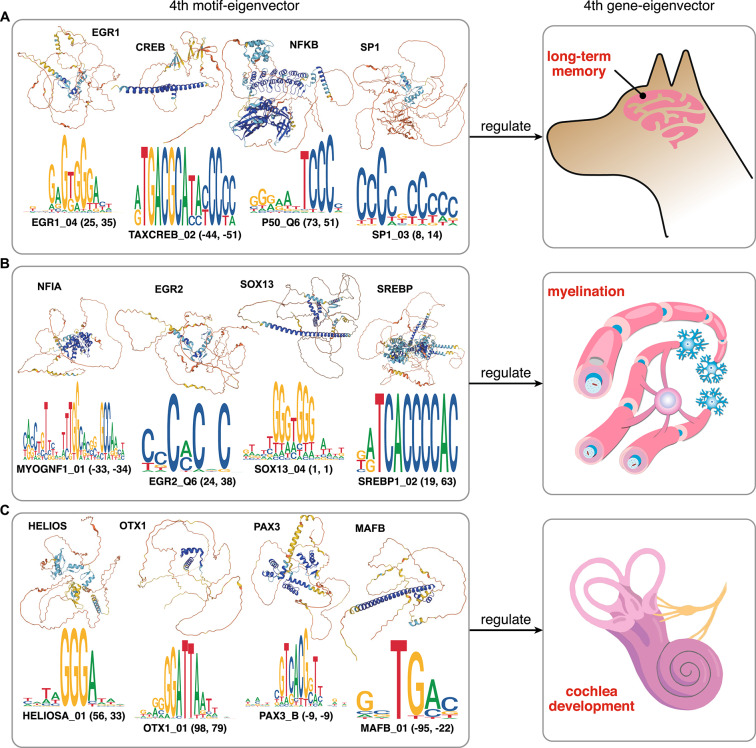
**Examples of the** ***cis*–*trans*** **regulatory correspondence between the polarized motif- and gene-eigenvectors.** These examples are directly obtained from the human and dog CREF modules, and are supported by experimental evidence found in the literature. In each row, the right box displays certain major biological processes enriched at the poles of the fourth polarized gene-eigenvector; whereas the left box illustrates their transcription factors and binding *cis*-regulatory motifs near the poles of the fourth polarized motif-eigenvector of both humans and dogs. The two numbers in parentheses after the motif symbol indicate its rank in the fourth motif-eigenvector of humans and dogs, respectively. Motifs positioned at the negative pole are marked by negative ranks. The protein structures are from the AlphaFold Protein Structure Database^41,42^. (**A**) Long-term memory and its four transcription factors with binding motifs. (**B**) Myelination and its four transcription factors with binding motifs. (**C**) Cochlea development and its four transcription factors with binding motifs.

Unlike short-term memory, the establishment of long-term memory relies on a temporally limited phase of RNA and protein synthesis^24^. A large number of important transcription factors involved in the regulation of long-term memory have been identified. They include EGR1, NFKB, CREB, MAZ, SP1, SP3, and SP4. We observed that nearly all of the *cis*-elements of these transcription factors are positioned at the positive pole of both human and dog fourth motif-eigenvectors, corresponding to the up-regulation of long-term memory at the positive pole of their fourth gene-eigenvectors. The details of these transcription factors are available in the Supplementary Text, subsection "Details of the long-term memory regulators standing around the positive pole of the fourth motif-eigenvector". Four of the long-term memory motifs and their binding factors are illustrated in Fig. 5A.

We note that in the fourth motif-eigenvectors of wolves and three apes, almost all *cis*-binding elements of transcription factors mentioned above rank out of the top 100. These *cis*-element results are in line with their regulatory targets observed along gene-eigenvectors.

### Dual eigen-analysis identifies the regulators involved in myelination at the fourth level for both human and dog

With a close look at the top of the fourth polarized motif-eigenvector of humans and dogs, we find a list of high-ranking motifs whose binding factors play important roles in the myelination process. Their binding factors include NFIA, SOXD, PAX3, AP-2, NFKB, EGR2, and SREBP. The details of these transcription factors are available in the Supplementary Text, subsection "Details of the myelination regulators standing around the poles of the fourth motif-eigenvector". Four of them are shown in Fig. 5B. Besides, we find SOX10_01 ranking 207 at the pole of the ninth motif-eigenvector in dogs. Its binding factor SOX10 was reported to be able to stimulate the transcription of the gene *MBP*, a key myelin component, by directly binding its proximal promoter region^25^. This regulatory-target relation further supports the correspondence of the gene- and motif-eigenvectors.

### *Cis–trans* regulation of cochlea development in the human and dog fourth CREF eigen-modules

Dual eigen-analysis reveals the *cis–trans* regulation of cochlea development. Indeed, important transcription factors involved in cochlea development, such as PKNOX2, HELIOS, OTX1, PAX3, and MAFB, have binding elements positioned at the poles of both human and dog fourth motif-eigenvectors. The details of these transcription factors are available in the Supplementary Text, subsection "Details of the cochlea development regulators standing around the poles of the fourth motif-eigenvector". Four of them are shown in Fig. 5C.

### The evolution diagram of the Canidae fourth and fifth CREF eigen-modules

**Fig. 6:**
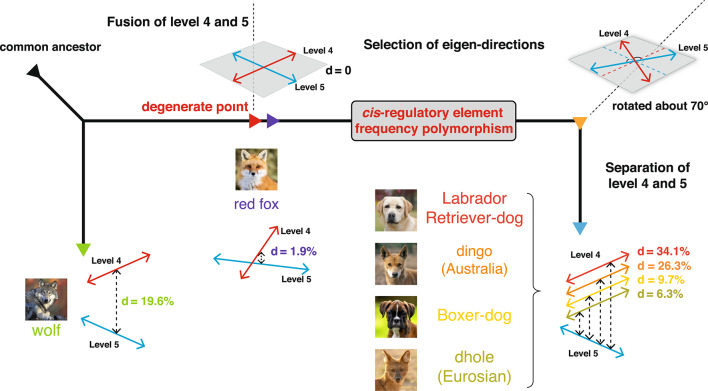
**The CREF eigen graph of the evolution of Canidae fourth and fifth CREF eigen-modules. **After the split of the common ancestor from the wolf lineage, the dog lineage experienced a more rapid decrease in the relative distance between the fourth and fifth levels than the wolf lineage. At a specific point, the singular values of two levels became nearly identical and consequently, the two 1-D eigenvectors fused into a 2-D eigen-space. This degenerate point characterizes the onset of saltation. Notably, the red fox is near this degenerate point with a relative distance of only 1.9% between the fourth and fifth levels. In this 2-D eigen-space, any direction was an eigenvector. We refer to this 2-D eigen-space at the degenerate point as a space of CREF-polymorphisms. Then the selection occurred and only the eigen-direction with the highest fitness was eventually selected. After the fixation of the eigen-direction in the dog lineage, the two levels separated again. In comparison to the fourth and fifth motif eigenvectors of the red fox, those of dog, dingo, and dhole were rotated about 70°. Although dingo and dhole evolved in two different continents, they exhibited a strikingly similar rotation in the fourth and fifth CREF modules. The comparison of the relative distance from the degenerate point indicates that both the dog and the dingo are farther away from the degenerate point than the dhole.

Although the CREF eigen-module of a species is a static picture of its genome, it is quite stable when the adjacent singular values are distant. Roughly speaking, the interference between two adjacent eigenvectors is inversely proportional to the relative distance of the singular values. By comparing and connecting the fourth and fifth CREF eigen-modules of the five canids, we can infer their evolutionary path as shown in Fig. 6. After the split of the common ancestor from the wolf lineage, the dog lineage experienced a more rapid decrease in the relative distance between the fourth and fifth levels than the wolf lineage.

At a specific point, the singular values of two levels became nearly identical, resulting in the fusion of the two 1-D eigenvectors into a 2-D eigen-space. This degenerate point characterizes the onset of saltation. Notably, the red fox is near this degenerate point with a relative distance of merely 1.9% between the fourth and fifth levels. We will discuss in greater detail the phenomenon of red fox nearing the degenerate point in the next section.

In this 2-D eigen-space, any direction is an eigenvector. We refer to this 2-D eigen-space at the degenerate point as a space of CREF-polymorphisms. This space of CREF-polymorphisms allowed the selection to occur, and only the eigen-direction with the highest fitness was eventually selected. As previously shown by the gene enrichment and motif analysis, the dog-specific phenotypes including long-term memory, myelination, and cochlea development were indeed selected in the dogs’ fourth module.

After the selection and fixation of the eigen-direction in the dog lineage, the two levels separated again. The selected eigen-directions can be expressed by a continuous angle of rotation relative to the eigenvectors of the red fox, the species near the degenerate point. In comparison to the fourth and fifth motif eigenvectors of the red fox, the corresponding ones of dog, dingo, and dhole rotated about 70°. These significant rotations are further supported by the result of the projection of four breeds of dogs onto the red fox (Fig. S12). Among the dog breeds, the Boxer, which has the smallest relative distance from the degenerate point, and the Labrador Retriever, which has the largest, were picked to be shown in Fig. 6.

### The red fox is closest to the degenerate point characterizing the onset of saltation

In the Russian farm-fox experiment, for over forty generations, selective breeding for positive responses toward humans has resulted in the development of a partially domesticated strain of foxes that are eager to interact with humans like dogs^17^. Such surprising experimental results can be partially explained by our theory. The red fox is closest to the degenerate point characterizing the onset of saltation.

We propose that the red fox is near the degenerate point of saltation. When approaching the degenerate point, the fourth and fifth singular values get close to each other and lead to a high sensitivity of the CREF eigen-modules at these two levels. The sensitivity of the CREF eigen-modules is explained in detail in the Supplementary Text, subsection "Sensitivity of eigenvalues and eigenvectors". The fourth and fifth eigenvectors become wobbly and easier to be rotated or mutated. The resulting polymorphic eigen-directions are consequently subject to selection. Eventually, only the eigen-direction with the highest fitness was selected. Therefore, we suggest that the rapid domestication of red foxes is due to the CREF module structure in which the fourth and fifth eigenvectors are near the degenerate point of saltation.

This hypothesis is supported by several observations and simulations detailed in the Supplementary Text, subsections "Evidence supporting the hypothesis of the saltation near the degenerate point" and "Stability analysis of CREF eigen-modules by random shuffling". In one simulation, we derive the sampling distribution of singular values by motif re-sampling without replacement, *cf*. Fig. S8. In another, we randomly shuffle the loadings in the gene-eigenvector and motif-eigenvector that constitute the eigen-module at a given level. A new CREF matrix is then reconstructed and decomposed. We then compute the mean absolute values of the PCCs between the original motif-eigenvectors in the top nine eigen-modules and those obtained after random shuffling, *cf*. Fig. S9. We also compute the mean Frobenius norms between the original eigen-module matrices and those obtained after random shuffling, *cf*. Fig. S10.

### The similarity of the fourth and fifth modules suggests a parallel evolution between dingo and dhole

The ancestors of dingoes can be traced back to domestic dogs in southern East Asia at least 3500 years ago^26,27^. They migrated through Island Southeast Asia, eventually reached Australia, and subsequently diverged into a phenotypically and genetically distinct population of feral dogs^28^. In comparison, dholes have a wide distribution range that spans across Central, South, and Southeast Asia, and much of Europe.

Although dingoes and dholes have distinct evolutionary histories and geographic distributions, they share striking similarities in their *cis*-regulatory modules. Overall, as illustrated in Fig. S13, all of their top six pairwise motif-eigenvectors are highly correlated, indicating that these six modules are highly conserved between dingoes and dholes. In particular, when projecting their fourth and fifth motif-eigenvectors onto those of red foxes, both dingoes and dholes exhibit large rotations (about 62° and 72°), with consistent directions (Fig. S12). The similarities suggest an intriguing and surprising case of parallel evolution in the gene regulation of dingoes and dholes.

Nevertheless, certain differences in CREF eigen-modules are observed between dingoes and dholes. For instance, the comparison of the relative distance between adjacent levels shows that the distance between the fourth and fifth levels of dingoes is larger than that of dholes (Table S2), indicating that the rotation of eigen-modules occurred earlier in dingoes than in dholes.

### Motifs present on Can-SINE elements underlying the CREF eigen-module saltation

We now explore the genetic driving factors for the saltation in Canidae CREF eigen-modules. Point mutations typically occur and accumulate at a relatively slow pace, making it difficult to explain the sudden and rapid phenotypic changes observed near the saltation. An alternative explanation could involve mutations related to transposable elements.

In this section, we explore the relationship between Can-SINE elements and the saltation of Canidae CREF eigen-modules. The motivations are available in the Supplementary Text, subsection "Motivations for studying Can-SINE elements". Specifically, we examine how many motifs present on Can-SINE elements (MPCS) occur at the top of polarized motif-eigenvectors.

To do this, we need a comprehensive list of the MPCSs. First, we collected 18 Can-SINE consensus sequences from the Repbase database^29^ which cover major Can_SINE “CF” sub-types in the dog genome. Second, we use the MATCH program to identify *cis*-elements present on Can-SINE elements. To ensure both the reliability and the coverage of the MPCSs list, we take the threshold option "minSUM (minimize the sum of both errors)". Finally, the MATCH program output 97 MPCSs.

**Fig. 7:**
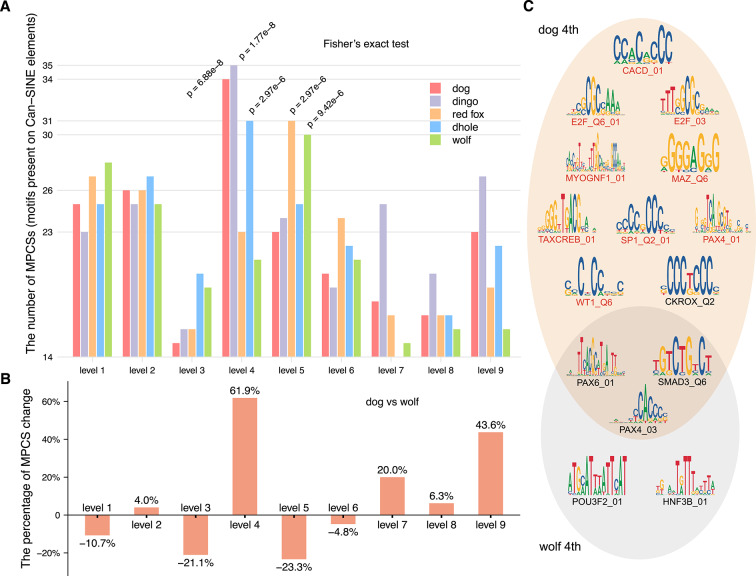
(**A**) **The number of MPCSs (motifs present on Can-SINE elements) in the top 100 motifs at each level of five canids.** Fisher’s exact test is performed to compare the odds of MPCS occurrences. The five significant *p*-values above the bar chart indicate that the MPCS occurrences increase significantly at level four in dogs, dingoes, and dholes while they do so at level five in red foxes and wolves. (**B**) **The relative change of MPCSs in percentages at each level from wolf to dog.** The number of MPCSs increases most prominently at level four by 61.9%, followed by 43.6% at the ninth level. (**C**) **The Venn diagram of MPCSs in the top 100 motifs in the fourth eigenvectors of dogs and wolves.** The result is based on the MPCSs output by the MATCH program with the minFP option. Nine highlighted MPCSs rank at the top of the fourth motif-eigenvector in both humans and dogs, but do not in wolves. They include four motifs of important transcription factors involved in myelination and long-term memory, MAZ_Q6, MYOGNF1_01, SP1_Q2_01, and TAXCREB_01.

Based on this MPCSs list, we quantitatively measure the impact of Can-SINE elements on the *cis–trans* regulation. In each of the five canids, we count the number of MPCSs among the top 100 motifs at the two poles of each level, as illustrated in Fig. 7A.

We compare the occurrences of MPCSs and other motifs among the top 100 of the motif-eigenvector by Fisher’s exact test (one-sided)^30,31^. Details of the comparison are given in the Supplementary Text, subsection "Frequency comparison of MPCS and other motifs at the poles of the fourth and fifth motif-eigenvectors" and Table S10. The result does exhibit some differences between the odds of occurrences of MPCSs and other motifs. At level four, it is obvious that the odds of MPCS occurrences increase substantially for dogs, dingoes, and dholes, with *p*-values 6.88e-8, 1.77e-8, and 2.97e6 respectively (Fig. 7A). While at level five, MPCS occurrences of red foxes and wolves are significantly enhanced with *p*-values 2.97e-6 and 9.42e-6.

In particular, we calculate the relative change of MPCSs in percentages at each level from wolves to dogs, as shown in Fig. 7B. It is obvious that the number of MPCSs increases most prominently at level four by 61.9%, followed by 43.6% at the ninth level.

Then we examine the specific list of MPCSs in the top 100 of dog and wolf fourth motif-eigenvectors. To obtain the representative MPCSs that fit in the graph, we performed this analysis with the MATCH threshold option set to "minFP (minimize false positive rate)" (Supplementary Text, subsection "MPCSs at the top of polarized motif-eigenvectors with the MATCH program option set to minFP"). Ten MPCSs are found to rank among the top 100 of the fourth motif-eigenvector of dogs, but not wolves (Fig. 7C). Interestingly, among these ten MPCSs, nine are at the top of the human fourth motif-eigenvector as well.

### CREF analysis shows a more similar cognition module between humans and dogs than that between humans and chimpanzees

**Fig. 8:**
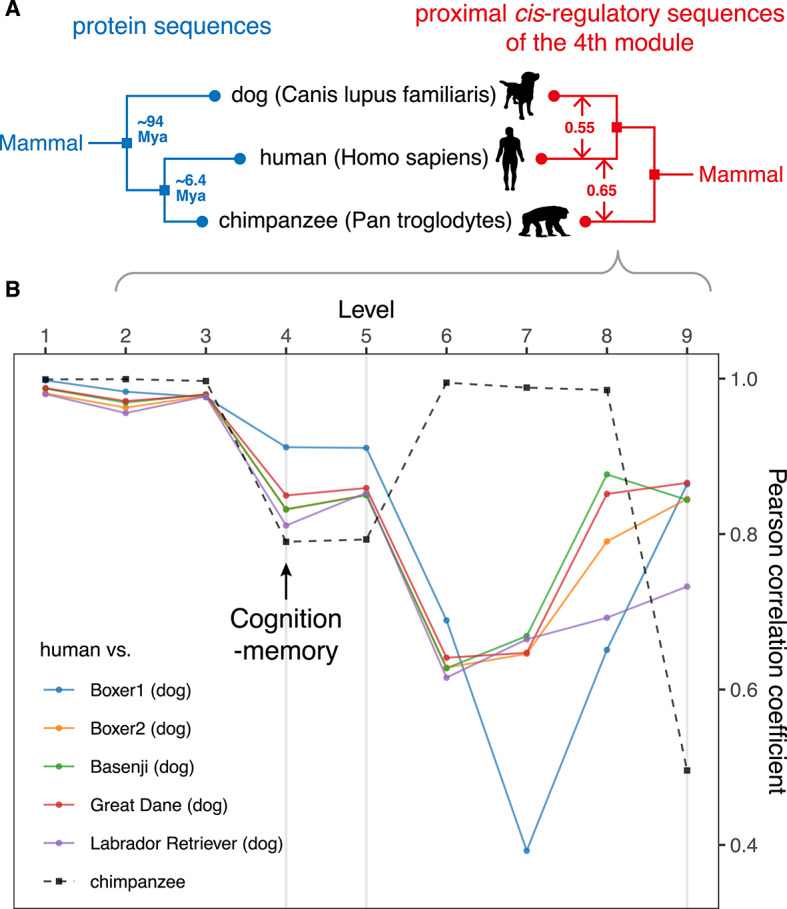
(**A**) **The cluster tree of humans, dogs, and chimpanzees based on protein sequences and the tree based on proximal regulatory sequences. **The left is based on protein sequences and the divergence times are marked on the tree. The right is based on the PCCs (Pearson correlation coefficients) and Euclidean distances between the motif-eigenvectors at the fourth CREF module. This module corresponds to cognition phenotypes and is obtained from proximal regulatory sequences. The Euclidean distances are marked on the tree. (**B**) **The line charts of PCCs between the top nine motif-eigenvectors of five dogs and chimpanzees versus those of humans. **The fourth, fifth, and ninth PCCs between humans and five dogs are greater than those between humans and chimpanzees.

Phylogenetic studies based on protein sequences demonstrated that compared to the dog, the chimpanzee is our closer evolutionary relative (Fig. 8A). Nevertheless, the evolution of the regulatory sequences could be a different story. As explained earlier, the representation of regulatory sequences should be different from that of protein sequences. The focus of this study is specifically on proximal *cis*-regulatory sequences. We use the CREF dual eigen-modules to represent the evolution of proximal *cis*-regulatory sequences. When comparing CREF dual eigen-modules between humans and apes, a human-specific cognition-memory eigen-module was found^13^. In the previous sections, we compared the CREF dual eigen-modules across five canids. Now we shift our attention to the eigen-modules of dogs, humans, and chimpanzees.

The comparison demonstrates that the fourth CREF dual eigen-module of humans is closer to dogs than to chimpanzees (Fig. 8A). First, we compare humans’ correlations with dogs and chimpanzees along motif-eigenvectors. Surprisingly, in terms of the fourth, fifth, and ninth motif-eigenvectors, all PCCs between humans and five dogs are greater than those between humans and chimpanzees (Fig. 8B). Second, we measure the distance between species by the Euclidean distances between their fourth motif-eigenvectors. The results show that the average Euclidean distances between humans and five dogs (0.55) are less than that between humans and chimpanzees (0.65, Fig. 8A). Third, we observe that long-term memory is up-regulated at the positive pole of the dog fourth gene-eigenvector (Fig. 3). Similarly, the fourth CREF eigen-module has also been found to be special to humans, as a group of biological pathways related to cognition, language, learning, and memory are enriched at the positive pole of the human fourth gene-eigenvector, but not along that of the chimpanzee^13^. Fourth, we identify a large number of binding elements of important transcription factors involved in the regulation of long-term memory around the positive poles of the fourth motif-eigenvector in both dogs and humans (Fig. 5).

We notice that dogs possess some special social-cognitive abilities that are not possessed by chimpanzees, such as understanding and communicating with human language, gestures, and facial expressions^5,6^. Our dual eigen-modules unveil a striking convergent evolution of dogs and humans in proximal regulatory sequences. This theory easily explains the social-cognitive abilities.

### Comparison of CREF dual eigen-modules between Canidae and Hominidae along motif-eigenvectors

Next, we compare CREF eigen-modules across species of different families, Canidae and Hominidae. In addition to the previously mentioned nine canids including five dogs, a dingo, a red fox, a dhole, and a wolf, we also include four hominids in our study: human, chimpanzee, orangutan, and gorilla. Based on their CREF matrices, we obtain their top pairs of gene- and motif-eigenvectors. To assess the similarity of the CREF eigen-module at a certain level among different species, we compute the pairwise PCCs of the corresponding motif-eigenvectors.

**Fig. 9:**
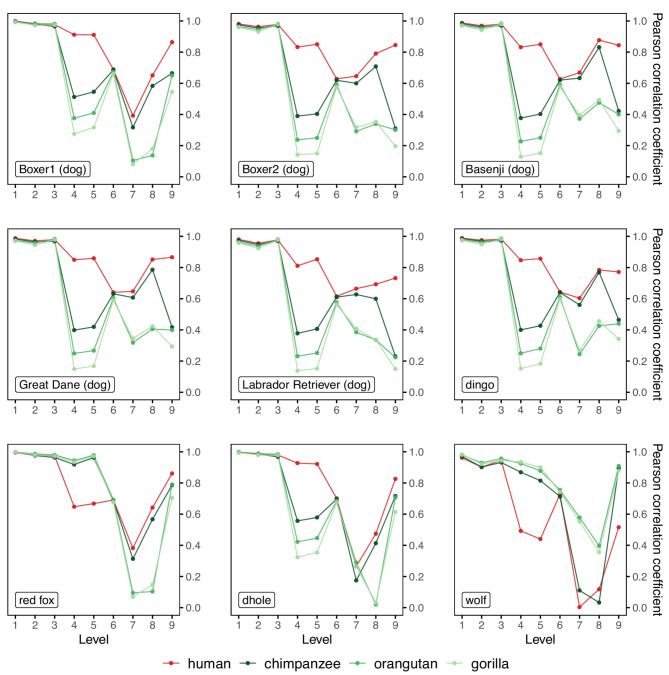
**The line charts of PCCs (Pearson correlation coefficients) between nine canids’ top nine motif-eigenvectors and those of four hominids. **For five dogs, dingoes, and dholes, their fourth, fifth, and ninth PCCs with humans are greater than those with three apes and decrease in the order of humans, chimpanzees, orangutans, and gorillas. Conversely, for red foxes and wolves, their fourth and fifth PCCs with humans are less than those with three apes. This suggests that dingoes, dholes, and dogs share a similar CREF pattern that is closer to humans than to the three apes; whereas red foxes and wolves display a similar CREF pattern that is closer to the three apes than to humans.

In each subplot of Fig. 9, one canid is taken as the reference, and its PCCs of motif-eigenvectors with each of the four hominids are shown from levels one to nine. Following our previous approach to hominids^13^, we first consider the top six PCCs. All top three PCCs are close to 1 except that wolves’ are slightly lower, indicating high conservation of these CREF eigen-modules. The sixth PCCs are within the narrow range of 0.6 to 0.7 across four hominids in all subplots. In contrast, the fourth and fifth PCCs vary among hominids. In the cases of five dogs together with dingoes and dholes, from high to low are humans, chimpanzees, orangutans, and gorillas; in the cases of red foxes and wolves, humans’ are less than three apes’.

Interestingly, the ninth level shows a similar CREF pattern as observed in the fourth and fifth levels. In the cases of five dogs, dingoes, and dholes, their ninth PCCs with hominids are in the descending order of humans, chimpanzees, orangutans, and gorillas.

Taking the results of correlation at the top nine levels together, we can draw the following two conclusions. First, dingoes and dholes both show high similarity to dogs, while red foxes and wolves share a similar CREF pattern at levels four, five, and nine. Second, dogs, dingoes, and dholes are closer to humans than to the three apes, whereas red foxes and wolves are closer to the three apes than to humans. These results indicate that the tens of thousands of years of domestication facilitated a convergent evolution in the CREF eigen-modules of humans and dogs.

### CREF—a formulation of *cis*-regulatory element evolution able to account for phenotypical saltation

At the phenotypical level, Darwin primarily held the view that most evolutionary changes followed a gradual process, although he did not deny the occurrences of jumps. In related species such as humans and apes, dogs and wolves, phenotypical saltation does exist without any doubt. At the molecular level, King and Wilson proposed that the major biological differences between humans and chimpanzees might be accounted for by mutations in regulatory sequences^10^. Highly anticipated is a mathematical formulation that can represent the *cis*-regulatory sequences so that their evolution can explain the phenotypical saltation.

In this study, we apply CREF, a mathematical and biologically meaningful representation of *cis*-regulatory sequences, to the understanding of the phenotypical saltation in Canidae, and the convergent evolution of dogs and humans. Our results demonstrated that other than protein sequences, gene regulation plays a key role in a species’ traits, likely through development. The model of regulatory sequences is different from that of protein sequences. Not only the presence of *cis*-elements but also their frequencies are crucial for deciphering the regulatory saltation. Although gradual evolution might be the only mode in the protein sequence, the evolution of gene regulation exhibits both gradual and saltational modes.

## Discussion

The analysis of the *cis*-regulatory sequences by CREF modules not only sheds insights into Canidae evolution, but also explains why dogs, but not chimpanzees, share more similar social-cognitive abilities with humans. We integrate the proximal *cis*-regulatory sequences of all protein genes so that the saltation can be formulated as a phase transition in statistical physics. It is the saltation of gene regulation that accounts for, to a great extent, the phenotypic saltation.

The CREF analysis relies on the proximal regulatory sequences of all or most protein-coding genes, rather than the entire genome. In the case of Hominidae species, we tested various versions of genomes and annotations and found that, while the degree of rotation varied to some extent, the saltation was consistently observed^13,15^. The Canidae and Hominidae genomes used in this study are summarized in Table S13, including six dog genomes and two dingo genomes. Comparative CREF analysis showed that although the degree of rotation varied slightly across versions, the saltation at the fourth level was consistently observed in the dogs and dingoes. Therefore, their results are considered reliable. On the other hand, only one genome is currently available for each of the wolf, dhole, and red fox, and their results require further verification in future studies.

To make robust inferences, we utilized the following techniques. First, we applied a robust version of singular value decomposition to help mitigate the potential influence of various sources of noise, including errors in the DNA sequences and annotations, as well as uncertainty in the motif position weight matrices (Robust SVD of CREF matrix, Materials and Methods). Second, in the enrichment analysis, we adopted the robust Wilcoxon scoring method (Supplementary Text, subsection "Enrichment analysis by the Wilcoxon rank sum scoring method"). This rank-based statistical method allowed us to evaluate the significance of the gene subsets more robustly.

The abundance of myelin in the dog brain was verified at the proteomic level in our previous study^9^. By quantifying the relative levels of three major myelin proteins with reference to the cerebellum, we reported that the total amount of myelin protein in the hippocampus of dogs reaches approximately one-third of humans while this ratio even reaches two-thirds in the prefrontal cortex, much higher than those of mice. The significant enhancement of myelination in the dog fourth eigen-module explains the myelin abundance in the dog brain. To our knowledge, no other theory can explain the myelin experimental results so far.

The saltation in CREF modules occurred at the systems biology level as a result of the emergence of a 2-D eigen-direction from cumulative changes in regulatory sequences. In addition to point mutations, the source of variations may also involve mutations related to transposable elements. In our previous study, mutations related to Alu elements have been demonstrated to be one of the important driving forces for the genomic saltation in Hominidae through the notion of motifs present on Alu elements^13,14^. In this study, we observed a significant increase in the number of motifs present on Can-SINE elements among the top motifs of the fourth polarized motif-eigenvectors of dog, dingo, and dhole and the fifth ones of red fox and wolf (Fig. 7A). This result suggests that mutations related to Can-SINE elements are likely to play a role in the Canidae genomic saltation.

However, the specific genetic mechanism by which the Can-SINE elements, along with other factors, influence the changes of the CREF eigen-modules and ultimately lead to saltation is mostly unknown. Because of the definition of CREF matrices, it is reasonable to hypothesize that the Can-SINE elements exert their influence by altering the distribution of *cis*-regulatory elements around the gene transcription start site (TSS). Based on the cases in Hominidae, we propose three possible mechanisms. First, some dog-specific Can-SINE elements are inserted directly into the proximal regulatory regions. Second, one or more Can-SINE elements are present around the TSS of a dog-specific transcript. Finally, the presence of Can-SINE elements causes a shift of TSS, leading to changes in the *cis*-elements in the proximal regulatory regions.

Our theory presents a mathematical framework that provides an explanation for the phenotypic jumps in evolution. Different from gradualism, there are other hypotheses regarding sudden and rapid changes in traits, such as saltationism and punctuated equilibrium. However, most of the evidence supporting these hypotheses is derived from the fossil record. In contrast, our comparative study is based on the genomes of existing species. Surprisingly, we discover a degenerate point in the regulatory regions of these existing genomes. When a species crosses a degenerate point where two neighboring eigenvalues approach each other, their corresponding eigenvectors become increasingly sensitive to perturbations, rendering them more prone to rotation or mutation. This critical sensitivity ultimately gives rise to the occurrence of phenotypic jumps in evolution.

Note that we have previously reported the existence of a degenerate point in the evolutionary history of Hominidae^13^. However, it remains unknown whether a species at this degenerate point actually existed in history. In this article, we found that the red fox is a species just near the degenerate point of the saltation in the evolution of Canidae. Based on this discovery, certain experiments can be designed to figure out the most influential factors in the saltation.

The current study provides a new perspective on the mechanism of speciation. In principle, speciation corresponds to the genetic mutations and fixation of alleles with high fitness. There exist various forms of genetic mutations in the genome, including mutations in protein sequences and mutations in regulatory sequences. In this article, the proposed mathematical model represents the evolution of regulatory sequences differently from the existing ones of point mutations. The new model uncovers a type of polymorphism in the regulatory structure. Near the degenerate point, mutations in the regulatory structure become critically more influential and lead to rapid and dramatic phenotypic changes, primarily through the regulation of development. Such phenotypic changes may have significant survival advantages in some circumstances, allowing them to be selected and dominate within a short period of time, and eventually lead to the formation of a new species. This regulatory sequence evolution of uneven paces stands in contrast to the relatively slow and uniform process of point mutations in the protein sequences.

Moreover, the varying mutation rate could potentially be another underlying factor for phenotypic jumps. The genetic driving force that leads to the saltation, other than point mutations, probably includes mutations related to transposable elements such as Can-SINE elements. The mutation rate of Can-SINE elements in a species varied in history^32^, possibly much larger in certain periods. This is true at least in the case of Alu elements in the Hominidae evolution^33,34^. A recent report provided more examples of human-specific Alu and SVA insertions into proximal regulatory sequences^15^, along with information about their chromosomal locations.

## Materials and methods

### *Cis*-regulatory element frequency profile

Rather than relying on sequence alignment, our mathematical framework characterizes the proximal regulatory sequence of a gene by the frequencies of *cis*-regulatory elements, which are then compared across species. Based on each of the five canid genomes, we constructed a species-specific *cis*-regulatory element frequency matrix, referred to as the CREF matrix, according to the following steps.

First, the proximal regulatory region of each protein-coding gene was extracted from the chromosome or scaffold according to the position of its transcription start site (TSS) in gene annotation. In this study, the proximal regulatory region of a protein-coding gene is defined to be the region between − 1000 and + 500 bp of the gene TSS. Note that we focused our analysis on protein-coding genes. When a gene has multiple annotated transcripts, the start position of its most upstream transcript toward the 5’ end is chosen as the TSS.

The genome and gene annotations we used for the dog, dingo, and red fox were CanFam3.1, ASM325472v1, and VulVul2.2, respectively, downloaded from the Ensembl database (release 102)^35^. For the dhole and wolf, the genome and gene annotation came from the iDog database^36^ due to the lack of available genomes in the Ensembl database up to now. The information regarding other genomes that are used in this study can be found in Table S13. The sequence of the proximal regulatory region was extracted using SeqKit^37^.

Second, motif frequencies in the proximal regulatory region of all genes were computed using the MATCH program with the minFN (minimize false negative rate) option^38^. A total of 1403 vertebrate motifs together with their position weight matrices (PWMs) from the TRANSFAC database were used in this study^19^.

Finally, the collection of all motif frequencies of all genes was arranged in a CREF matrix*.* The CREF matrix $\widetilde{C}$ of a species is a matrix of $g$ rows and $m$ columns, where $g$ and $m$ denote the total number of protein-coding genes and the total number of motifs, respectively. In the matrix $\widetilde{C}$, each row corresponds to a gene and each column corresponds to a motif. The entry in the $i$-th row and $j$-th column of the matrix $\widetilde{\text{C}}$ is the frequency of $j$-th motif in the proximal regulatory region of $i$-th gene.

### Robust SVD of CREF matrix

The CREF matrix serves as the starting point for the dual eigen-analysis. We decomposed each CREF matrix into dual eigen-modules via singular value decomposition (SVD). Then we aligned and compared the resulting dual eigen-modules across species. To mitigate outliers’ impact arising from various sources, we employ a robust version of SVD in practice.

The SVD of the species-specific CREF matrix is the first step of the dual eigen-analysis. A CREF matrix, denoted by $\widetilde{C}$, might be contaminated by outliers caused by various uncontrollable factors such as the quality of the genome, the gene annotation, and motifs’ PWMs. Therefore, we express the matrix $\widetilde{C}$ as the sum of two components, a low-rank matrix $C$ expected to capture the principal genomic information of the CREF profile, and a sparse matrix $S$ containing other unexpected variations.

Although the classical SVD is able to recover the low-rank component $C$ of the CREF matrix $\widetilde{C}$ when the noise is additive and is independently and identically distributed as Gaussian, it is not robust when a substantial portion of the elements are subject to unknown and possibly extreme disturbances. Therefore, we adopt a robust version of the SVD. The low-rank component $C$ is recovered from $\widetilde{C}$ by solving the following convex optimization problem:

$$\underset{C,S}{\text{min}}{\parallel C\parallel }_{*}+\lambda {\parallel S\parallel }_{1} \, \text{  subject to  } \widetilde{C}=C+S,$$

where ${\parallel C\parallel }_{*}$ denotes the nuclear norm of matrix $C$, i.e., the sum of its singular values, ${\parallel S\parallel }_{1}$ denotes the ${l}_{1}$-norm of the matrix $S$ seen as a long vector in ${\mathbb{R}}^{g\times m}$, i.e., the total sum of the absolute values of matrix $S$ entries, and the tuning parameter $\lambda$ is chosen to be $\frac{1}{\sqrt{g}}$ as in the paper^20^. To solve this convex optimization problem, we employ the inexact augmented Lagrange multipliers (IALM) algorithm^39^.

After that, we applied the classical SVD to matrix $C$:

$$C={U}_{g\times r}{\Sigma }_{r\times r}{V}_{r\times m}^{T}={\sum }_{k=1}^{r}{\rho }_{k}{u}_{k}{v}_{k}^{T},$$

where $r$ denotes the rank of matrix $C$, the column vectors of matrices $U$ and $V$ are respectively ${\left\{{u}_{k}\right\}}_{k=1}^{r}$ and ${\left\{{v}_{k}\right\}}_{k=1}^{r}$, the elements of the diagonal matrix $\Sigma$ are singular values ${\left\{{\rho }_{k}\right\}}_{k=1}^{r}$ in descending order. ${\left\{{u}_{k}\right\}}_{k=1}^{r}$ are mutually orthogonal and so are ${\left\{{v}_{k}\right\}}_{k=1}^{r}$. We refer to ${\left\{{u}_{k}\right\}}_{k=1}^{r}$ and ${\left\{{v}_{k}\right\}}_{k=1}^{r}$ as gene-eigenvectors and motif-eigenvectors, respectively. These two sets of vectors are of great importance and are the basis of CREF dual-eigen analysis.

We make two notes here. First, the result of SVD is not unique, since the sign of the product ${u}_{k}{v}_{k}^{T}$ does not change if the signs of ${u}_{k}$ and ${v}_{k}$ change simultaneously. Therefore, to ensure the consistency of the comparison across different canids, we set the signs of their vectors ${\left\{{u}_{k}\right\}}_{k=1}^{r}$ and ${\left\{{v}_{k}\right\}}_{k=1}^{r}$ as follows. Taking dogs as the reference, for the other four canids, we determine the sign of ${v}_{k}$ so that its inner product with dogs’ ${v}_{k}$ is positive. Accordingly, the sign of each ${u}_{k}$ is determined by the sign of the corresponding ${v}_{k}$.

Second, the original top singular value and eigenvectors of a CREF matrix are essentially the adjusted averages. These baselines offer little interesting biological explanations for the *cis–trans* regulatory profiles. Therefore, when we refer to the singular values or eigenvectors, we exclude the baseline eigen-component throughout the article.

The entries in the gene-eigenvector are referred to as gene loadings; we sort the loadings in the gene-eigenvector ${u}_{k}$ in the descending order and obtain the polarized gene-eigenvector, denoted by ${\overrightarrow{u}}_{k}$. Similarly, the entries in the motif-eigenvector are referred to as motif loadings; we sort the loadings in the motif-eigenvector ${v}_{k}$ in the descending order and obtain the polarized motif-eigenvector, denoted by ${\overrightarrow{v}}_{k}$. Each pair of polarized gene and motif-eigenvectors, together with the singular value comprise a dual eigen-module of CREF, referred to as a CREF dual eigen-module. Their detailed definition can be found in the Supplementary Text, subsection "Mathematical definitions of key terms".

### Stability of 2-D eigenspace

The saltation in the CREF matrix is rooted in the sensitivity of its eigenvalues and eigenvectors, a phenomenon explained by perturbation theory. As two eigenvalues approach each other, their corresponding eigenvectors become unstable. However, the two-dimensional eigenspace spanned by these vectors remains relatively stable. Compared to single-nucleotide polymorphisms, it is this 2-D eigenspace that constitutes the regulatory variation or polymorphism—in a systems biology sense—subject to selection.

Based on the perturbation theory of matrix, we formulate the stability of 2-D eigenspace. Without loss of generality, we consider the motif-eigenvectors ${\left\{{v}_{k}\right\}}_{k=1}^{r}$, which are the eigenvectors of the symmetric matrix $D={C}^{T}C={\sum }_{k=1}^{r}{\rho }_{k}^{2}{v}_{k}{v}_{k}^{T}={\sum }_{k=1}^{r}{\lambda }_{k}{v}_{k}{v}_{k}^{T},$ where ${\lambda }_{k}={\rho }_{k}^{2}$. Let $\overline{D }=D+E$, where $E$ is a symmetric perturbation. We aim to quantify the influence of the perturbation $E$ on the 2-D eigenspace spanned by the fourth and fifth motif-eigenvectors.

Assuming that eigenvalues ${\lambda }_{4}$ and ${\lambda }_{5}$ are well separated from other eigenvalues and the symmetric perturbation $E$ is sufficiently small, we have ${\lambda }_{1}\ge {\lambda }_{2}\ge {\lambda }_{3}>{\lambda }_{4}\ge {\lambda }_{5}>{\lambda }_{6}\ge \cdots \ge {\lambda }_{m}$ and the perturbed eigenvalues ${\overline{\lambda }}_{1}\ge {\overline{\lambda }}_{2}\ge {\overline{\lambda }}_{3}>{\overline{\lambda }}_{4}\ge {\overline{\lambda }}_{5}>{\overline{\lambda }}_{6}\ge \cdots \ge {\overline{\lambda }}_{m}$. We employ the following definition to measure the distance between two subspaces $\mathcal{X}$ and $\mathcal{Y}$:

$$\rho_{2} \left( {{\mathcal{X}},{\mathcal{Y}}} \right) = {\text{max}}\left\{ {\begin{array}{*{20}c} {\mathop {{\text{max}}}\limits_{{\begin{array}{*{20}c} {x \in {\mathcal{X}}} \\ {\left\| {x} \right\|_{2} = 1} \\ \end{array} }} \mathop {\min }\limits_{{y \in {\mathcal{Y}}}} \left\| {x - y} \right\|_{2} ,} & {\mathop {{\text{max}}}\limits_{{\begin{array}{*{20}c} {y \in {\mathcal{Y}}} \\ {\left\| {y} \right\|_{2} = 1} \\ \end{array} }} \mathop {\min }\limits_{{x \in {\mathcal{X}}}} \left\| {x - y} \right\|_{2} } \\ \end{array} } \right\} = \left\| {X_{ \bot }^{T} Y} \right\|_{2} ,$$

where ${\Vert \cdot \Vert }_{2}$ denotes the 2-norm of a vector or a matrix. The columns of matrices ${X}_{\perp }$ and $Y$ respectively form the orthonormal bases of the orthogonal complement ${\mathcal{X}}_{\perp }$ of $\mathcal{X}$ and the orthonormal bases of $\mathcal{Y}$.

Under specific conditions, the distance between the 2-D eigenspaces spanned by original eigenvectors ${v}_{4}$ and ${v}_{5}$ and the 2-D eigenspaces spanned by perturbed eigenvectors ${\overline{v} }_{4}$ and ${\overline{v} }_{5}$ has the following upper bound^40^:

$$\begin{aligned} \rho_{2} \left( {{\text{span}}\{ v_{4} ,v_{5} \} ,{\text{span}}\left\{ {\overline{v}_{4} ,\overline{v}_{5} } \right\}} \right) & \le \frac{{2\left\| E \right\|_{2} }}{{\mathop {\min }\limits_{i = 4,5,j \ne 4,5} \left| {\lambda_{i} - \lambda_{j} } \right| - \left\| E \right\|_{2} }} \\ & = \frac{{2\left\| E \right\|_{2} }}{{{\text{min}}\{ \lambda_{3} - \lambda_{4} ,\;\lambda_{5} - \lambda_{6} \} - \left\| E \right\|_{2} }}. \\ \end{aligned}$$

The upper bound of the perturbed deviation depends on both the magnitude of the disturbance and the distance between the eigenvalues of the original matrix. Therefore, a larger distance between the fourth/fifth eigenvalues and the rest eigenvalues will result in a smaller perturbed deviation, thereby, higher stability of 2-D eigenspace.

### Polarization degree of eigenvectors

The pivotal transition from classical SVD to dual eigen-analysis is the polarization of motif- and gene-eigenvectors. Each dual eigen-module can then be biologically interpreted through the polarized eigenvectors. By the definition of the CREF matrix, the *cis*-elements at the poles of the motif-eigenvector are capable of regulating the genes located near the poles of the gene-eigenvector. On the other hand, the squares of either motif or gene loadings sum to one, and most loadings are close to zero due to the large number of motifs and genes. Therefore, it is helpful to compare the degree of polarization across CREF eigen-modules.

In this section we define a metric, polarization degree, to quantitatively measure the concentration of the gene or motif loadings near the two poles of an eigenvector. The polarized eigenvector can be a polarized gene-eigenvector or a polarized motif-eigenvector.

In the definition of polarization degree, the importance of a gene or a motif in a CREF eigen-module is measured by its square, which is referred to as its energy. All energies sum to one according to the eigenvector definition.

We give the mathematical definition of polarization degree in the Supplementary Text, subsection "Polarization degree of eigenvectors". The following example helps to understand the definition intuitively. Let $\overrightarrow{w}$ be a polarized gene- or motif-eigenvector. Suppose its polarization degree at the positive pole is ${d}^{+}\left(\overrightarrow{w}\right)=0.9$, then ${1-d}^{+}\left(\overrightarrow{w}\right)=10\%$ genes or motifs at the positive end of $\overrightarrow{w}$ contain at least ${d}^{+}\left(\overrightarrow{w}\right)=90\%$ of the total energy of all genes or motifs at the positive end of $\overrightarrow{w}$. Therefore, the polarization degree reasonably measures the concentration of energy at the two poles of an eigenvector. A large polarization degree indicates a higher concentration of energy at this end, and vice versa.

## Supplementary Information

Supplementary Information.

## Data Availability

All data generated or analyzed during this study are included in this article and its supplementary materials files. The source codes and data used for the study are available at https://github.com/JianhuiShi/Canidae-CREF-analysis.

## Abbreviations

CREF: *Cis*-Regulatory element frequency
TSS: Transcription start sites
SVD: Singular value decomposition
PCC: Pearson correlation coefficient
LTP: Long-term potentiation
LTD: Long-term depression
CNS: Central nervous system
PNS: Peripheral nervous system
SINEs: Short interspersed nuclear elements
Can-SINEs: Carnivore-specific SINEs
MPCS: Motifs present on Can-SINE elements
minFP: Minimize false positive rate
minSUM: Minimize the sum of both errors
minFN: Minimize false negative rate
PWM: Position weight matrix
