# Supplementary material for: Saltations of cis-regulatory modules in Canidae and Hominidae
Source: Sci Rep. 2025 Aug 6;15:28838. doi: 10.1038/s41598-025-13034-y (PMC12328751; doi:10.1038/s41598-025-13034-y)
Supplement: Supplementary file 1 — Supplementary Information. [file 41598_2025_13034_MOESM1_ESM.pdf]

Supplementary Materials

Saltations of *Cis*-regulatory Modules in Canidae and Hominidae

Jianhui Shi *et al.*

*Corresponding author. Email: lilei@amss.ac.cn

This PDF file includes:

Supplementary Text
References 1-56
Figs. S1 to S17
Tables S1 to S14

Contents

Supplementary Text ... 4
Mathematical definitions of key terms ... 4
Motivations behind the definition of *cis*-regulatory element frequency ... 5
PCC thresholds of conservation when comparing motif-eigenvectors ... 5
Comparison along motif-eigenvectors including multiple breeds of dogs ... 5
Rotations in the 2-D eigenspaces including multiple breeds of dogs ... 6
Sensitivity of eigenvalues and eigenvectors ... 6
Stability analysis of the top singular values by random sampling ... 7
Stability analysis of CREF eigen-modules by random shuffling ... 7
Enrichment analysis by the Wilcoxon rank sum scoring method ... 9
Details of the long-term memory-related pathways significantly enriched at the positive pole of the fourth gene-eigenvector ... 10
Details of the myelination-related pathways significantly enriched at the poles of the fourth gene-eigenvector ... 11
Details of the cochlea development-related pathways significantly enriched at the poles of the fourth gene-eigenvector ... 12
Details of the long-term memory regulators standing around the positive pole of the fourth motif-eigenvector ... 12
Details of the myelination regulators standing around the poles of the fourth motif-eigenvector ... 13
Details of the cochlea development regulators standing around the poles of the fourth motif-eigenvector ... 14
Evidence supporting the hypothesis of the saltation near the degenerate point ... 15
Motivations for studying Can-SINE elements ... 15
Frequency comparison of MPCS and other motifs at the poles of the fourth and fifth motif-eigenvectors ... 16
MPCSs at the top of polarized motif-eigenvectors with the MATCH program option set to minFP ... 17
Polarization degree of eigenvectors ... 17
References ... 19
Fig. S1. ... 26
Fig. S2. ... 27
Fig. S3. ... 28
Fig. S4. ... 29
Fig. S5. ... 30
Fig. S6. ... 31
Fig. S7. ... 32
Fig. S8. ... 33
Fig. S9. ... 34
Fig. S10. ... 35
Fig. S11. ... 36
Fig. S12. ... 37
Fig. S13. ... 38
Fig. S14. ... 39
Fig. S15. ... 40

Fig. S16. 41
Fig. S17. 42
Table S1. 43
Table S1 (continued). 44
Table S1 (continued). 45
Table S1 (continued). 46
Table S2. 47
Table S3. 48
Table S3 (continued). 49
Table S4. 50
Table S4 (continued). 51
Table S5. 52
Table S6. 53
Table S7. 54
Table S8. 55
Table S9. 56
Table S10. 57
Table S11. 58
Table S12. 59
Table S13. 60
Table S14. 61
Table S14 (continued). 62

Supplementary Text

Mathematical definitions of key terms

First, based on each of the five canid genomes, we constructed a species-specific CREF matrix.

CREF matrix (*cis*-regulatory element frequency matrix) The CREF matrix $\tilde{C}$ of a species is a matrix of g rows and m columns, where g and m denote the total number of protein-coding genes and the total number of motifs, respectively. In the matrix $\tilde{C}$, each row corresponds to a gene and each column corresponds to a motif. The entry in the i-th row and j-th column of the matrix $\tilde{C}$ is the frequency of j-th motif in the proximal regulatory region of i-th gene.

Second, we applied the robust singular value decomposition (SVD) on the CREF matrix $\tilde{C}$ as follows:

$$\min_{C,S} \| C \|_* + \lambda \| S \|_1 \quad \text{subject to} \quad \tilde{C} = C + S,$$

$$C = U_{g\times r}\Sigma_{r\times r}V_{r\times m}^T,$$

where r denotes the rank of matrix C.

Gene-eigenvector The gene-eigenvectors $\{u_k\}_{k=1}^r$ are column vectors of the matrix U, i.e., left singular vectors of the low-rank matrix C.

Motif-eigenvector The motif-eigenvectors $\{v_k\}_{k=1}^r$ are column vectors of the matrix V, i.e., right singular vectors of the low-rank matrix C.

Gene loading Let the gene-eigenvector $u_k = \left(u_{1,k}, u_{2,k}, \dots, u_{g,k}\right)^T$, then the loading of *i*-th gene at the *k*-th level is its weight $u_{i,k}$ in the gene-eigenvector u_k.

Motif loading Let the motif-eigenvector $v_k = \left(v_{1,k}, v_{2,k}, \dots, v_{m,k}\right)^T$, then the loading of *j*-th motif at the *k*-th level is its weight $v_{j,k}$ in the motif-eigenvector v_k.

Third, we polarized the gene- and motif-eigenvectors by sorting their loadings.

Polarized gene-eigenvector Let the gene-eigenvector $u_k = \left(u_{1,k}, u_{2,k}, \dots, u_{g,k}\right)^T$, then the polarized gene-eigenvector $\vec{u}_k = \left(u_{\tau(1),k}, u_{\tau(2),k}, \dots, u_{\tau(g),k}\right)^T$, where τ is a permutation of the set $\{1,2,\dots,g\}$ satisfying $u_{\tau(1),k} \geq u_{\tau(2),k} \geq \cdots \geq u_{\tau(g),k}$.

Polarized motif-eigenvector Let the motif-eigenvector $v_k = \left(v_{1,k}, v_{2,k}, \dots, v_{m,k}\right)^T$, then the polarized motif-eigenvector $\vec{v}_k = \left(v_{\pi(1),k}, v_{\pi(2),k}, \dots, v_{\pi(m),k}\right)^T$, where π is a permutation of the set $\{1,2,\dots,m\}$ satisfying $v_{\pi(1),k} \geq v_{\pi(2),k} \geq \cdots \geq v_{\pi(m),k}$.

CREF dual eigen-module The *k*-th CREF dual eigen-module $C_k = (\rho_k, \vec{u}_k, \vec{v}_k)$ is the triple of the singular value ρ_k, polarized gene-eigenvector $\vec{u}_k$, and polarized motif-eigenvector $\vec{v}_k$ at the *k*-th level.

Motivations behind the definition of *cis*-regulatory element frequency

The *cis*-regulatory elements frequency (CREF) is defined to be the occurrences of the *cis*-elements that can bind to a specific transcription factor in the proximal regulatory region of a protein-coding gene [1]. There are several motivations behind this definition. First, the experimental study in the context of development revealed that, within a certain range, there exists a roughly proportional relationship between the number of mRNA molecules in the vicinity of the transcription site and the number of bound transcription factors [2]. Since the maximum of the latter number is bounded by the *cis*-element frequency, this frequency provides a measure of the capacity of the transcriptional regulation. Second, a recent single-cell study further demonstrated that transcription factor binding quantities linearly contribute to promoter activation [3], and in turn, control the gene expressions. Third, Feng et al. demonstrated that the chance of at least one successful binding event is roughly proportional to the *cis*-elements frequency, thereby defining the *cis*-*trans* binding strength by the number of *cis*-elements [4]. This binding strength turned out to be effective in the inference of transcriptional regulation [5].

PCC thresholds of conservation when comparing motif-eigenvectors

When comparing CREF modules along motif-eigenvectors, we set up thresholds to determine whether CREF eigen-modules are highly conserved or not. To do this, we select five dogs (four breeds including two Boxers, a Basenji, a Great Dane, and a Labrador Retriever) from the Ensembl database [6]. Using genomes from the same source excludes the variations due to different databases. We obtain their CREF eigen-modules and compute the PCC between each pair of them at each level. Such ten PCCs at a certain level are used to construct the empirical 95 percent confidence interval of the PCC. Finally, we take the confidence lower limit as the threshold of conservation (Fig. S2). If the PCC between motif-eigenvectors of two species is beyond this threshold, the module difference between species is considered to be smaller than that within species. In such cases, we define the module at this level to be highly conserved; otherwise, it is defined to be divergent. Roughly speaking, as the level increases, the threshold of conservation shows a decreasing trend. In other words, the requirement for conservation drops down.

Comparison along motif-eigenvectors including multiple breeds of dogs

To enhance the reliability of comparison along motif-eigenvectors, we further include different breeds of dogs, two dingoes, and one dhole in our study. Among them, six dogs of five breeds include two Boxers, a Basenji, a Great Dane, a Labrador Retriever, and a German Shepherd.

On the one hand, all PCCs between each pair of top six motif-eigenvectors of dogs, dingoes, and dholes are beyond 0.9 (Fig. S3A). Moreover, their range is similar to the range of PCCs between each pair of six dogs (Fig. S3B). These observations indicate that dogs, dingoes, and dholes are highly correlated at the top six levels. On the other hand, red foxes and wolves both exhibit weak

correlations with all breeds of dogs, dingoes, and dholes at the fourth and fifth levels, demonstrating a divergence at these two levels (Fig. S4 and Fig. S5).

Rotations in the 2-D eigenspaces including multiple breeds of dogs

We project the fourth and fifth motif-eigenvectors of four breeds of dogs onto the 2-D eigenspaces of wolves. The result shows that the two eigen-directions of all four breeds of dogs rotate about 82° to 88° from those of wolves (Fig. S6).

We also project the fourth and fifth motif-eigenvectors of red foxes onto the 2-D eigenspaces of the other nine canids including five breeds of dogs, two dingoes, a dhole, and a wolf. The result shows that the two eigen-directions of red foxes rotate at a relatively large angle (between 62° and 75°) from all nine canids with the exception of wolves (Fig. S7).

Sensitivity of eigenvalues and eigenvectors

In this section, we illustrate the sensitivity of CREF eigen-modules based on the perturbation theory of matrix. The matrix perturbation theory provides valuable insights into the saltation near the degenerate point and the rotation of the fourth and fifth eigen-directions.

First, we state some relevant mathematical facts. The singular values of CREF matrix C are the square roots of the nonzero eigenvalues of the symmetric matrix CC^T and C^TC. The gene-eigenvectors of C are the eigenvectors of CC^T; and the motif-eigenvectors of C are the eigenvectors of C^TC. This can be seen from the eigen-decomposition:

$$CC^T = U(\Sigma\Sigma^T)U^T = \sum_{k=1}^{r} \rho_k^2 u_k u_k^T = \sum_{k=1}^{m} \lambda_k u_k u_k^T ,$$

$$C^TC = V(\Sigma^T\Sigma)V^T = \sum_{k=1}^{r} \rho_k^2 v_k v_k^T = \sum_{k=1}^{m} \lambda_k v_k v_k^T ,$$

where $\lambda_1 \geq \lambda_2 \geq \cdots \geq \lambda_m$ are the eigenvalues of CC^T and C^TC, and $\lambda_k = \rho_k^2$.

Below we analyze the perturbation on C^TC. We introduce the following notations. Assume that E is a symmetric perturbation of C^TC. $\bar{C}^T\bar{C}$ is the matrix after perturbation, i.e., $\bar{C}^T\bar{C} = C^TC + E$. $\bar{\lambda}_1 \geq \bar{\lambda}_2 \geq \cdots \geq \bar{\lambda}_m$ are the eigenvalues of $\bar{C}^T\bar{C}$. $\bar{v}_k$ is the corresponding eigenvector of $\bar{\lambda}_k$. Under specific conditions, the perturbed eigenvalues and eigenvectors of the fourth and fifth eigen-modules have the following expansions [7]:

$$\begin{cases} \bar{\lambda}_4 = \lambda_4 + v_4^T E v_4 + O(\|E\|_2^2) \\ \bar{\lambda}_5 = \lambda_5 + v_5^T E v_5 + O(\|E\|_2^2) \end{cases},$$

$$\begin{cases} \bar{v}_4 = v_4 + \dfrac{v_5^T E v_4}{\lambda_4 - \lambda_5} v_5 + \sum_{k\neq 4,5} \dfrac{v_k^T E v_4}{\lambda_4 - \lambda_k} v_k + O(\|E\|_2^2) \\ \bar{v}_5 = v_5 - \dfrac{v_4^T E v_5}{\lambda_4 - \lambda_5} v_4 + \sum_{k\neq 4,5} \dfrac{v_k^T E v_5}{\lambda_5 - \lambda_k} v_k + O(\|E\|_2^2) \end{cases},$$

where $\|\cdot\|_2$ denotes the 2-norm of a matrix, and the big O notation indicates the asymptotic upper bound as in mathematical analysis. The expansions indicate that the interference of one eigenvector to another is roughly inversely proportional to the distance between the two eigenvalues. After the normalization of the vectors, the perturbation approximately generates a rotation between v_4 and v_5.

In our case, if a species is near the degenerate point, its eigenvalues λ_4 and λ_5 are extremely close but well separated from other eigenvalues. The interaction between their eigenvectors will become dominant while those between other pairs are ignorable. This makes the CREF eigen-modules highly sensitive to even small perturbations in v_4 and v_5. As a result, even slight genetic variations within the regulatory regions can lead to a significant divergence in the eigen-directions.

Stability analysis of the top singular values by random sampling

We apply a re-sampling method without replacement [1] so that the sampling distributions of singular values tell us the stability of CREF eigen-modules concerning motif selection. The re-sampling process consists of three steps: (i) randomly select 80% motifs from 1403 motifs without replacement; extract the corresponding columns of these motifs from the original CREF matrix C to form a perturbed matrix denoted by C^i; (ii) calculate the SVD of C^i and collect its singular values ρ_k^i; (iii) repeat the above two steps 1000 times. The sampling distribution of each singular value is obtained based on $\{\rho_k^i\}_{i=1}^{1000}$.

According to the mathematical perturbation theory, the stability of CREF eigen-modules relies on the relative distances between adjacent singular values (section "Sensitivity of eigenvalues and eigenvectors", Supplementary Text). Accordingly, we consider the overlap between the distributions of adjacent singular values (Fig. S8). A large overlap indicates a small relative distance and unstable modules. In the cases of dogs (Boxer), red foxes, and dholes, the sampling distributions of the top three and the sixth singular values are well separated from their adjacent ones. However, the fourth and fifth ones exhibit some overlap. Red foxes, dholes, and dogs are in the descending order of the overlapping proportion between the fourth and fifth singular value distributions indicated by their 95% confidence interval. This ranking is in line with the inverse order of their relative distances between the fourth and fifth levels (1.9%, 6.3%, and 9.7%). The largest overlapping proportion of the red fox among the five canids indicates that the red fox is closest to the degenerate point that characterizes the onset of saltation.

Furthermore, the distribution of the seventh and eighth singular values overlap by a large portion in all five canids. Between the eighth and ninth ones, the overlap is also observed in dogs and dingoes. This simulation result supports the previous conclusion that the fourth, fifth, seventh, eighth, and ninth CREF eigen-modules are not as stable as the modules at other levels.

Stability analysis of CREF eigen-modules by random shuffling

To further explore the stability of eigen-modules, we introduce random shuffling of eigen-modules. We randomly shuffle the loadings in the gene-eigenvector and motif-eigenvector that

constitute the eigen-module at a certain level, and then a new CREF matrix is reconstructed and decomposed. The gene- and motif-eigenvectors in the original eigen-module before shuffling consist of loadings in a particular order, which contain part of the important information of this eigen-module. In contrast, the random shuffling of a certain eigen-module eliminates such information. Then, we investigate the relationship between the original eigen-modules and those obtained after random shuffling to evaluate the influence of random shuffling on eigen-modules.

To be clear, we explain it in a mathematical setting. Let C denote the original CREF matrix, we randomly shuffle the loadings in its gene-eigenvector u_p and motif-eigenvector v_p at a certain level p, then the corresponding vectors u_p^* and v_p^* are obtained. Next, the new CREF matrix $\breve{C}$ is constructed and SVD is applied as follows:

$$\breve{C} = \rho_p u_p^* v_p^{*T} + \sum\nolimits_{k \neq p} \rho_k u_k v_k^T,$$

$$\breve{C} = \sum\nolimits_{k} \breve{\rho}_k \breve{u}_k \breve{v}_k^T,$$

where $\breve{u}_k$ and $\breve{v}_k$ represent the new gene- and motif-eigenvectors in the SVD of $\breve{C}$ respectively. At level p, which takes values from one to nine, we repeat the above steps 1000 times to obtain 1000 samples of decomposition. To measure the effects of random shuffling on the eigen-modules at each level, we evaluate the differences among eigen-modules in the following two ways.

Firstly, we compute the mean absolute values of PCCs between the original motif-eigenvectors in the top nine eigen-modules and those obtained after random shuffling. Specifically, for each i and j from one to nine, we compute r_{ij}, the PCC between the i-th original motif-eigenvectors v_i and the j-th new motif-eigenvectors $\breve{v}_j$ after shuffling for each sample. Then we compute the mean of $|r_{ij}|$ of all samples.

For five canids, the mean absolute values of all PCCs are displayed in Fig. S9. In general, strong correlations are observed along the diagonal of each PCC matrix while off-diagonal correlations are much weaker. With a close look at the diagonal elements, we find the mean PCC vanishes only when the level of a new motif-eigenvector in the shuffled eigen-module matches that of the original one; otherwise, the mean PCCs are approximately one. This indicates that the random shuffling of the eigen-module at a certain level only diminishes the correlation between motif-eigenvectors in this module, while the correlations in the other modules remain almost unaffected.

Some off-diagonal PCCs exhibit moderate correlation. We notice that in the case of red foxes, the shuffling of the fourth eigen-module leads to a mean PCC of 0.35 between the original fifth motif-eigenvector and the fourth one obtained after shuffling; in the meantime, the mean PCC at level four reduces to 0.92. Similarly, the shuffling of the fifth eigen-module leads to a mean PCC of 0.34 between the original fourth motif-eigenvector and the fifth one obtained after shuffling; in the meantime, the mean PCC at level five reduces to 0.92. This phenomenon can also be observed when the seventh eigen-module or eighth one is shuffled. For all five canids, when the seventh eigen-module is shuffled, moderate correlations between the original eighth motif-eigenvector and the seventh one obtained after shuffling are observed, ranging from 0.2 to 0.3; when the eighth eigen-module is shuffled, moderate correlations between the original seventh

motif-eigenvector and the eighth one obtained after shuffling are observed in the range from 0.2 to 0.3.

Secondly, at each of the top nine levels, we use the mean Frobenius norm to measure the difference between the original eigen-module matrix and that obtained after shuffling. Specifically, for l from one to nine, we compute the Frobenius norm, d_l, of the difference between the original eigen-module matrix $u_l v_l^T$ and the new eigen-module matrix $\breve{u}_l \breve{v}_l^T$,

$$d_l = \|u_l v_l^T - \breve{u}_l \breve{v}_l^T\|_F.$$

Then we compute the mean of d_l of all samples. Notice that the Frobenius norm of the original and the new eigen-module matrix is equal to one,

$$\|u_l v_l^T\|_F = \|\breve{u}_l \breve{v}_l^T\|_F = 1.$$

This normalization allows for a direct comparison of the mean Frobenius norm across different levels and species. Therefore, the mean Frobenius norm is a reasonable measure to quantify the difference between eigen-modules.

It can be found in Fig. S10 that the difference between eigen-modules at the shuffled level is much larger than those at other levels in most cases of shuffling. Notably, for all five canids and all shuffling, the mean Frobenius norm of such differences is almost equal (about $\sqrt{2}$). Here are two of the few exceptions. First, for red foxes, a difference with mean Frobenius norm 0.49 appears between the original fifth eigen-module and that obtained after shuffling when the fourth eigen-module is shuffled; shuffling the fifth eigen-module also leads to a difference with mean Frobenius norm 0.49 between the original fourth eigen-module and that obtained after shuffling. Second, for each of the five canids, when the seventh eigen-module is shuffled, a difference appears between the original eighth eigen-module and that obtained after shuffling with mean Frobenius norm over 0.29; shuffling the eighth eigen-module also leads to a difference with mean Frobenius norm over 0.29 between the original seventh eigen-module and that obtained after shuffling.

The above influence of random shuffling on eigen-modules is consistent with the results of the relative distance between adjacent singular values and the simulation of motif re-sampling. The relative distance between the fourth and fifth levels is as small as 1.9% in red foxes and less than 5.0% between the seventh and eighth levels in all five canids. In the simulation of motif re-sampling, there are significant overlaps in the distribution of the fourth singular value with the fifth one in red foxes and the distribution of the seventh one and the eighth one in all five canids. These results demonstrate that if two adjacent singular values are far apart, a perturbation in one eigen-module will only influence this module itself to some extent, whereas the other modules remain almost unaffected; if two adjacent singular values are close to each other, then the eigen-modules at these two levels will be unstable and be sensitive to perturbations. The singularity paves the way for saltation.

Enrichment analysis by the Wilcoxon rank sum scoring method

Inferring the biological function of each CREF eigen-module is a key step of dual eigen-analysis. In this step, we perform enrichment analysis on each polarized gene-eigenvector to identify the

significantly enriched biological subsets at the two poles. The relevant gene subsets we consider are collected from the following three knowledgebases: Gene Ontology [8,9], KEGG [10,11], and Reactome [12]. Among them, Gene Ontology gene sets are classified into three groups: Biological Process (BP), Molecular Function (MF), and Cellular Component (CC). To improve the robustness of our analysis, we adopt the rank-based Wilcoxon scoring method [13] as the statistical method for enrichment analysis. The method is described as follows.

Suppose we have gene subsets $S_1, S_2, \cdots, S_n$. Denote the set of genes in the polarized gene-eigenvector by G. First, for each gene subset S_i, we compare the loadings of genes in the intersection $G \cap S_i$ against the loadings of those in relative complement $G - S_i$ by one-sided Wilcoxon rank sum test, where the latter can be regarded as the control group for the former. The p-value of the test is obtained accordingly. As needed in some situations, a one-sided right-tail p-value < 0.10 indicates a significant enrichment at the positive pole; whereas a one-sided left-tail p-value < 0.10 indicates a significant enrichment at the negative pole. We choose 0.10 as the significance level due to the relatively small number of gene sets collected.

Second, we sort these subsets according to their statistical significance. Gene subsets with smaller one-sided right-tail p-values indicate a more significant enrichment at the positive pole; gene subsets with smaller one-sided left-tail p-values indicate a more significant enrichment at the negative pole.

Third, based on the gene subsets significantly enriched at the poles of the gene-eigenvector together with their statistical significance, we select representative biological subsets and summarized the biological function of the CREF eigen-module.

Details of the long-term memory-related pathways significantly enriched at the positive pole of the fourth gene-eigenvector

Along with learning and long-term memory, the positive pole of the dog fourth gene-eigenvector exhibits up-regulated activities of synaptic transmissions. Synapses of several important neurotransmitters are significantly enhanced. This includes glutamate, which serves as the primary neurotransmitter at the great majority of fast excitatory synapses, together with GABA and glycine, the major inhibitory neurotransmitter in the brain and spinal cord. Some other types of synapses can also be found at the positive pole, such as dopaminergic synapse, cholinergic synapse, and serotonergic synapse. Additionally, several biological pathways related to membrane potential changes during neurotransmission are enriched, such as excitatory and inhibitory postsynaptic potential, membrane depolarization, hyperpolarization, and repolarization. Other enriched neurotransmission-related pathways include synaptic vesicle maturation, transport, priming, and postsynaptic membrane, etc.

A crucial neurochemical foundation of learning and memory is synaptic plasticity. Among the various forms of long-term synaptic plasticity, two prominent and most studied forms are long-term potentiation (LTP) and long-term depression (LTD) [14]. It is observed that the regulation of long-term neuronal synaptic plasticity, long-term potentiation, and long-term depression are enriched at the positive pole of the dog fourth gene-eigenvector.

One of our focus is on NMDA receptor-dependent LTP. The primary molecular mechanism of LTP induction involves the NMDA and AMPA ionotropic glutamate receptors, postsynaptic

depolarization, and calcium influx. In a typical scenario, the binding of synaptically released glutamate to NMDA receptors coupled with depolarization of the postsynaptic membrane, which relieves the extracellular magnesium ions channel block, results in the opening of NMDA channels. This leads to an influx of calcium ions through activated NMDA receptors and consequently a rise of calcium ions concentration in dendritic spines. This is necessary for triggering the subsequent biochemical processes that drive LTP. Furthermore, depolarization also opens voltage-dependent calcium channels on the dendritic shafts. Indeed, at the positive pole, we found several significantly enhanced biological pathways related to ionotropic glutamate receptors, voltage-gated calcium channels, and cellular calcium ion concentration.

The primary downstream target of the rise in postsynaptic calcium is calcium-calmodulin-dependent kinase II (CaMKII), which is both necessary and sufficient for LTP. CaMKII undergoes autophosphorylation, which converts it into a constitutively active enzyme independent of calcium and makes it ideally suited to serve as a "memory molecule" [14]. Protein phosphorylation-dephosphorylation is important for LTP. The GluA1-4 subunits of AMPA receptors are phosphorylated on serine, threonine, and tyrosine residues by several protein kinases on over 20 different phosphorylation sites [15,16]. It is observed that protein phosphorylation and protein autophosphorylation are both enhanced at the positive pole of the dog fourth gene-eigenvector, together with phosphorylation of serine, threonine, and tyrosine.

The increase in postsynaptic calcium also results in the adenylyl cyclase-activated cAMP signal. The cAMP signaling pathway can activate downstream protein kinase A and key transcription factors such as CREB, thereby initiating a series of transcriptional events essential for LTP maintenance [17,18]. We notice that the cAMP signaling pathway is also significantly enriched.

Details of the myelination-related pathways significantly enriched at the poles of the fourth gene-eigenvector

The differentiation and development of oligodendrocytes and Schwann cells, which are two types of critical myelin-forming glial cells in the CNS and PNS respectively, are also enriched at the poles of both human and dog fourth gene-eigenvectors. During active myelination phases, oligodendrocytes exhibit high metabolical activity and synthesize large amounts of myelin components, especially lipids [19]. This ensures an extraordinarily high level of lipid synthesis required for effective myelination.

Unlike most other biological membranes, the myelin sheath exhibits a distinctive composition characterized by a high lipid content, accounting for about 75% of its dry weight, and a relatively low protein content of about 20% [20]. For many myelin lipids, their metabolism-related pathways are observed significantly enriched at the poles of both human and dog fourth gene-eigenvectors. They include phospholipid biosynthetic and metabolic process, phospholipid homeostasis, glycerophospholipid biosynthetic and metabolic process, sphingolipid metabolism, and glycosphingolipid metabolism.

Among the various myelin lipids, we first focus on cholesterol. Cholesterol constitutes over 25% of the total lipid content in myelin, in contrast to less than 20% in other typical plasma membranes [21]. Cholesterol is an indispensable lipid component of myelin membranes. A high cholesterol level is essential for myelin membrane growth during brain maturation [21]. In addition

to the general function of providing stability to myelin by regulating both fluidity and permeability of the membrane, the rate of cholesterol synthesis is associated with the speed of myelin membrane biogenesis [19]. It is found that cholesterol biosynthetic process and regulation of cholesterol biosynthetic process are enriched at the poles of both human and dog fourth gene-eigenvectors. Besides, some other biological pathways related to cholesterol metabolism are also enriched, such as cholesterol metabolic process, cholesterol homeostasis, and cholesterol transport.

In addition to cholesterol, fatty acids are another important component of the fundamental structure of myelin, indicating the critical role of fatty acid synthesis in myelination. Surprisingly, fatty acid biosynthesis and fatty acid homeostasis are significantly enhanced at the poles of both human and dog fourth gene-eigenvectors. Other enriched gene sets related to fatty acid metabolism include positive regulation of fatty acid biosynthetic process, fatty acid metabolism, and fatty acid elongation.

Details of the cochlea development-related pathways significantly enriched at the poles of the fourth gene-eigenvector

Cochlea morphogenesis, inner ear morphogenesis, and inner ear development are enriched in humans; cochlea development is enriched in dogs.

In the cochlea, the perception of auditory stimuli is mediated by the organ of Corti, a specialized sensory epithelium that extends alongside the coiled cochlear duct. Mechanosensory hair cells located in the organ of Corti serve as the primary transducers, converting auditory stimuli into neural signals. We found that inner ear receptor cell differentiation and development together with auditory receptor cell stereocilium organization are enriched at the poles of both human and dog fourth gene-eigenvectors.

Several other enriched biological pathways related to cochlea development are also enriched, including vestibulocochlear nerve formation and detection of mechanical stimulus involved in sensory perception of sound.

Details of the long-term memory regulators standing around the positive pole of the fourth motif-eigenvector

EGR1, recognized as a marker of neuronal plasticity, plays a crucial role in the maintenance of synaptic plasticity [22]. Another member of the EGR family, EGR2, was also reported to be involved in the regulation of long-term memory. Their respective binding *cis*-elements, EGR1_04 and EGR2_Q6, rank 25/24 in dogs and 35/38 in humans. Besides, the EGR family has a binding element EGR_Q6 ranking 52 and 128 respectively in dogs and humans.

NFKB, apart from its well-known role in the immune system, assumes an important role in synaptic transmission, regulated synaptic plasticity, and memory formation [23,24]. One of its members, NFKB1, has a binding element P50_Q6 ranking 73 in dogs and 51 in humans.

It is now well accepted that CREB is pivotal in consolidating short-term memory to long-term memory. During the early phase of LTP, the calcium-activated cAMP signaling pathway triggers

the phosphorylation of CREBs, which in turn initiates the expression of a series of downstream transcription factors and genes. Three binding elements, CREBATF_Q6, TAXCREB_01, and TAXCREB_02, stay at the top of the opposite pole, ranking 41, 87, and 44 in dogs, respectively. In humans, their rankings are 71, 105, and 51.

SP1 and MAZ were reported to be involved in mediating the enhancement of NR1 (NMDA receptor subunit type 1) promoter activity during neuronal differentiation [25]. SP1_03 and MAZ_Q6, two of their binding elements, hold rankings of 8/22 in dogs and 14/22 in humans, respectively. Their other binding elements also rank among the top 100.

SP3, along with SP1, serves as an oxidative stress-induced transcription factor in cortical neurons and positively regulates neuronal survival [26]. Its binding element SP3_Q3 ranks 61 in dogs and 86 in humans.

SP4, another member of the SP family, was identified as an important regulator of dendritic patterning during cerebellar development by promoting activity-dependent pruning and limiting branch formation [27]. Aberrant dendritic patterning was found to be associated with neurological disorders and defects in learning and memory [28]. SP4 has a binding element SP4_Q5 ranking 74 in dogs and 181 in humans.

Details of the myelination regulators standing around the poles of the fourth motif-eigenvector

In the central nervous system, the myelin-forming cells are a kind of glial cells called oligodendrocytes. NFIA plays a crucial role in the initiation of gliogenesis. It promotes gliogenesis by forming a Sox9/NFIA complex with Sox9 and co-regulating a set of genes induced after glial initiation [29,30]. MYOGNF1_01, the *cis*-binding element of NFIA, is found at the negative pole of the fourth motif-eigenvector, with a ranking of 33 in dogs and 34 in humans.

SOXD proteins keep oligodendrocytes in the precursor state, inhibit terminal differentiation, and promote their migratory activity [31,32]. In contrast, SOXD proteins promote differentiation and myelination of Schwann cells in PNS [33]. Among the SOXD family, we found a member SOX13. It has a binding element SOX13_04 ranking in the first place at the positive pole in both dogs and humans. SOX13 was reported to functionally complement SOX5 and SOX6, the other two members of the SOXD family, as important modulators of oligodendrocyte development [34].

PAX3 is important for the early development of the Schwann cell lineage and is considered to be involved in the differentiation pathway to myelinating Schwann cells in PNS myelination [35]. The corresponding spontaneous Splotch and Splotch-delayed mouse mutants for Pax3 exhibit early Schwann cell defects [36]. PAX3 has a binding element PAX3_B at the negative pole with a ranking of 9 in both dogs and humans. Another binding partner, PAX3_01, ranks 25 and 92 in dogs and humans, respectively.

AP-2 transcription factor is suggested to be involved in the control of the timing of Schwann cell development as a negative regulator of Schwann cell generation [37]. Two of its binding elements, AP2_Q3 and AP2_Q6_01, both rank among the top 20 at the negative pole in dogs and humans.

The activation of NFKB was shown to be an essential differentiation signal for the progression of axon-associated Schwann cells into a myelinating phenotype [38]. It is upstream of OCT6

induction in Schwann cells and OCT6 is a pivotal regulator in PNS myelination regulatory network. One of its members, NFKB1, has a binding element P50_Q6 at the positive pole, ranking 73 in dogs and 51 in humans.

Transcription factor EGR2 (Krox20) is widely regarded as one of the cornerstones and central regulators of peripheral myelination [39]. During the myelination process of PNS, OCT6 is activated by SOX10 at first and then stimulates EGR2 expression in synergy with SOX10, which forms a positive feed-forward loop. Besides the onset of myelination, EGR2 is also crucial for the maintenance of the myelinating state and may act as a molecular switch governing Schwann cell myelination and demyelination programs [40]. EGR2_Q6, the binding element of EGR2, is found at the top of the positive pole, ranking 24 in dogs and 38 in humans. SOX10_01, the binding element of SOX10, is found at the top of the ninth level, ranking 207 in dogs and 201 in humans.

Genome-wide analysis has shown that EGR2 extensively binds to regulatory regions of myelin-associated genes, including several genes involved in lipid biosynthesis and metabolism [41]. SREBP is the central regulator of lipid biosynthesis and plays a critical role in the peripheral nerve myelination process. It was reported that EGR2 and SREBP transactivators can synergistically activate promoters of several SREBP target genes [42]. Its binding *cis*-element, SREBP1_02, is found at the positive pole in both dogs and humans, ranking 19 and 63 respectively. We note that the regulation of cholesterol biosynthesis by SREBP (SREBF) is correspondingly enriched at the same pole of the human fourth gene-eigenvector.

Details of the cochlea development regulators standing around the poles of the fourth motif-eigenvector

The transcription factor PKNOX2 was shown to play a critical role in regulating the sensitivity of cochlea at higher frequencies in mammals [43]. Its binding *cis*-element, PKNOX2_01, is found at the negative pole in both dogs and humans, with respective ranks 26 and 30.

The zinc finger protein HELIOS, encoded by the IKZF2 gene, was identified as an essential transcription factor required for functional maturation of cochlea outer hair cells and hearing [44]. Mice with a mutation in the IKZF2 gene exhibit progressive hearing loss and defects in outer hair cell function. HELIOS has a binding element HELIOSA_01 at the positive pole, ranking 56 in dogs and 33 in humans. Another binding partner, HELIOSA_02, ranks 70 in dogs and 21 in humans.

OTX1 was reported to be involved in the morphogenesis of the inner ear and the development of its sensory organs [45]. The absence of OTX1 in mice affected the shaping of the cochlea and the development of some sensory organs within the inner ear. OTX1_01, the binding element of OTX1, is found at the positive pole in both dogs and humans, with rankings of 98 in dogs and 79 in humans.

PAX3 not only participates in myelination but also contributes extensively to the development of multiple inner ear structures. The function of PAX3 is specifically required for differentiation and survival of the cochlea melanogenic lineage, whose absence appears to be a major cause of congenital hearing loss in human Waardenburg's syndrome type I (WS-I)[46,47]. As mentioned

earlier, PAX3 has two binding elements ranking among the top 100 at the negative pole in both dogs and humans.

MAFB was found to drive the formation of auditory ribbon synapses, specialized structures facilitating rapid transmission from cochlea hair cells to spiral ganglion neurons [48]. Loss of MAFB function in both humans and mice was reported to cause inner-ear defects and deafness [49]. MAFB has a binding element MAFB_01 at the negative pole, ranking 95 in dogs and 22 in humans.

Evidence supporting the hypothesis of the saltation near the degenerate point

We propose that the red fox is near the degenerate point of the saltation. This is supported by several observations and simulations as below.

First, the fourth and fifth singular values of red foxes are extremely close. The relative distance between them is as small as 1.9%, which is far less than that between other adjacent pairs in the top six levels (Table S2). Among five canids, the relative distance between the fourth and fifth singular values of red foxes is the smallest, and it is also smaller than that of humans, chimpanzees, and orangutans [1].

Second, the orthogonal projections of the fourth and fifth motif-eigenvectors of different breeds of dogs onto the 2-D eigenspace of red foxes show that although the 2-D eigenspaces of dogs and red foxes are almost identical, the fourth and fifth motif-eigenvectors of dogs rotate at a relatively large angle (between 72° and 75°) from those of red foxes (Fig. S12). In addition to dogs, the projections of dingoes and dholes also exhibit large rotations (about 62° and 72°) with reference to red foxes (Fig. S12). In contrast, with reference to wolves, the fourth and fifth motif-eigenvectors of red foxes only rotate at a small angle (about 10°), as shown in Fig. S7. Putting together the results of projections, we propose that the red fox has just passed the degenerate point and its fourth and fifth levels have not been fully separated yet.

In addition, two simulation results also support our speculation. First, the stability analysis of the singular values by motifs re-sampling without replacement shows that the sampling distributions of the fourth and fifth singular values overlap most in red foxes among five canids (Fig. S8). Second, among five canids, the random shuffling of each of the fourth and fifth eigen-modules induces the strongest interaction in red foxes (section “Stability analysis of CREF eigen-modules by random shuffling” in the Supplementary Text, Fig. S9, and Fig. S10).

Motivations for studying Can-SINE elements

Our previous study has suggested that one of the important driving factors of Hominidae genomic saltation is mutations related to Alu elements [1,50]. The relationship was found primarily through the *cis*-motifs present on Alu elements.

Similar to humans, transposable elements account for a large proportion of the dog genome (about 34 to 40 percent) [51,52]. Short interspersed nuclear elements (SINEs) are the most abundant transposable elements in terms of copy number (about 1.5 million copies) and occupy around 10% of the dog genome [51,52]. Can-SINEs, a Carnivora-specific SINE family, have become the predominant SINEs within Carnivora genomes and serve as a significant source of genomic

diversity. Based on the dog genome, the estimated number of Can-SINEs is 1.1 million [51]. Some of the Can-SINE elements still exhibit high retrotransposition activity in the dog genome [52]. For example, Can-SINE sub-type SINEC_Cf is still active today as indicated by the presence of numerous recent insertions that exhibit polymorphism [53]. For the above reasons, we explore the relationship between Can-SINE elements and the saltation of Canidae CREF eigen-modules.

Frequency comparison of MPCS and other motifs at the poles of the fourth and fifth motif-eigenvectors

In order to show that mutations related to Can-SINE elements account partially for the saltation of Canidae CREF eigen-modules, we first compare the frequencies of MPCSs (motifs present on Can-SINE elements) and those of other motifs among the top 100 at the two poles.

For a polarized motif-eigenvector at a certain level of a certain species, the total of 1403 motifs can be divided into four categories according to whether they are MPCSs and whether they occur among the top 100. In the case of the MPCSs output by MATCH program with the minSUM option, a 2 × 2 contingency table shown in Table S9 displays the numbers of motifs belonging to such four categories.

The odds ratio (OR) is a simple measure of the strength of the association between the two events corresponding to the row and the column factor in a 2 × 2 contingency table. In this table, the OR refers to the ratio of the odds of MPCS occurrences and the odds of other motifs occurrences among the top 100. The sample OR is the ratio of the product of the main diagonal elements and the product of the off-diagonal elements. Namely,

$$OR = \frac{n_{11}/(n_{11}+n_{10})}{n_{10}/(n_{11}+n_{10})} \Big/ \frac{n_{01}/(n_{01}+n_{00})}{n_{00}/(n_{01}+n_{00})} = \frac{n_{11}n_{00}}{n_{10}n_{01}}.$$

Based on the contingency table, we want to know whether the odds of MPCS occurrences among the top 100 increase compared to other motifs. Notice that an OR of 1 is equivalent to no association between the two events, and an OR greater than 1 indicates a positive association between two events in the sense that compared to the absence of one event, the presence of this event raises the odds of the other event. Therefore, we consider the following hypotheses (one-sided alternative).

$$H_0: OR = 1 \leftrightarrow H_1: OR > 1$$

Acceptance of the null hypothesis indicates that there is no significant difference between the odds of MPCS occurrences among the top 100 and that of other motifs; rejection of the null hypothesis indicates that the odds of MPCS occurrences among the top 100 are significantly greater than that of other motifs.

Taking into account that both the total number of MPCSs and the total number of motifs in the top 100 are fixed, we perform Fisher's exact test (one-sided) [54,55]. The p-value of the test is provided. Besides, we also give the estimate (conditional maximum likelihood estimate) and the 95 percent lower confidence bound of OR.

Contingency tables and test results for five canids at levels four and five are presented in Table S10, based on the MPCSs output by the MATCH program with the minSUM option. At the fourth level, it is obvious that the odds of MPCS occurrences among the top 100 increase significantly for dogs, dingoes, and dholes, with p-values 6.88e-8, 1.77e-8, and 2.97e-6

respectively. At the fifth level, the odds in red foxes and wolves are significantly enhanced with p-values 2.97e-6 and 9.42e-6.

MPCSs at the top of polarized motif-eigenvectors with the MATCH program option set to minFP

An alternative determination of MPCSs is setting the MATCH program option to minFP (minimize false positive rate). Compared to the minSUM threshold option, the minFP threshold option increases the false negative rate while reducing the false positive rate, allowing us to find more reliable transcription factor binding sites [56]. With the minFP option, the MATCH program output 22 MPCSs.

Similarly, for five canids, we count the number of MPCSs among the top 100 motifs at the two poles of each level, as shown in Fig. S14. The ranks of 22 MPCSs in the fourth and fifth motif-eigenvectors can be found in Table S12.

We compare the frequencies of MPCSs and those of other motifs among the top 100 at the two poles by Fisher's exact test (one-sided). For details of Fisher's exact test, see Table S11. At level four, the odds of MPCS occurrences among the top 100 increase substantially for dogs, dingoes, and dholes, with p-values 1.07e-6, 9.01e-6, and 3.78e-4 respectively. While at level five, MPCS occurrences of red foxes and wolves are significantly enhanced with p-values 3.78e-4 and 2.76e-2. This result is consistent with that obtained by the minSUM option.

We also calculate the relative change of MPCSs in percentages at each level from wolves to dogs, as shown in Fig. S15. The number of MPCSs increases most prominently at the fourth level by 160.0%.

Polarization degree of eigenvectors

In this section we define a metric, polarization degree, to quantitatively measure the concentration of the gene or motif loadings near the two poles of an eigenvector. The polarized eigenvector can be a polarized gene-eigenvector or a polarized motif-eigenvector.

In the definition of polarization, the importance of a gene or a motif in a CREF eigen-module is measured by its square, which is referred to as its energy. All energies sum to one according to the eigenvector definition.

Consider a polarized eigenvector denoted by $\vec{w}$, we define the polarization degree of its positive end and negative end, respectively. Let $\vec{w} = \left(w_1^+, w_2^+, \dots, w_p^+, 0, 0, \dots, 0, w_n^-, \dots, w_2^-, w_1^-\right)^T$, where p and n denote the number of positive and negative loadings in $\vec{w}$, respectively. The loadings in $\vec{w}$ are sorted in descending order: $w_1^+ \geq w_2^+ \geq \cdots \geq w_p^+ > 0 > w_n^- \geq \cdots \geq w_2^- \geq w_1^-$. Below we only give the definition of its positive end polarization degree $d^+(\vec{w})$. The negative end polarization degree $d^-(\vec{w})$ can be defined in the same way.

First, it is convenient to give the following definitions and notations. At the positive end, the energy of the ith gene or motif is denoted by e_i^+:

$$e_i^+ = {w_i^+}^2, \quad i = 1,2,\cdots,p.$$

We can normalize the energy by computing its proportion in the total energy at the positive end: e_i^+/e^+, where e^+ is the total energy at the positive end. Namely, e^+ is the sum of the squares of all positive loadings:

$$e^+ = \sum_{i=1}^{p} e_i^+ = \sum_{i=1}^{p} {w_i^+}^2.$$

Note that since the length of each eigenvector is one, the sum of the total energy at the positive and the negative end is one. The rank of the loading can also be normalized by a linear mapping from $\{1,2,\cdots,m\}$ to the interval $[0,1]$:

$$r_i^+ = \frac{i}{p}, \qquad i = 1,2,\cdots,p.$$

Now we can define the positive end polarization degree of $\vec{w}$ as follows.

Polarization degree For a polarized gene- or motif-eigenvector $\vec{w}$, the polarization degree of its positive end is defined as

$$d^+(\vec{w}) = \sum_{i=1}^{k^+} \frac{e_i^+}{e^+},$$

where

$$k^+ = \min_{1\le k\le p}\left\{\sum_{i=k}^{p} \frac{e_i^+}{e^+} + r_k^+ \ge 1\right\} = \min_{1\le k\le p}\left\{\sum_{i=k}^{p} \frac{e_i^+}{e^+} \ge 1 - r_k^+\right\}.$$

.

Fig. S16 intuitively interprets the above definition of polarization degree at the positive end. The gray rectangles with increasing height from left to right show the gradual accumulation of normalized energy from the positive end. Such growth trend of the cumulative normalized energy is outlined by the smooth curve successively connecting the right top of each rectangle. The polarization of $\vec{w}$ indicates that the rate of normalized energy accumulation decreases gradually. Therefore, the curve is concave, in other words, the slope of the tangent to the curve decreases gradually from left to right. Intersecting the curve is a straight line of slope -1. Each point on the straight line satisfies that the sum of the normalized rank and the cumulative normalized energy is exactly one. According to the above definition, the positive end polarization degree of $\vec{w}$ is the height of the first rectangle that intersects with the straight line from left to right, filled in red. In practice, both p and k^+ are relatively so large that are almost of the same magnitude as the number of genes or the number of motifs. Therefore, the polarization degree is approximately equal to the y-coordinate of the intersection of the curve and the straight line. If loadings with relatively large absolute sizes are closer to the positive end, the curve will be closer to the point $(0,1)$, and its intersection with the straight line will also be closer to the point $(0,1)$, which implies a larger polarization degree.

This geometric representation gives an interpretation of the polarization degree. For example, Suppose the polarization degree at the positive end is $d^+(\vec{w}) = 0.9$, then $1 - d^+(\vec{w}) = 10\%$ genes or motifs at the positive end of $\vec{w}$ contain at least $d^+(\vec{w}) = 90\%$ of the total energy of all genes or motifs at the positive end of $\vec{w}$. Therefore, the polarization degree reasonably measures the concentration of energy at the end of an eigenvector. A large polarization degree indicates a higher concentration of energy at this end, and vice versa. Note that the polarization degree is a

real number between 0.5 and 1. Two extremes are helpful to understand the definition. In one extreme when $w_1^+ = w_2^+ = \cdots = w_m^+$, the energy is uniformly distributed at the positive end, and the polarization degree takes the minimum of 0.5; in the other extreme when $w_1^+ \gg w_2^+, \ldots, w_m^+$, the energy is most concentrated at the positive end, and the polarization degree is about the maximum of 1.

References

1. Li, L., Zhang, S. & Li, L. M. Dual Eigen-modules of Cis-Element Regulation Profiles and Selection of Cognition-Language Eigen-direction along Evolution in Hominidae. *Molecular Biology and Evolution* **37**, 1679–1693 (2020).
2. Xu, H., Sepúlveda, L. A., Figard, L., Sokac, A. M. & Golding, I. Combining protein and mRNA quantification to decipher transcriptional regulation. *Nat Methods* **12**, 739–742 (2015).
3. Doughty, B. R. *et al.* Single-molecule chromatin configurations link transcription factor binding to expression in human cells. Preprint at https://doi.org/10.1101/2024.02.02.578660 (2024).
4. Feng, Y., Zhang, S., Li, L. & Li, L. M. The cis-trans binding strength defined by motif frequencies facilitates statistical inference of transcriptional regulation. *BMC Bioinformatics* **20**, 201 (2019).
5. Cheng, C., Yan, X., Sun, F. & Li, L. M. Inferring activity changes of transcription factors by binding association with sorted expression profiles. *BMC Bioinformatics* **8**, 452 (2007).
6. Martin, F. J. *et al.* Ensembl 2023. *Nucleic Acids Research* **51**, D933–D941 (2023).
7. Stewart, G. W. (Gilbert W.). *Matrix Perturbation Theory*. (Academic Press, Boston, 1990).
8. Ashburner, M. *et al.* Gene Ontology: tool for the unification of biology. *Nat Genet* **25**, 25–29 (2000).

9. The Gene Ontology Consortium *et al.* The Gene Ontology knowledgebase in 2023. *Genetics* **224**, iyad031 (2023).
10. Kanehisa, M., Furumichi, M., Tanabe, M., Sato, Y. & Morishima, K. KEGG: new perspectives on genomes, pathways, diseases and drugs. *Nucleic Acids Res* **45**, D353–D361 (2017).
11. Kanehisa, M., Furumichi, M., Sato, Y., Kawashima, M. & Ishiguro-Watanabe, M. KEGG for taxonomy-based analysis of pathways and genomes. *Nucleic Acids Research* **51**, D587–D592 (2023).
12. Milacic, M. *et al.* The Reactome Pathway Knowledgebase 2024. *Nucleic Acids Research* **52**, D672–D678 (2024).
13. Cheng, C. *et al.* Significant and Systematic Expression Differentiation in Long-Lived Yeast Strains. *PLOS ONE* **2**, e1095 (2007).
14. Huganir, R. L. & Nicoll, R. A. AMPARs and Synaptic Plasticity: The Last 25 Years. *Neuron* **80**, 704–717 (2013).
15. Lu, W. & Roche, K. W. Posttranslational regulation of AMPA receptor trafficking and function. *Current Opinion in Neurobiology* **22**, 470–479 (2012).
16. Shepherd, J. D. & Huganir, R. L. The Cell Biology of Synaptic Plasticity: AMPA Receptor Trafficking. *Annual Review of Cell and Developmental Biology* **23**, 613–643 (2007).
17. Davis, S., Vanhoutte, P., Pagès, C., Caboche, J. & Laroche, S. The MAPK/ERK Cascade Targets Both Elk-1 and cAMP Response Element-Binding Protein to Control Long-Term Potentiation-Dependent Gene Expression in the Dentate Gyrus In Vivo. *J. Neurosci.* **20**, 4563–4572 (2000).

18. Elgersma, Y. & Silva, A. J. Molecular mechanisms of synaptic plasticity and memory. *Current Opinion in Neurobiology* **9**, 209–213 (1999).

19. Schmitt, S., Cantuti Castelvetri, L. & Simons, M. Metabolism and functions of lipids in myelin. *Biochimica et Biophysica Acta (BBA) - Molecular and Cell Biology of Lipids* **1851**, 999–1005 (2015).

20. Williams, K. A., Deber, C. M. & Klrschner, O. A. The Structure and Function of Central Nervous System Myelin. *Critical Reviews in Clinical Laboratory Sciences* **30**, 29–64 (1993).

21. Saher, G. *et al.* High cholesterol level is essential for myelin membrane growth. *Nat Neurosci* **8**, 468–475 (2005).

22. Alberini, C. M. Transcription Factors in Long-Term Memory and Synaptic Plasticity. *Physiological Reviews* **89**, 121–145 (2009).

23. Merlo, E., Freudenthal, R., Maldonado, H. & Romano, A. Activation of the transcription factor NF-κB by retrieval is required for long-term memory reconsolidation. *Learn. Mem.* **12**, 23–29 (2005).

24. Kaltschmidt, B. & Kaltschmidt, C. NF-KappaB in Long-Term Memory and Structural Plasticity in the Adult Mammalian Brain. *Frontiers in Molecular Neuroscience* **8**, (2015).

25. Okamoto, S., Sherman, K., Bai, G. & Lipton, S. A. Effect of the ubiquitous transcription factors, SP1 and MAZ, on NMDA receptor subunit type 1 (NR1) expression during neuronal differentiation. *Molecular Brain Research* **107**, 89–96 (2002).

26. Ryu, H. *et al.* Sp1 and Sp3 Are Oxidative Stress-Inducible, Antideath Transcription Factors in Cortical Neurons. *J. Neurosci.* **23**, 3597–3606 (2003).

27. Ramos, B., Gaudillière, B., Bonni, A. & Gill, G. Transcription factor Sp4 regulates dendritic patterning during cerebellar maturation. *Proceedings of the National Academy of Sciences* **104**, 9882–9887 (2007).

28. Kaufmann, W. E. & Moser, H. W. Dendritic Anomalies in Disorders Associated with Mental Retardation. *Cerebral Cortex* **10**, 981–991 (2000).

29. Kang, P. *et al.* Sox9 and NFIA Coordinate a Transcriptional Regulatory Cascade during the Initiation of Gliogenesis. *Neuron* **74**, 79–94 (2012).

30. Deneen, B. *et al.* The Transcription Factor NFIA Controls the Onset of Gliogenesis in the Developing Spinal Cord. *Neuron* **52**, 953–968 (2006).

31. Stolt, C. C. *et al.* SoxD Proteins Influence Multiple Stages of Oligodendrocyte Development and Modulate SoxE Protein Function. *Developmental Cell* **11**, 697–709 (2006).

32. Baroti, T. *et al.* Transcription factors Sox5 and Sox6 exert direct and indirect influences on oligodendroglial migration in spinal cord and forebrain. *Glia* **64**, 122–138 (2016).

33. Ittner, E. *et al.* SoxD transcription factor deficiency in Schwann cells delays myelination in the developing peripheral nervous system. *Sci Rep* **11**, 14044 (2021).

34. Baroti, T., Schillinger, A., Wegner, M. & Stolt, C. C. Sox13 functionally complements the related Sox5 and Sox6 as important developmental modulators in mouse spinal cord oligodendrocytes. *Journal of Neurochemistry* **136**, 316–328 (2016).

35. Kioussi, C., Gross, M. K. & Gruss, P. Pax3: A paired domain gene as a regulator in PNS myelination. *Neuron* **15**, 553–562 (1995).

36. Epstein, D. J., Vekemans, M. & Gros, P. splotch (Sp2H), a mutation affecting development of the mouse neural tube, shows a deletion within the paired homeodomain of Pax-3. *Cell* **67**, 767–774 (1991).

37. Stewart, H. J. S. *et al.* Developmental regulation and overexpression of the transcription factor AP-2, a potential regulator of the timing of Schwann cell generation. *European Journal of Neuroscience* **14**, 363–372 (2001).

38. Nickols, J. C., Valentine, W., Kanwal, S. & Carter, B. D. Activation of the transcription factor NF-κB in Schwann cells is required for peripheral myelin formation. *Nat Neurosci* **6**, 161–167 (2003).

39. Topilko, P. *et al.* Krox-20 controls myelination in the peripheral nervous system. *Nature* **371**, 796–799 (1994).

40. Decker, L. *et al.* Peripheral Myelin Maintenance Is a Dynamic Process Requiring Constant Krox20 Expression. *J. Neurosci.* **26**, 9771–9779 (2006).

41. Jang, S.-W. *et al.* Locus-wide identification of Egr2/Krox20 regulatory targets in myelin genes. *Journal of Neurochemistry* **115**, 1409–1420 (2010).

42. LeBlanc, S. E. *et al.* Regulation of cholesterol/lipid biosynthetic genes by Egr2/Krox20 during peripheral nerve myelination. *Journal of Neurochemistry* **93**, 737–748 (2005).

43. Trigila, A. P. *et al.* Accelerated Evolution Analysis Uncovers PKNOX2 as a Key Transcription Factor in the Mammalian Cochlea. *Molecular Biology and Evolution* **40**, msad128 (2023).

44. Chessum, L. *et al.* Helios is a key transcriptional regulator of outer hair cell maturation. *Nature* **563**, 696–700 (2018).

45. Morsli, H. *et al.* Otx1 and Otx2 activities are required for the normal development of the mouse inner ear. *Development* **126**, 2335–2343 (1999).

46. Kim, H. *et al.* Pax3 function is required specifically for inner ear structures with melanogenic fates. *Biochemical and Biophysical Research Communications* **445**, 608–614 (2014).
47. Udagawa, T. *et al.* Loss of Pax3 causes reduction of melanocytes in the developing mouse cochlea. *Sci Rep* **14**, 2210 (2024).
48. Yu, W.-M. *et al.* A Gata3–Mafb transcriptional network directs post-synaptic differentiation in synapses specialized for hearing. *eLife* **2**, e01341 (2013).
49. Park, J. G. *et al.* Loss of MAFB Function in Humans and Mice Causes Duane Syndrome, Aberrant Extraocular Muscle Innervation, and Inner-Ear Defects. *The American Journal of Human Genetics* **98**, 1220–1227 (2016).
50. Li, L. M., Li, M. & Li, L. Cis-regulatory Element Frequency Modules and their Phase Transition across Hominidae. in *Handbook of Statistical Bioinformatics* (eds. Lu, H. H.-S., Schölkopf, B., Wells, M. T. & Zhao, H.) 371–395 (Springer, Berlin, Heidelberg, 2022). doi:10.1007/978-3-662-65902-1_18.
51. Broad Sequencing Platform members *et al.* Genome sequence, comparative analysis and haplotype structure of the domestic dog. *Nature* **438**, 803–819 (2005).
52. Peng, C. *et al.* Can-SINE dynamics in the giant panda and three other Caniformia genomes. *Mobile DNA* **9**, 32 (2018).
53. Wang, W. & Kirkness, E. F. Short interspersed elements (SINEs) are a major source of canine genomic diversity. *Genome Res.* **15**, 1798–1808 (2005).
54. Fisher, R. A. On the Interpretation of $\chi 2$ from Contingency Tables, and the Calculation of P. *Journal of the Royal Statistical Society* **85**, 87–94 (1922).

55. Edwards, A. W. F. Chapter 67 - R.A. Fischer, statistical methods for research workers, first edition (1925). in *Landmark Writings in Western Mathematics 1640-1940* (eds. Grattan-Guinness, I., Cooke, R., Corry, L., Crépel, P. & Guicciardini, N.) 856–870 (Elsevier Science, Amsterdam, 2005). doi:10.1016/B978-044450871-3/50148-0.

56. Kel, A. E. *et al.* MATCHTM: a tool for searching transcription factor binding sites in DNA sequences. *Nucleic Acids Res* **31**, 3576–3579 (2003).

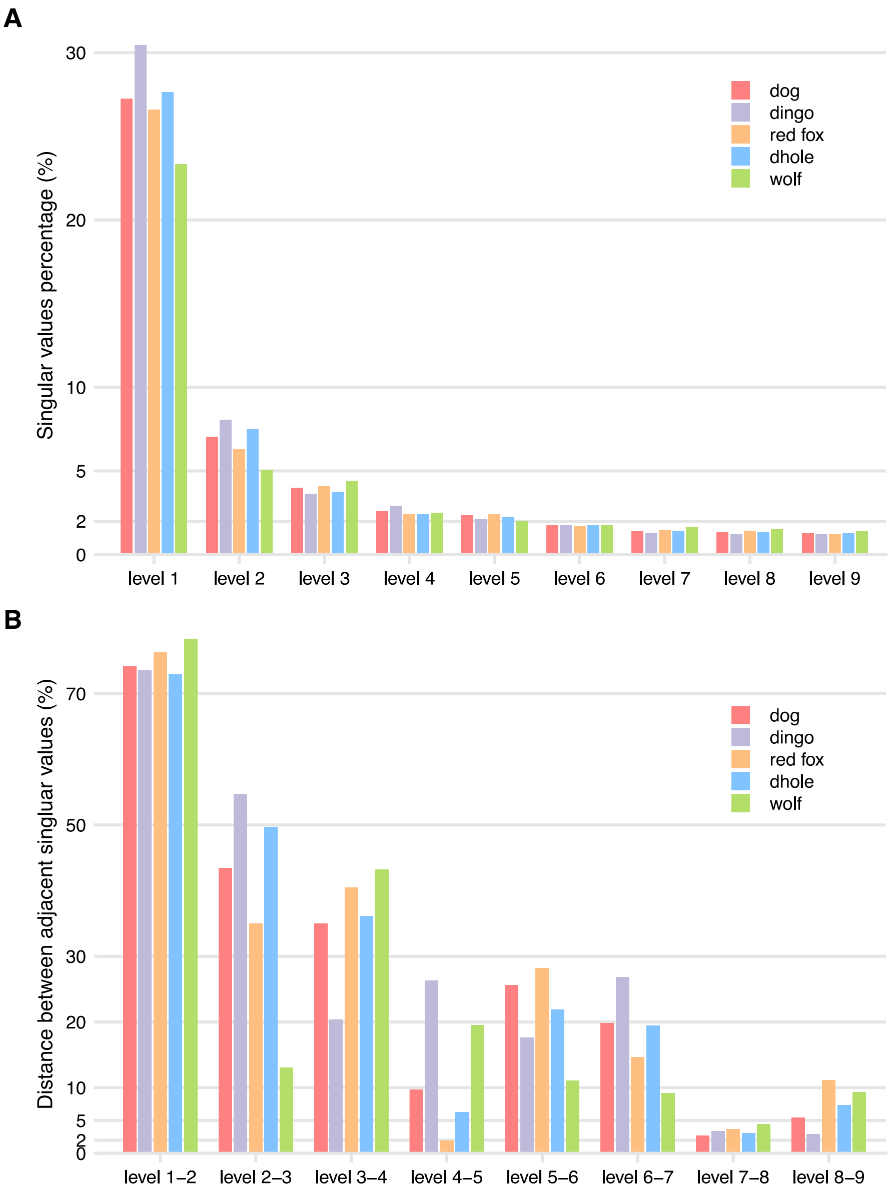

Fig. S1.

(A) Bar chart: the percentages of the top nine singular values of the five canids' CREF matrices. For all five canids, the distributions of their singular values exhibit a long-tailed trend. The percentages of the singular values are less than 5% after the second and less than 2% after the fifth one. **(B) Bar chart: the relative distance between adjacent singular values up to the ninth level.** The relative distance is defined as the ratio of the difference between adjacent singular values over the larger one. Between the fourth and fifth levels, the relative distance is as small as 1.9% in red foxes, 6.3% in dholes, and 9.7% in dogs (Boxer). These values are far less than those between other adjacent pairs in the top six levels (exceeding 20%). In the cases of all five canids, the relative distance between the seventh and eighth levels is less than 5.0% and is less than 12% between the eighth and ninth levels**.**

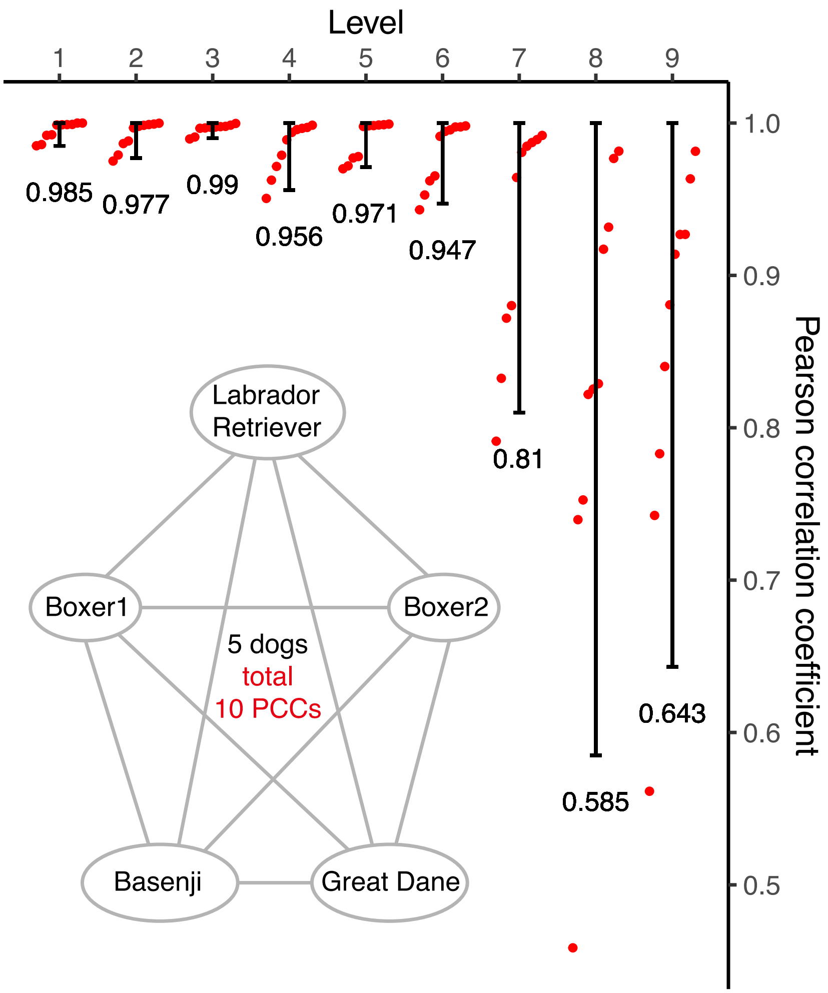

Fig. S2.

The distribution of PCCs (Pearson correlation coefficients) between each pair of five dogs' motif-eigenvectors at the top nine levels. At each level, next to the PCCs in red are their empirical 95 percent confidence intervals in black. Below the intervals are the values of the corresponding confidence lower limits, which are taken as the threshold to define conservation. Roughly speaking, as the level increases, the threshold of conservation shows a decreasing trend.

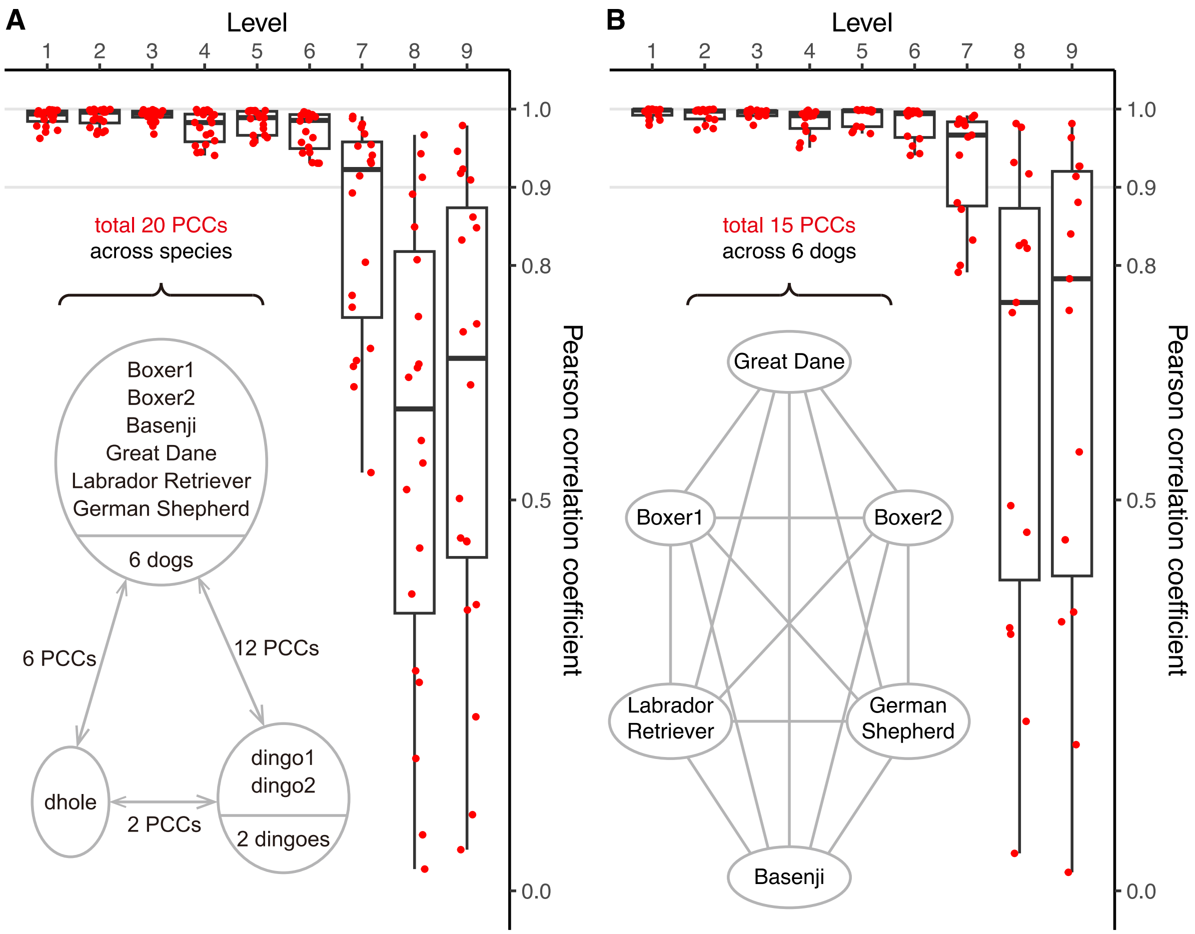

Fig. S3.

(A) The boxplots of PCCs (Pearson correlation coefficients) between each pair of motif-eigenvectors across dogs, dingoes, and dholes at the top nine levels. Dogs, dingoes, and dholes are highly correlated up to the sixth level, as all PCCs are beyond 0.9. **(B) The boxplots of PCCs between each pair of motif-eigenvectors of six dogs at the top nine levels.** The six dogs include five breeds: Boxer, Basenji, Great Dane, Labrador Retriever, and German Shepherd.

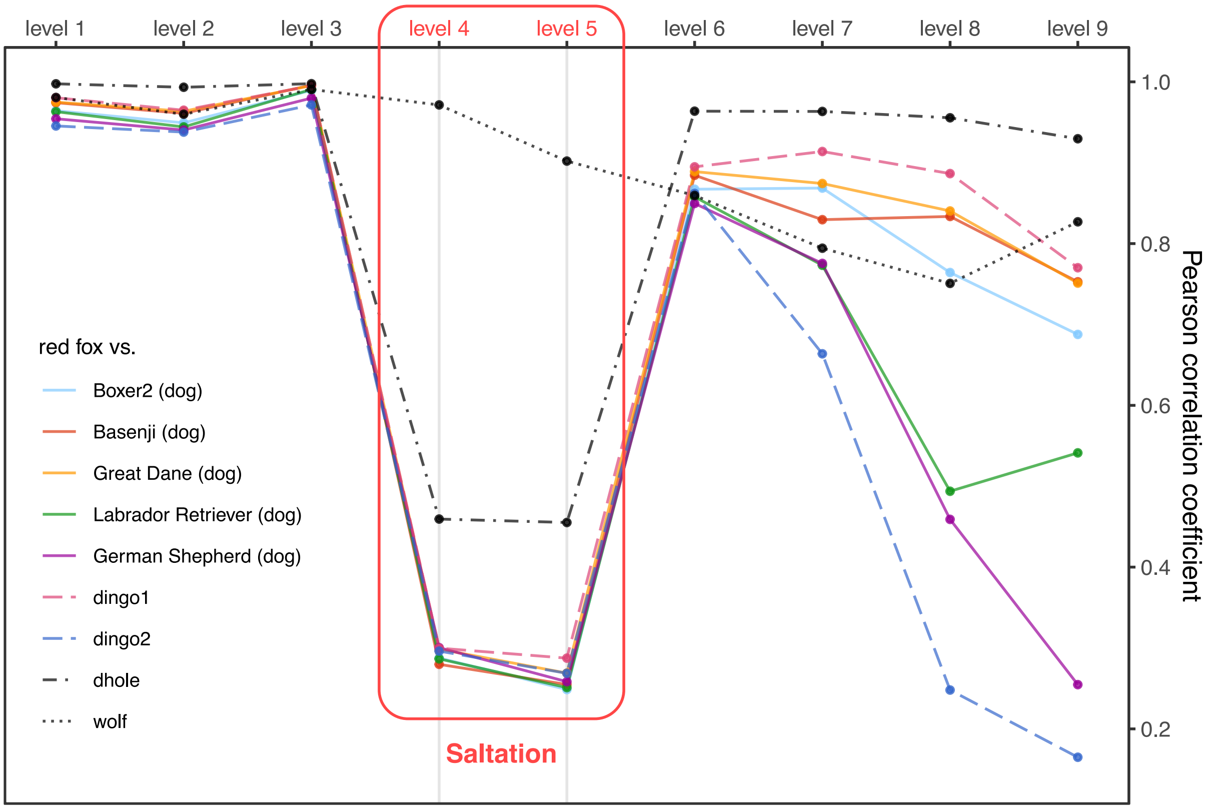

Fig. S4.

The line chart of PCCs (Pearson correlation coefficients) between red foxes' top nine motif-eigenvectors and those of the other nine canids. The nine canids include five breeds of dogs, two dingoes, a dhole, and a wolf. At the fourth and fifth levels, red foxes exhibit a weak correlation (PCC lower than 0.5) with all nine canids except for wolves. This divergence indicates the saltation occurred between the fourth and fifth modules of Canidae.

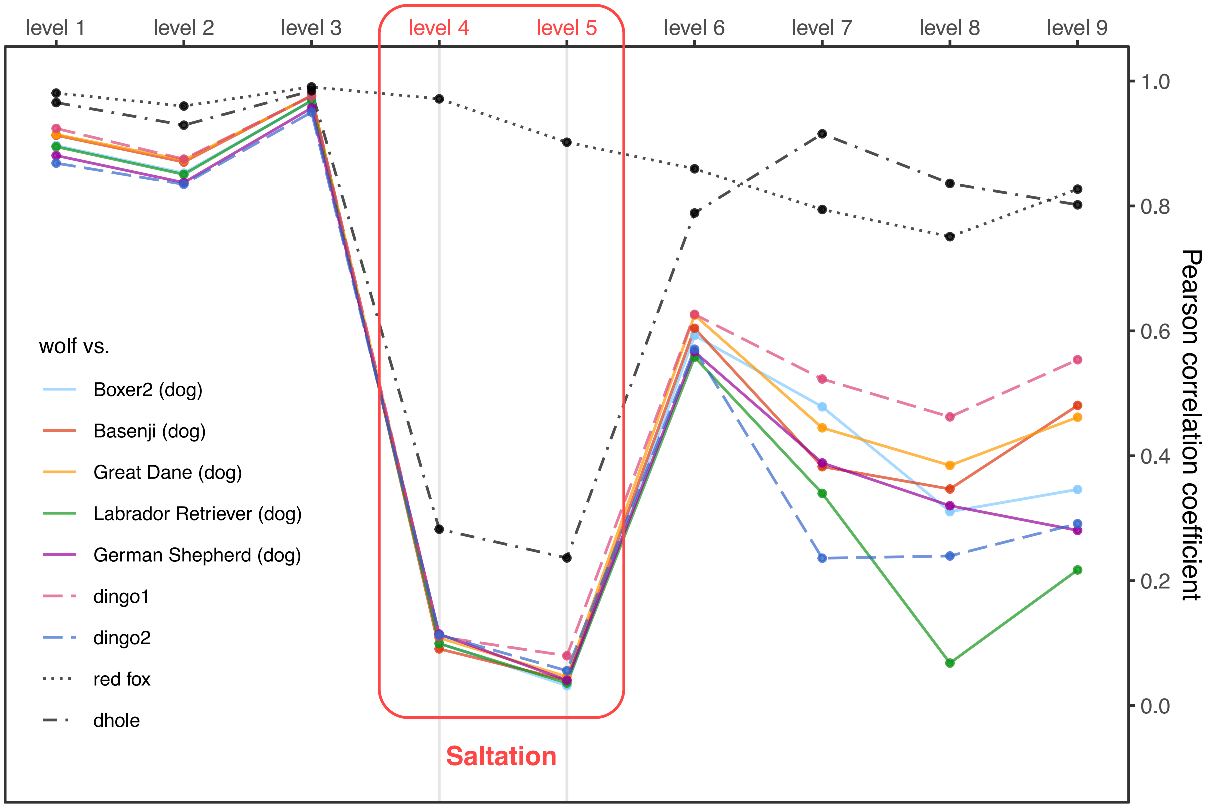

Fig. S5.

The line chart of PCCs (Pearson correlation coefficients) between wolves' top nine motif-eigenvectors and those of the other nine canids. The nine canids include five breeds of dogs, two dingoes, a red fox, and a dhole. At the fourth and fifth levels, wolves exhibit a weak correlation (PCC lower than 0.3) with all nine canids except for red foxes. This divergence indicates the saltation occurred between the fourth and fifth modules of Canidae.

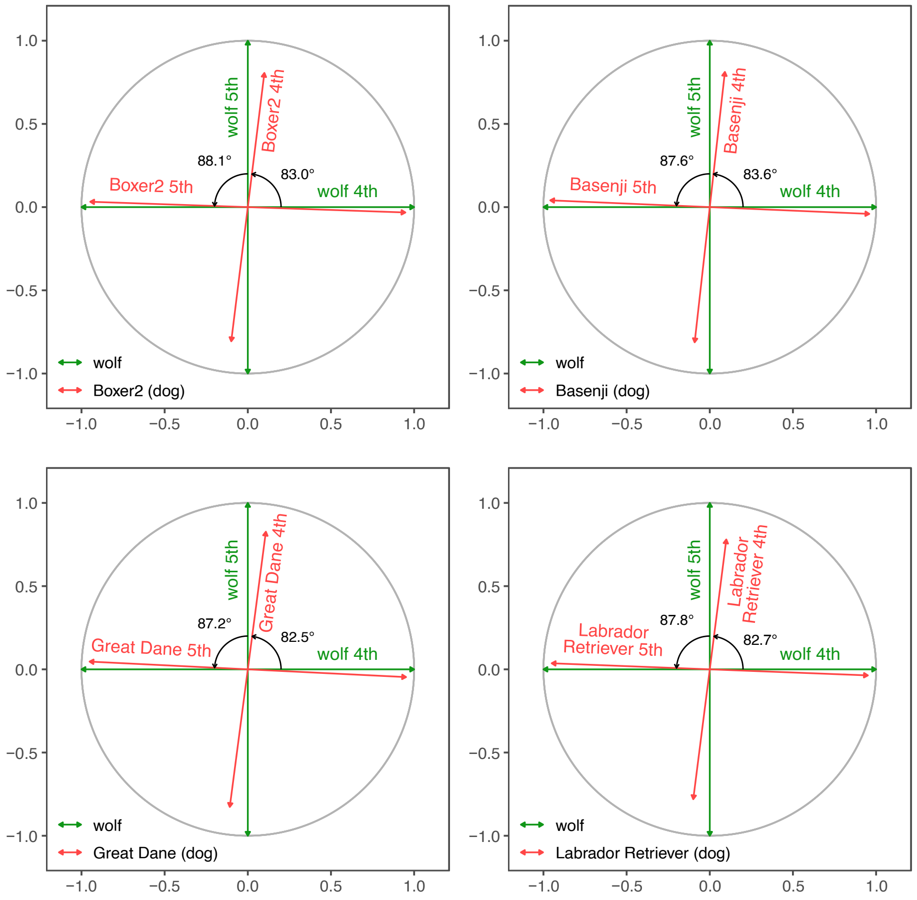

Fig. S6.

Rotations between the fourth and fifth motif-eigenvectors from wolves to four breeds of dogs. In the 2-D eigen-space spanned by the fourth and fifth motif eigenvectors of wolves, the two eigen-directions of all four breeds of dogs rotate about 82° to 88° from those of wolves.

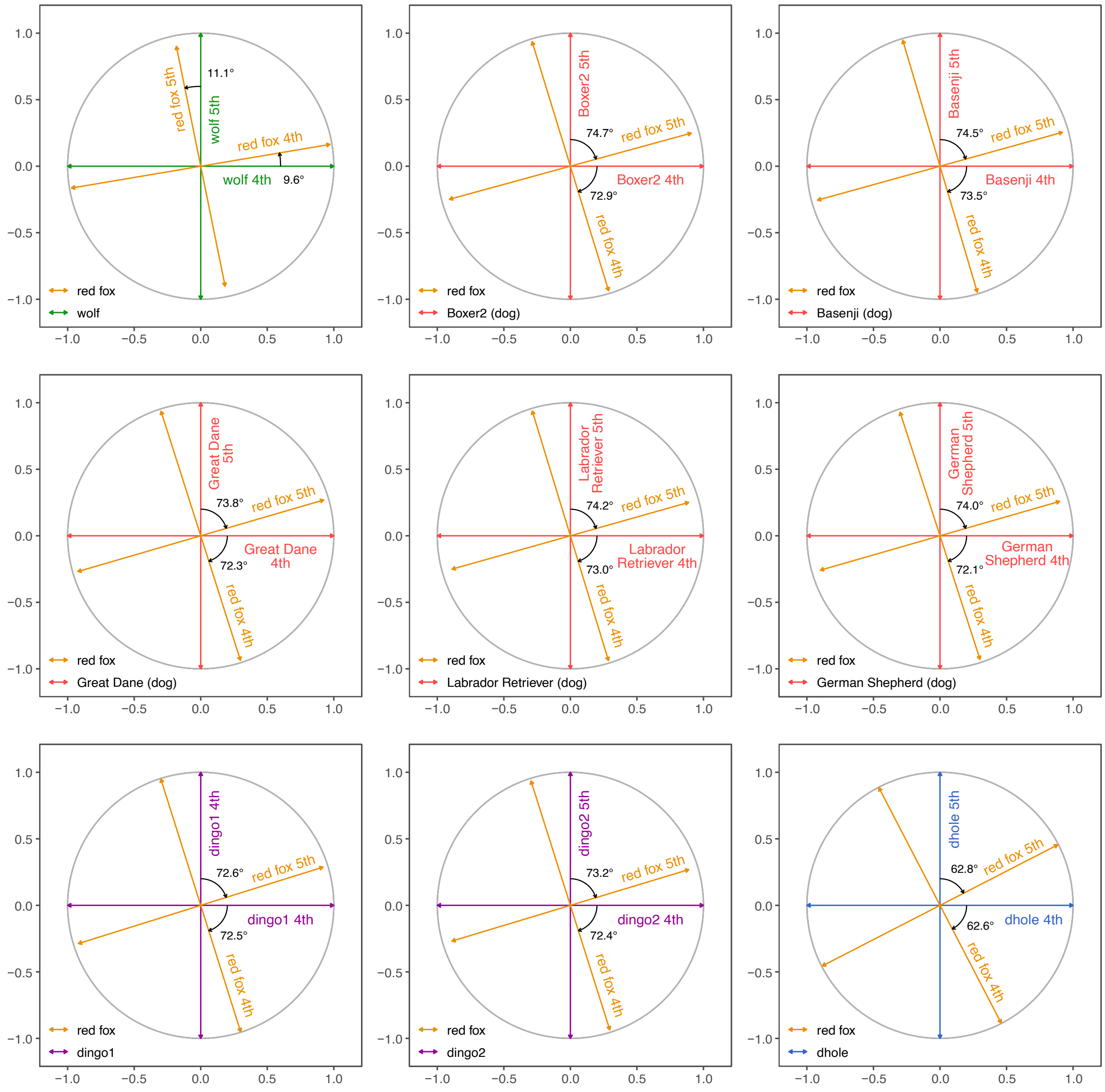

Fig. S7.

Rotations between the fourth and fifth motif-eigenvectors from nine canids to red foxes. The nine canids include a wolf, five breeds of dogs, two dingoes, and a dhole. Nine canids have almost the identical 2-D eigenspaces as red foxes. The two eigen-directions of red foxes rotate at a relatively large angle (between 62° and 75°) from all nine canids except for wolves.

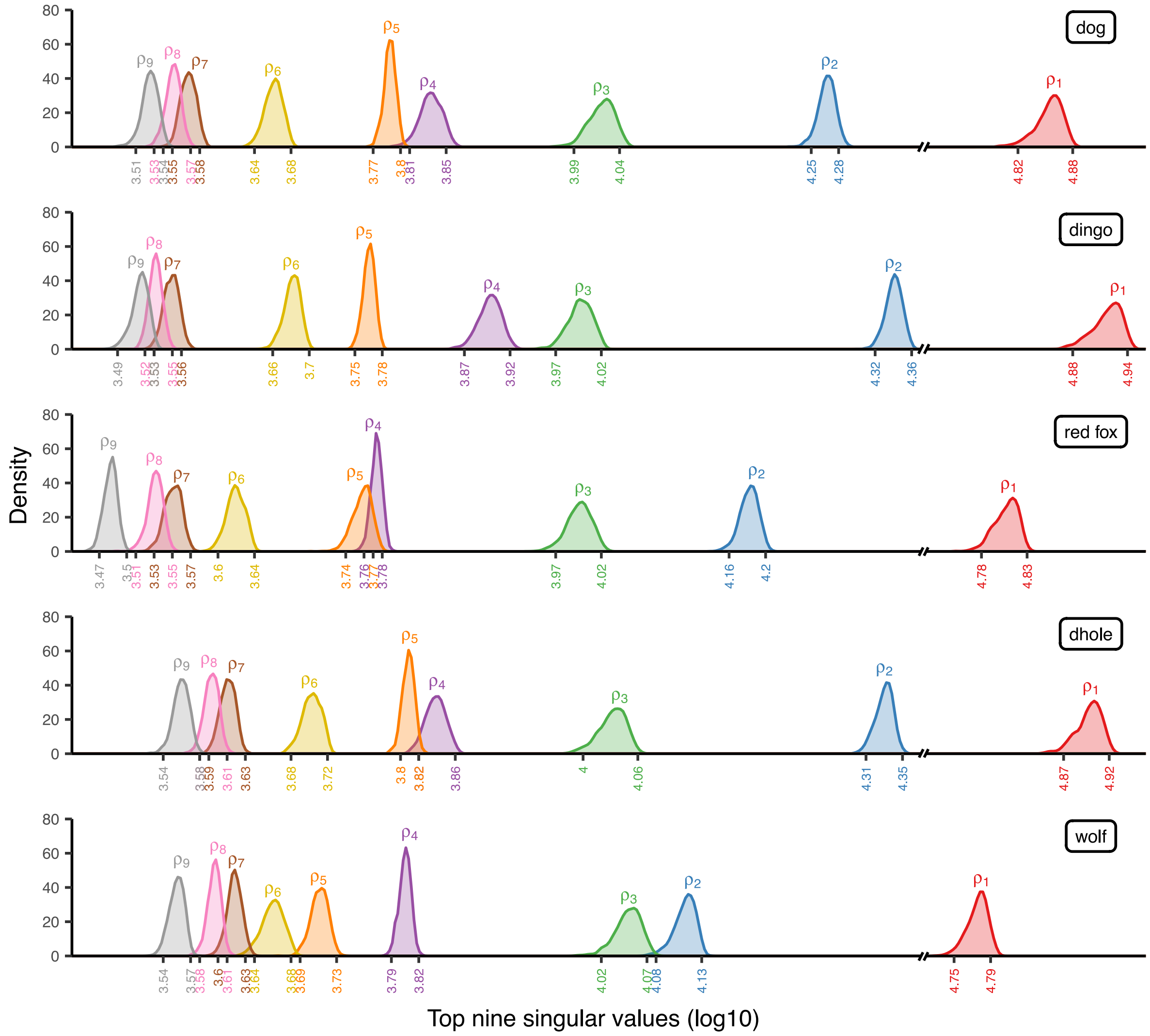

Fig. S8.

The sampling distributions of five canids' top nine singular values are shown by their densities from right to left in matched colors. The 2.5% and 97.5% quantiles are labeled along the x-axis. For dogs, red foxes, and dholes, the sampling distributions of the top three and the sixth singular values are well separated from adjacent ones while the fourth and fifth overlap by a portion. The red fox exhibits the largest overlapping proportion, indicating that the red fox is closest to the degenerate point that characterizes the saltation. The overlap is also observed between the seventh and eighth singular values in all five canids, and between the eighth and the ninth ones in dogs and dingoes.

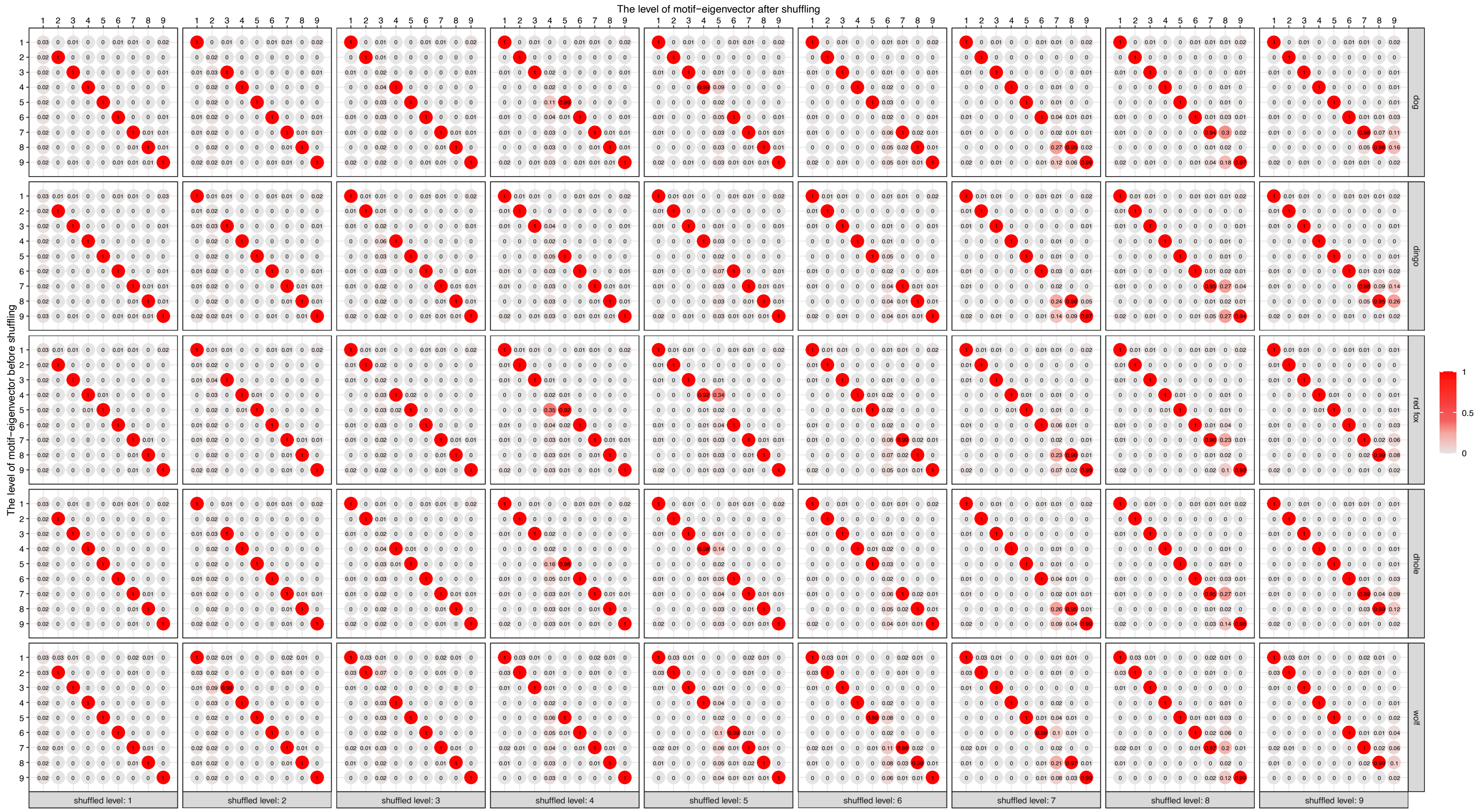

Fig. S9.

The mean absolute values of the Pearson correlation coefficients between the original motif-eigenvector and those obtained after random shuffling. The point in deeper color indicates a stronger correlation. The procedure of random shuffling is described in detail in the section "Stability analysis of CREF eigen-modules by random shuffling", Supplementary Text.

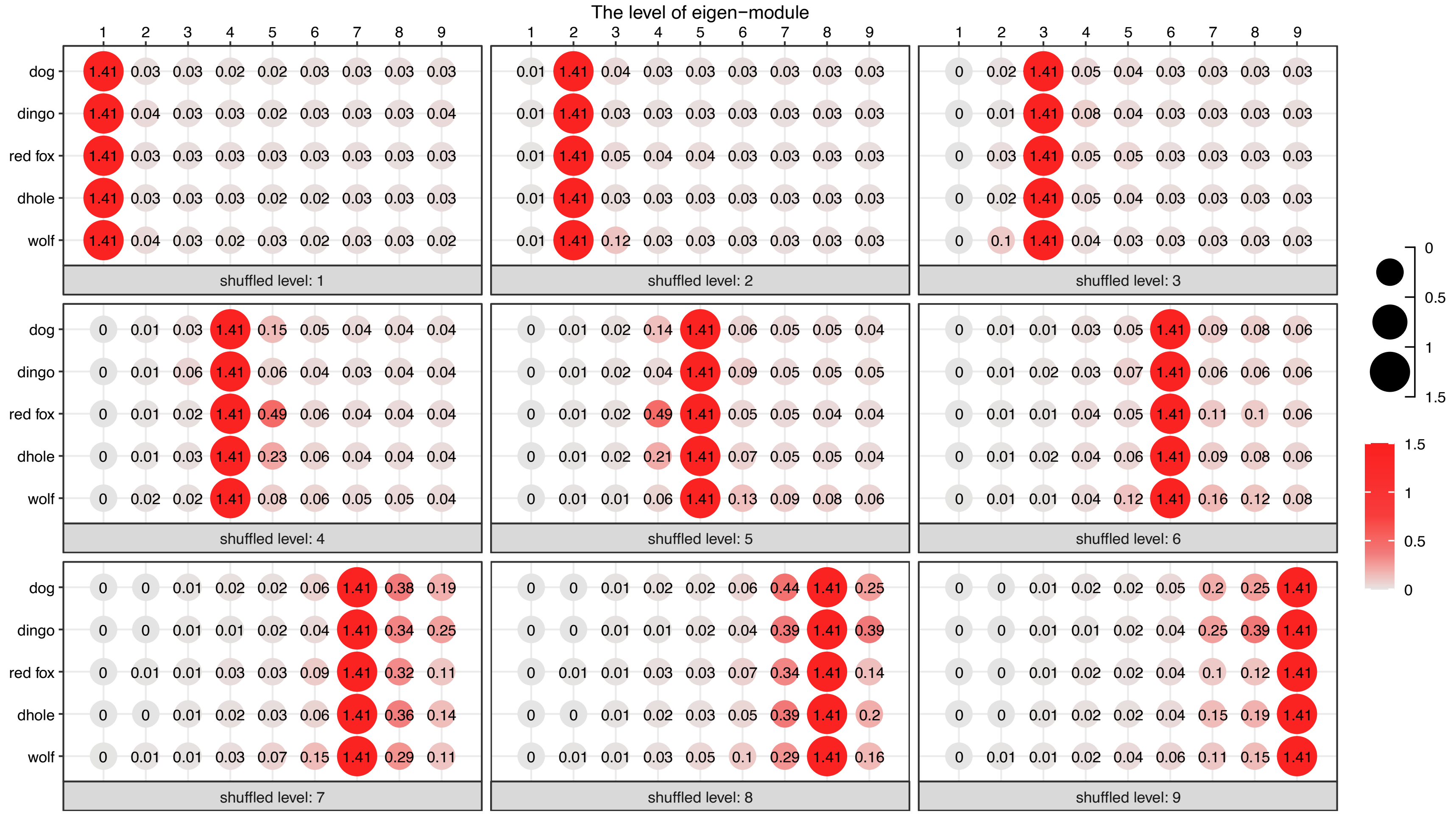

Fig. S10.

The mean Frobenius norms of the differences between the original eigen-module matrix and those obtained after random shuffling. The point in deeper color and larger size indicates a bigger difference. The procedure of random shuffling is described in detail in the section “Stability analysis of CREF eigen-modules by random shuffling”, Supplementary Text.

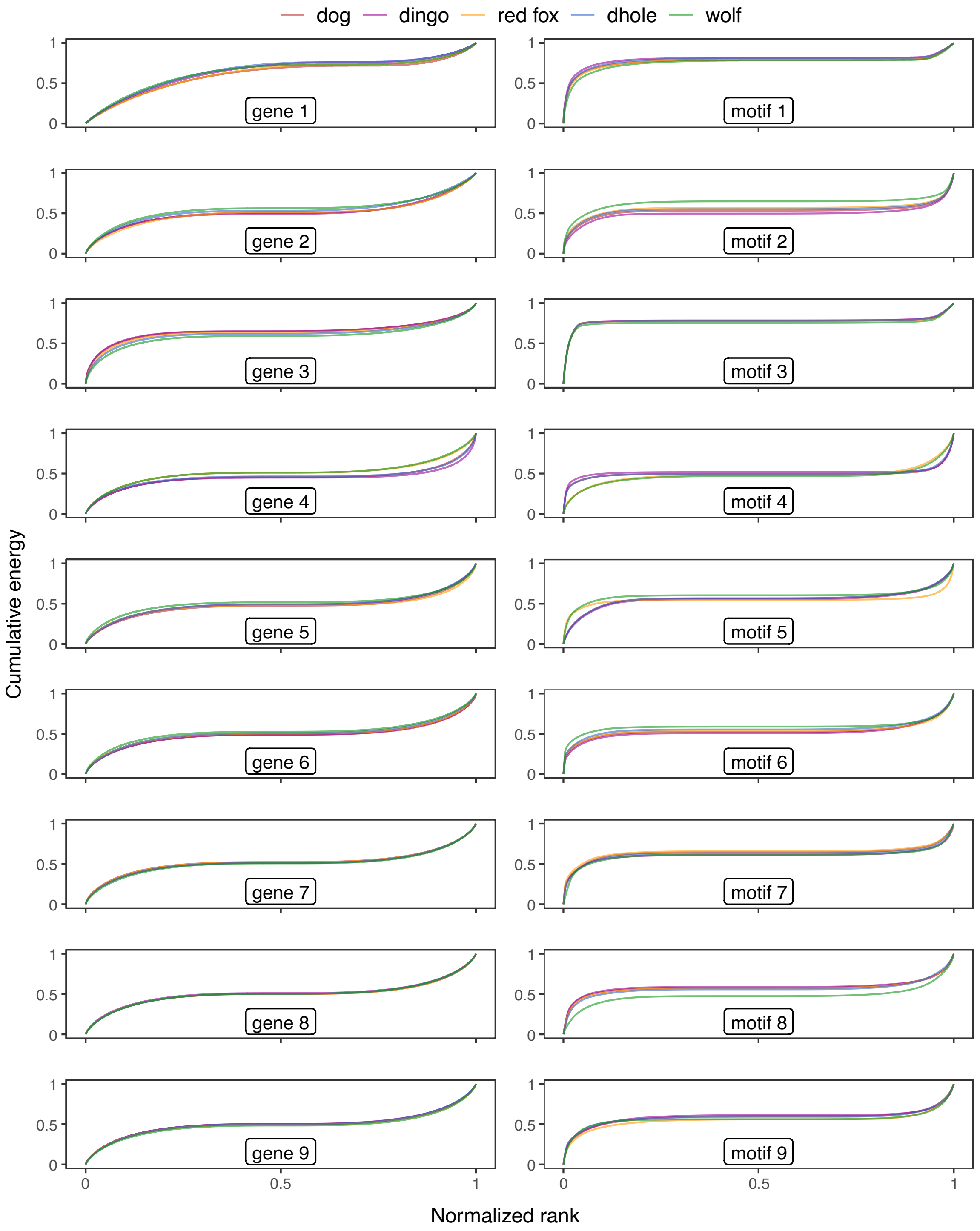

Fig. S11.

Cumulative distributions of the energy of the genes and motifs in the top nine polarized eigenvectors of five canids. The x-axis is the normalized rank of the gene loadings and motif loadings. The y-axis is their cumulative energy. The energy of motifs is more concentrated at the two poles than those of genes, indicating that in each CREF module, a small portion of *cis*-regulatory elements control the transcription of a relatively larger portion of genes.

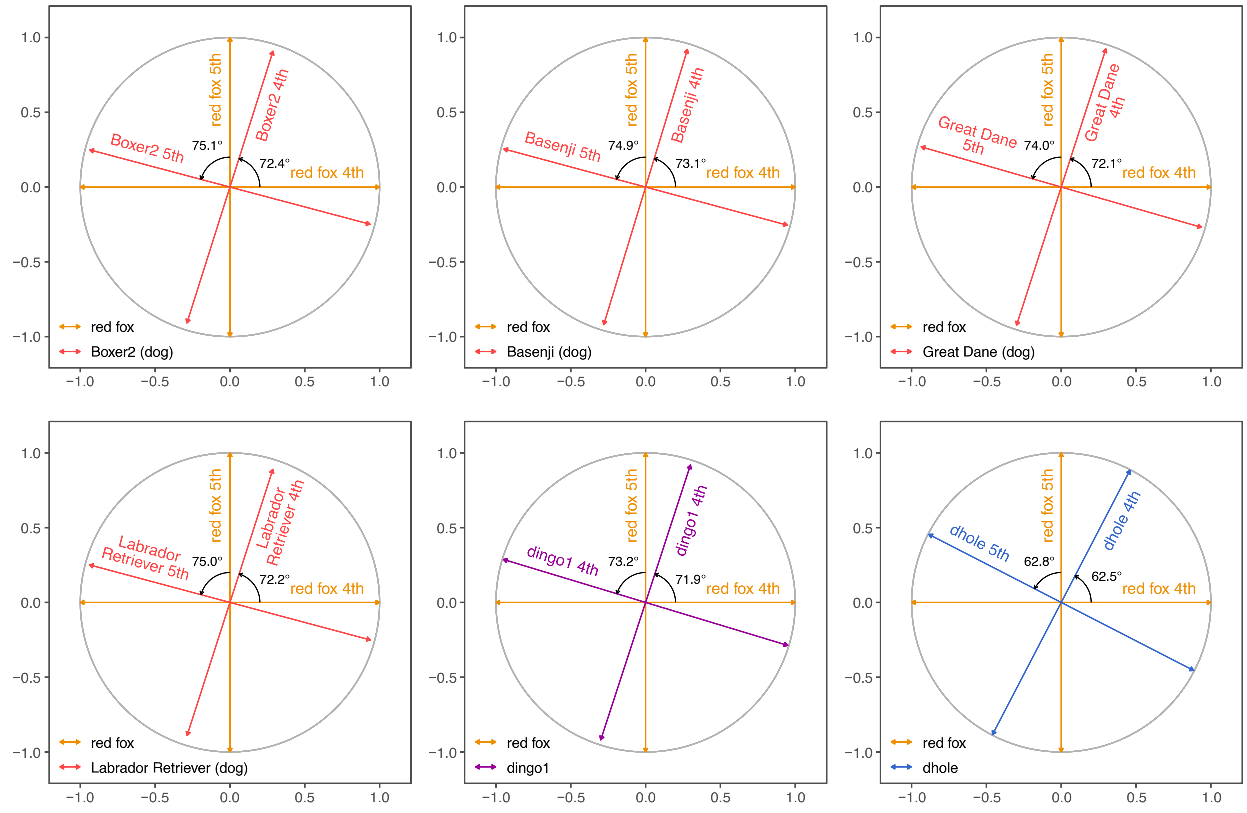

Fig. S12.

Rotations between the fourth and fifth motif-eigenvectors from red foxes to six canids. The six canids include four breeds of dogs, a dingo, and a dhole. While the six canids share almost identical 2-D eigenspaces as red foxes, their two eigen-directions rotate at a relatively large angle (between 62° and 75°) from red foxes.

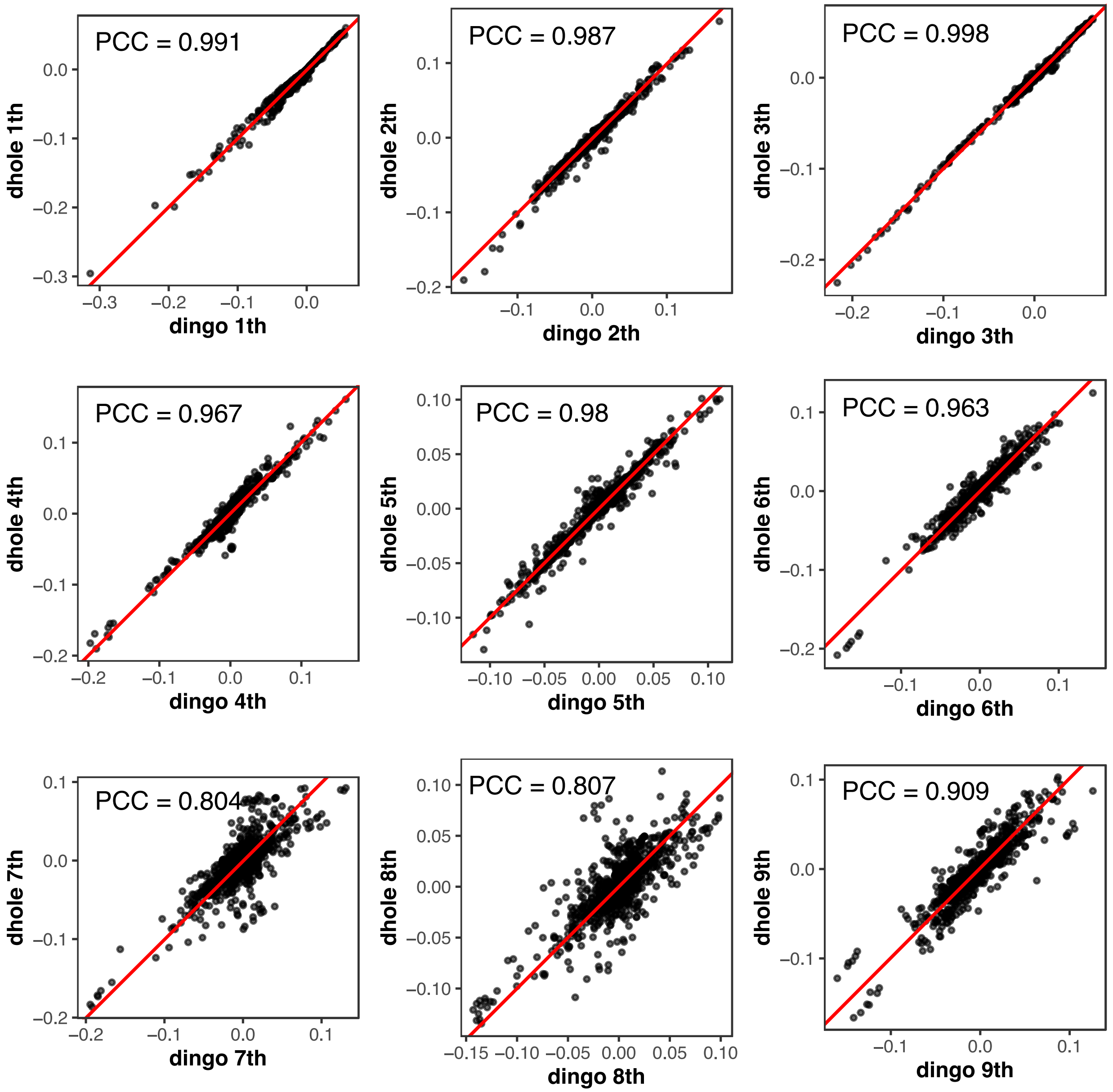

Fig. S13.

The scatter plots of dingoes' top nine motif eigenvector loadings versus dholes'. The straight lines are fitted by Deming regression. The top six PCCs (Pearson correlation coefficients) are greater than 0.96, the seventh and eighth ones are greater than 0.80, and the ninth is greater than 0.90. This indicates that the top six modules are highly conserved between dingoes and dholes.

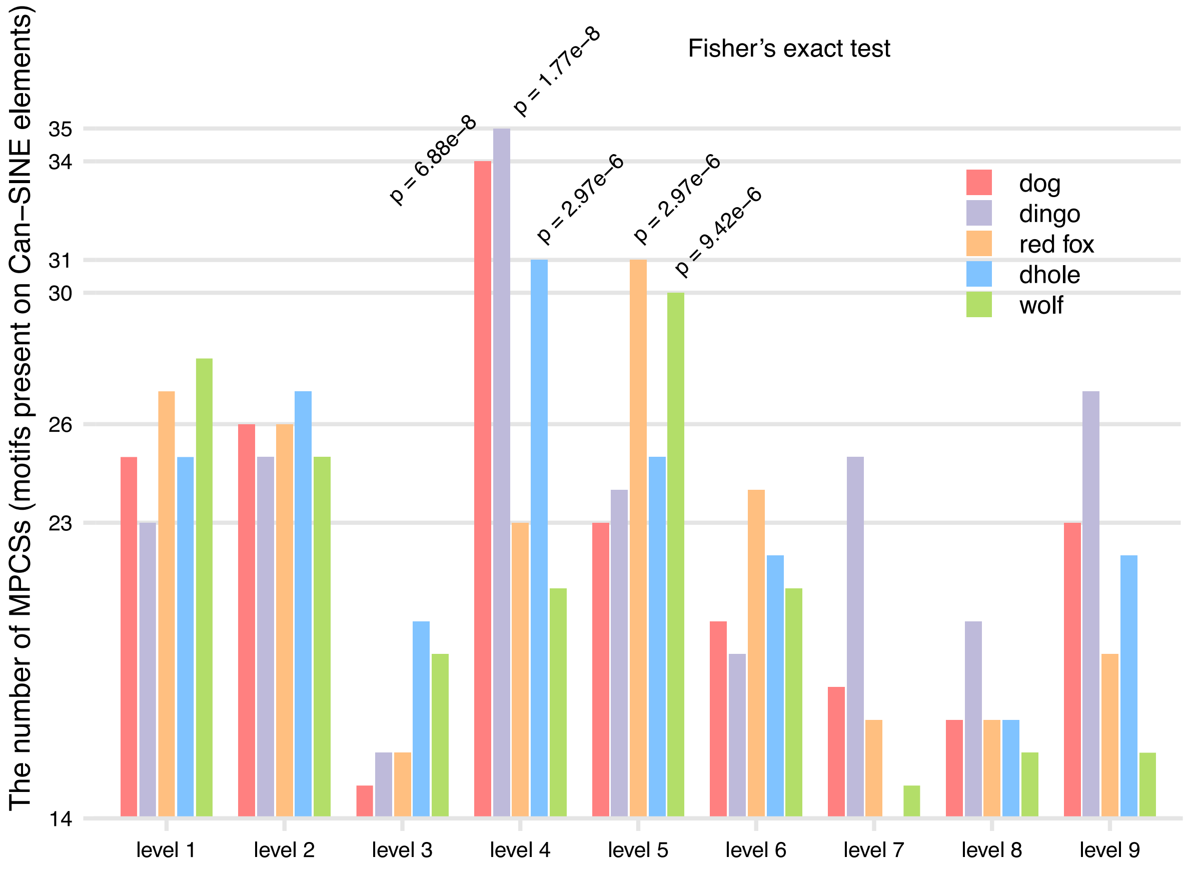

Fig. S14.

The number of MPCSs (motifs present on Can-SINE elements) in the top 100 motifs at each level of five canids. The analysis is based on the MPCSs output by the MATCH program with the minFP option. Fisher's exact test is performed to compare the odds of MPCS occurrences. The five significant p-values above the bar chart indicate that the MPCS occurrences increase significantly at level four in dogs, dingoes, and dholes, while they do so at level five in red foxes and wolves.

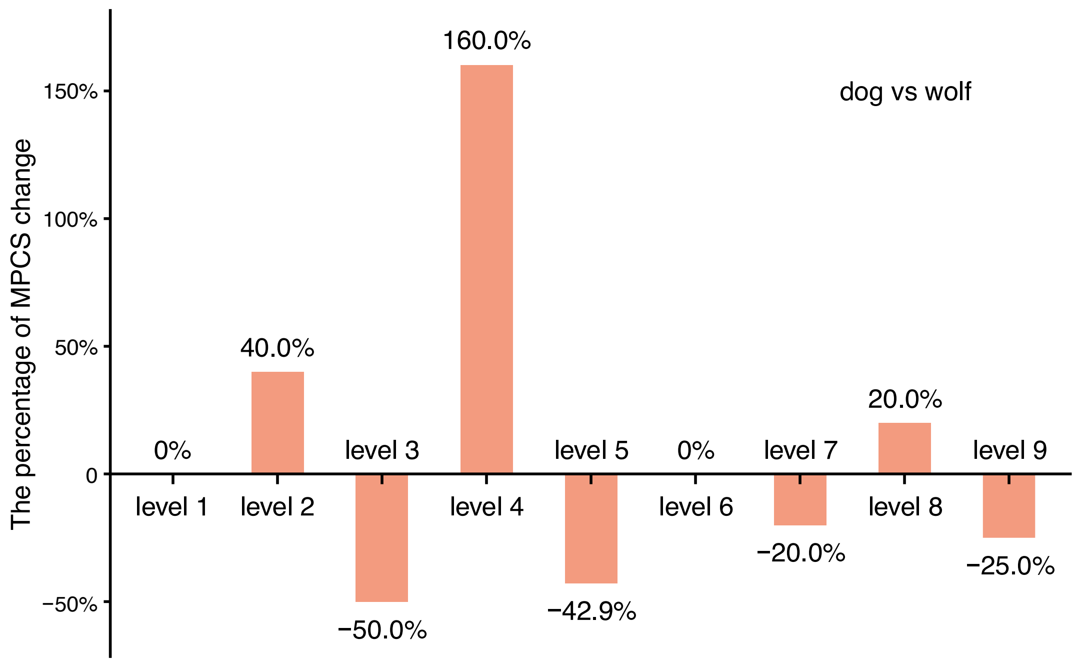

Fig. S15.

The relative change of MPCSs (motifs present on Can-SINE elements) in percentages at each level from wolf to dog. The analysis is based on the MPCSs output by the MATCH program with the minSUM option. The number of MPCSs increases most prominently at level four by 160.0%.

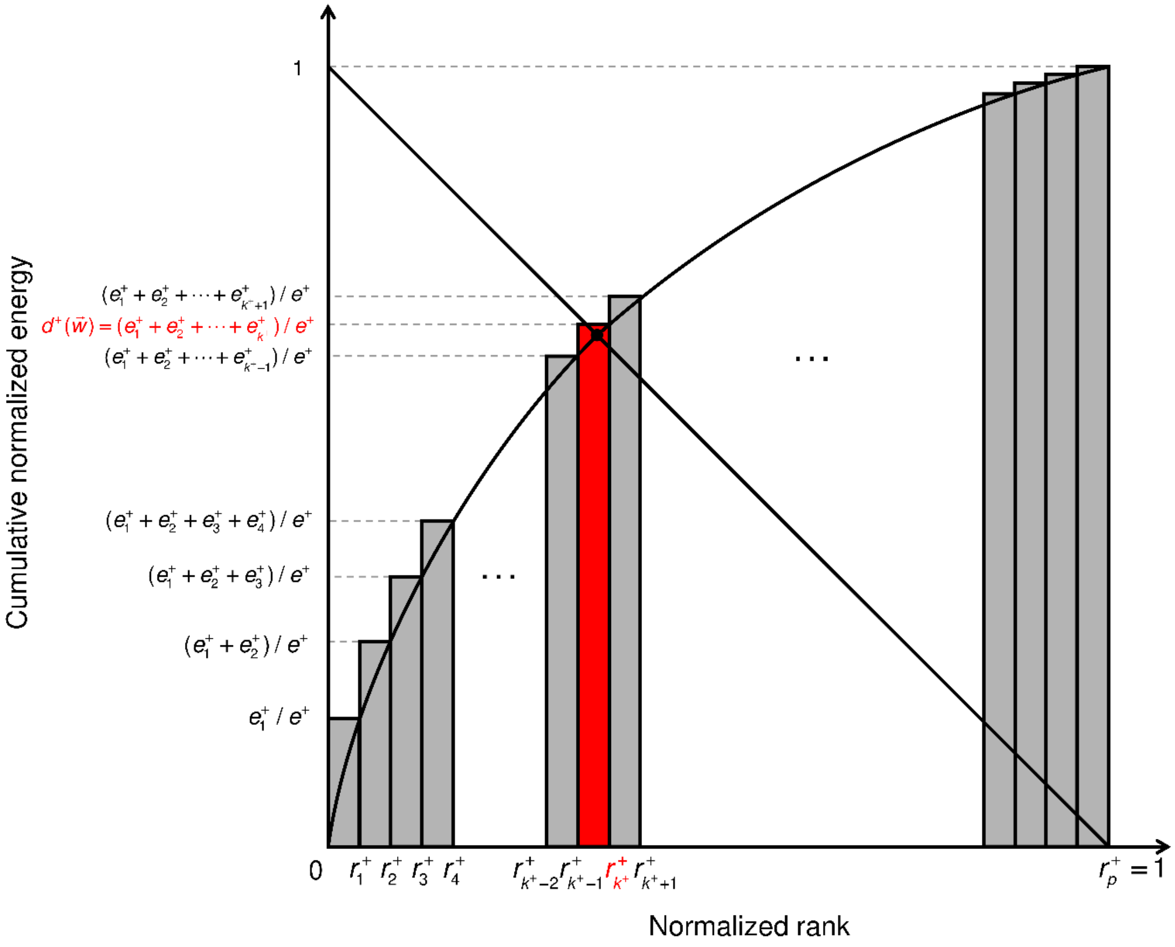

Fig. S16.

An intuitive description of the definition of the positive end polarization degree $d^+(\vec{w})$ of the polarized eigenvector $\vec{w}$. The mathematical definition is given in the section "Polarization degree of eigenvectors", Supplementary Text. The x-axis is the normalized rank of the loadings, where the notation $r_i^+ = \frac{i}{p}, i = 1,2,\cdots,p$. The y-axis is their cumulative normalized energy. From left to right, the gray rectangles with increasing height show the gradual accumulation of normalized energy. The concave curve successively connecting the right top of each rectangle outlines the growth trend of the cumulative normalized energy. The straight line that intersects with the curve has a slope of -1. The positive end polarization degree of $\vec{w}$ is the height of the first rectangle that intersects with the straight line from left to right. The rectangle is indicated in red. It is approximately equal to the y-ordinate of the intersection of the curve and the straight line.

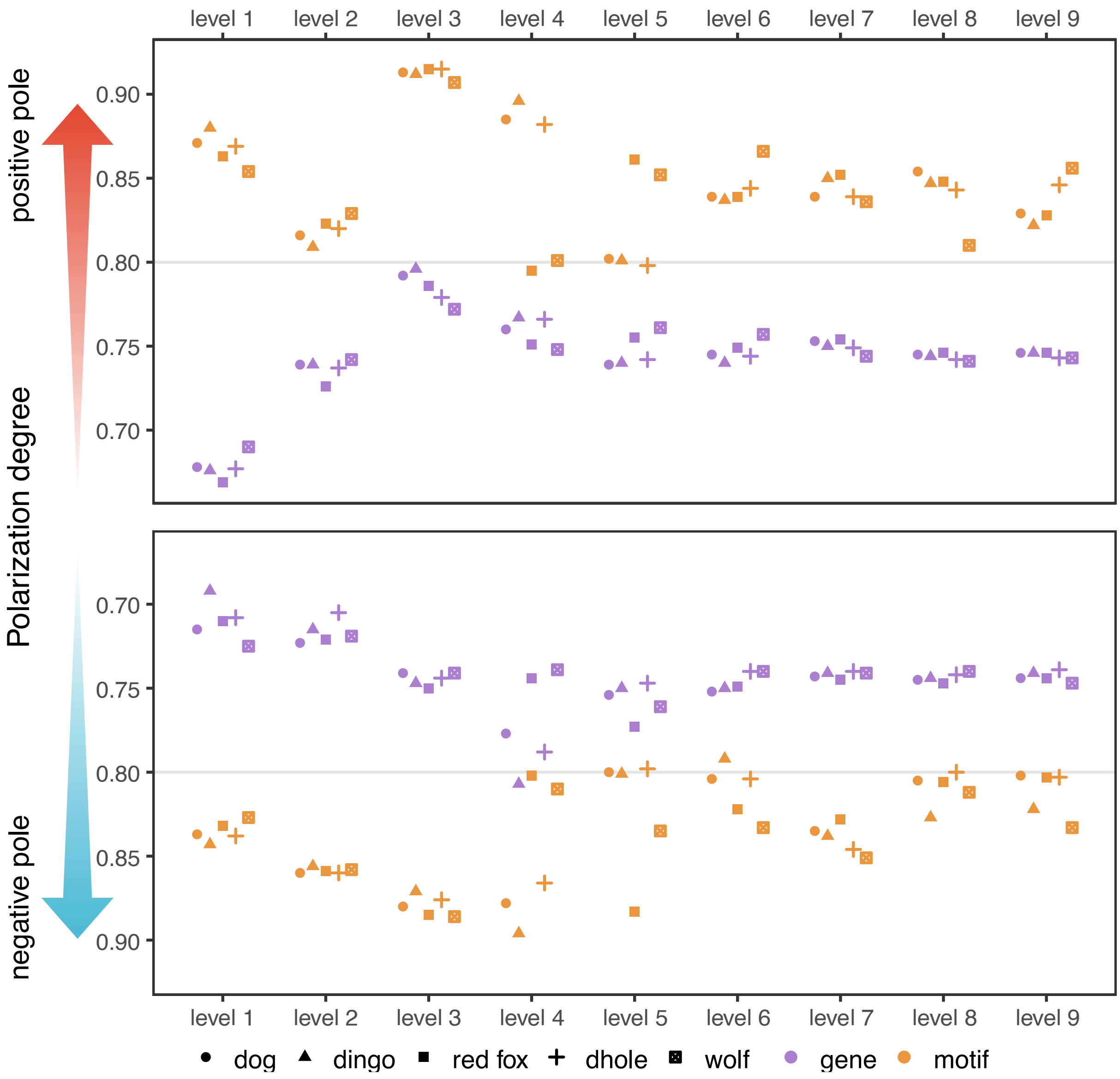

Fig. S17.

The scatter plots of the polarization degree of gene- and motif-eigenvectors. The upper part of the plot shows the positive pole, and the lower part shows the negative pole. Five canids are represented by different shapes of points. The purple points representing genes and the orange points representing motifs are well separated by the horizontal line at 0.80.

Table S1.

The top nine singular values of the CREF matrices, their percentages, and the cumulative percentages.

	Dog (Boxer1)			Dog (Boxer2)			Dog (Basenji)		
	Value ($\times 10^3$)	Percentage (%)	Cumulative percentage (%)	Value ($\times 10^3$)	Percentage (%)	Cumulative percentage (%)	Value ($\times 10^3$)	Percentage (%)	Cumulative percentage (%)
ρ_1	80.11	27.25	27.25	87.32	29.05	29.05	89.05	29.30	29.30
ρ_2	20.73	7.05	34.31	22.52	7.49	36.55	23.58	7.76	37.05
ρ_3	11.72	3.99	38.29	11.23	3.74	40.28	11.38	3.75	40.80
ρ_4	7.62	2.59	40.88	9.54	3.17	43.46	9.27	3.05	43.85
ρ_5	6.88	2.34	43.22	6.65	2.21	45.67	6.75	2.22	46.07
ρ_6	5.12	1.74	44.96	5.31	1.77	47.44	5.41	1.78	47.85
ρ_7	4.10	1.39	46.36	4.02	1.34	48.77	3.99	1.31	49.16
ρ_8	3.99	1.36	47.71	3.79	1.26	50.03	3.84	1.26	50.42
ρ_9	3.77	1.28	49.00	3.75	1.25	51.28	3.75	1.24	51.66

Table S1 (continued).
The top nine singular values of the CREF matrices, their percentages, and the cumulative percentages.

	Dog (Great Dane)			Dog (Labrador Retriever)			Dog (German Shepherd)		
	Value ($\times 10^3$)	Percentage (%)	Cumulative percentage (%)	Value ($\times 10^3$)	Percentage (%)	Cumulative percentage (%)	Value ($\times 10^3$)	Percentage (%)	Cumulative percentage (%)
ρ_1	90.23	29.38	29.38	91.76	28.96	28.96	98.90	29.81	29.81
ρ_2	23.75	7.73	37.11	23.76	7.50	36.46	24.86	7.49	37.30
ρ_3	11.36	3.70	40.81	12.41	3.92	40.38	11.98	3.61	40.91
ρ_4	9.24	3.01	43.82	10.62	3.35	43.73	10.69	3.22	44.13
ρ_5	6.88	2.24	46.06	7.00	2.21	45.94	7.25	2.19	46.32
ρ_6	5.42	1.76	47.82	5.64	1.78	47.72	5.93	1.79	48.11
ρ_7	4.07	1.32	49.15	4.23	1.33	49.05	4.42	1.33	49.44
ρ_8	3.90	1.27	50.42	3.97	1.25	50.31	4.17	1.26	50.70
ρ_9	3.81	1.24	51.66	3.92	1.24	51.55	4.15	1.25	51.95

Table S1 (continued).
The top nine singular values of the CREF matrices, their percentages, and the cumulative percentages.

	Dingo (dingo1)			Dingo (dingo2)			Red fox		
	Value ($\times10^3$)	Percentage (%)	Cumulative percentage (%)	Value ($\times10^3$)	Percentage (%)	Cumulative percentage (%)	Value ($\times10^3$)	Percentage (%)	Cumulative percentage (%)
ρ_1	92.72	30.43	30.43	91.61	29.07	29.07	71.75	26.58	26.58
ρ_2	24.53	8.05	38.48	23.42	7.43	36.50	17.02	6.31	32.89
ρ_3	11.11	3.64	42.12	11.37	3.61	40.11	11.06	4.10	36.99
ρ_4	8.84	2.90	45.02	10.32	3.27	43.38	6.59	2.44	39.43
ρ_5	6.51	2.14	47.16	6.98	2.21	45.59	6.46	2.39	41.82
ρ_6	5.36	1.76	48.92	5.57	1.77	47.36	4.64	1.72	43.54
ρ_7	3.92	1.29	50.20	4.17	1.32	48.68	3.96	1.47	45.00
ρ_8	3.79	1.24	51.45	3.99	1.27	49.95	3.81	1.41	46.42
ρ_9	3.68	1.21	52.66	3.92	1.24	51.19	3.39	1.25	47.67

Table S1 (continued).
The top nine singular values of the CREF matrices, their percentages, and the cumulative percentages.

	Dhole			Wolf		
	Value ($\times 10^3$)	Percentage (%)	Cumulative percentage (%)	Value ($\times 10^3$)	Percentage (%)	Cumulative percentage (%)
ρ_1	88.50	27.64	27.64	66.68	23.34	23.34
ρ_2	23.95	7.48	35.12	14.45	5.06	28.39
ρ_3	12.06	3.77	38.89	12.56	4.39	32.79
ρ_4	7.70	2.40	41.30	7.13	2.49	35.28
ρ_5	7.22	2.25	43.55	5.73	2.01	37.29
ρ_6	5.64	1.76	45.31	5.10	1.79	39.07
ρ_7	4.54	1.42	46.73	4.63	1.62	40.69
ρ_8	4.40	1.37	48.10	4.43	1.55	42.24
ρ_9	4.08	1.27	49.37	4.01	1.40	43.65

Table S2.

The relative distance between adjacent singular values of the CREF matrices.

	Relative distance (%)										
	Dog (Boxer1)	Dog (Boxer2)	Dog (Basenji)	Dog (Great Dane)	Dog (Labrador Retriever)	Dog (German Shepherd)	Dingo (dingo1)	Dingo (dingo2)	Red fox	Dhole	Wolf
ρ_1 - ρ_2	74.12	74.21	73.52	73.68	74.11	74.86	73.55	74.44	76.28	72.93	78.33
ρ_2 - ρ_3	43.49	50.14	51.72	52.16	47.76	51.79	54.71	51.47	35.00	49.67	13.10
ρ_3 - ρ_4	34.98	15.09	18.58	18.69	14.44	10.83	20.41	9.23	40.48	36.14	43.23
ρ_4 - ρ_5	9.72	30.25	27.22	25.55	34.07	32.13	26.33	32.35	1.91	6.28	19.55
ρ_5 - ρ_6	25.63	20.16	19.75	21.25	19.53	18.22	17.66	20.24	28.23	21.89	11.06
ρ_6 - ρ_7	19.85	24.32	26.31	24.95	25.03	25.42	26.86	25.08	14.64	19.50	9.16
ρ_7 - ρ_8	2.72	5.73	3.79	4.13	5.94	5.80	3.36	4.39	3.71	3.08	4.46
ρ_8 - ρ_9	5.48	0.97	2.17	2.32	1.32	0.48	2.94	1.63	11.16	7.32	9.35

Table S3.

The polarization degree of the top nine gene- and motif-eigenvectors of five canids. The mathematical definition of polarization degree is given in the section "Polarization degree of eigenvectors", Supplementary Text. For each eigenvector, the polarization degree of the positive pole and negative pole is shown respectively. The range of the polarization degree of gene-eigenvectors is from 0.669 to 0.807. The range of the polarization degree of motif-eigenvectors is from 0.792 to 0.915. Compared with genes, motifs exhibit a higher concentration of energy at the poles. Pos, the polarization degree of the positive pole; Neg, the polarization degree of the negative pole.

		Gene-eigenvector		Motif-eigenvector	
**Species**	**Level**	**Pos**	**Neg**	**Pos**	**Neg**
dog	1	0.678	0.715	0.871	0.837
dingo	1	0.676	0.692	0.880	0.843
red fox	1	0.669	0.710	0.863	0.832
dhole	1	0.677	0.708	0.869	0.838
wolf	1	0.690	0.725	0.854	0.827
dog	2	0.739	0.723	0.816	0.860
dingo	2	0.739	0.715	0.809	0.856
red fox	2	0.726	0.721	0.823	0.859
dhole	2	0.737	0.705	0.820	0.860
wolf	2	0.742	0.719	0.829	0.858
dog	3	0.792	0.741	0.913	0.880
dingo	3	0.796	0.747	0.912	0.871
red fox	3	0.786	0.750	0.915	0.885
dhole	3	0.779	0.744	0.915	0.876
wolf	3	0.772	0.741	0.907	0.886
dog	4	0.760	0.777	0.885	0.878
dingo	4	0.767	0.807	0.896	0.896
red fox	4	0.751	0.744	0.795	0.802
dhole	4	0.766	0.788	0.882	0.866
wolf	4	0.748	0.739	0.801	0.810
dog	5	0.739	0.754	0.802	0.800
dingo	5	0.740	0.750	0.801	0.801
red fox	5	0.755	0.773	0.861	0.883
dhole	5	0.742	0.747	0.798	0.798
wolf	5	0.761	0.761	0.852	0.835
dog	6	0.745	0.752	0.839	0.804
dingo	6	0.740	0.750	0.837	0.792
red fox	6	0.749	0.749	0.839	0.822
dhole	6	0.744	0.740	0.844	0.804
wolf	6	0.757	0.740	0.866	0.833

Table S3 (continued).

The polarization degree of the top nine gene- and motif-eigenvectors of five canids. The mathematical definition of polarization degree is given in the section "Polarization degree of eigenvectors", Supplementary Text. For each eigenvector, the polarization degree of the positive pole and negative pole is shown respectively. The range of the polarization degree of gene-eigenvectors is from 0.669 to 0.807. The range of the polarization degree of motif-eigenvectors is from 0.792 to 0.915. Compared with genes, motifs exhibit a higher concentration of energy at the poles. Pos, the polarization degree of the positive pole; Neg, the polarization degree of the negative pole.

		**Gene-eigenvector**		**Motif-eigenvector**	
**Species**	**Level**	**Pos**	**Neg**	**Pos**	**Neg**
dog	7	0.753	0.743	0.839	0.835
dingo	7	0.750	0.741	0.850	0.838
red fox	7	0.754	0.745	0.852	0.828
dhole	7	0.749	0.740	0.839	0.846
wolf	7	0.744	0.741	0.836	0.851
dog	8	0.745	0.745	0.854	0.805
dingo	8	0.744	0.744	0.847	0.827
red fox	8	0.746	0.747	0.848	0.806
dhole	8	0.742	0.742	0.843	0.800
wolf	8	0.741	0.740	0.810	0.812
dog	9	0.746	0.744	0.829	0.802
dingo	9	0.746	0.741	0.822	0.822
red fox	9	0.746	0.744	0.828	0.803
dhole	9	0.743	0.739	0.846	0.803
wolf	9	0.743	0.747	0.856	0.833

Table S4.

The significant long-term memory-related gene subsets enriched at the positive pole of the dog fourth polarized gene-eigenvector.

Category	Description	Source	P-value
Long-term memory	learning or memory	GO BP	2.76E-02
	memory	GO BP	3.80E-02
	long-term memory	GO BP	7.96E-02
Neurotransmission	glycinergic synapse	GO CC	5.96E-03
	glutamatergic synapse	KEGG	1.50E-03
	cholinergic synapse	KEGG	4.22E-03
	serotonergic synapse	KEGG	1.91E-02
	GABAergic synapse	KEGG	1.13E-02
	dopaminergic synapse	KEGG	5.14E-04
	hippocampal mossy fiber to CA3 synapse	GO CC	1.07E-02
	membrane depolarization	GO BP	5.22E-02
	membrane hyperpolarization	GO BP	1.28E-02
	membrane repolarization	GO BP	4.76E-02
	excitatory postsynaptic potential	GO BP	1.84E-04
	inhibitory postsynaptic potential	GO BP	3.02E-02
	transmission across chemical synapses	Reactome	2.16E-02
	positive regulation of synaptic transmission	GO BP	9.44E-02
	regulation of synaptic transmission, glutamatergic	GO BP	3.10E-02
	neuromuscular synaptic transmission	GO BP	6.35E-03
	neurotransmitter receptors and postsynaptic signal transmission	Reactome	6.58E-02
	postsynapse	GO CC	1.29E-02
	synaptic vesicle	GO CC	3.70E-02
	postsynaptic membrane	GO CC	3.30E-02
	synaptic vesicle priming	GO BP	1.84E-02
	synaptic vesicle maturation	GO BP	1.90E-03
	regulation of synaptic vesicle fusion to presynaptic active zone membrane	GO BP	1.67E-02
	synaptic vesicle transport	GO BP	2.85E-02
	dendritic spine	GO CC	6.41E-04
	dendrite	GO CC	2.84E-02
Synaptic plasticity	regulation of long-term neuronal synaptic plasticity	GO BP	1.67E-03
	long-term synaptic potentiation	GO BP	2.63E-02
	negative regulation of long-term synaptic potentiation	GO BP	3.15E-03
	negative regulation of long-term synaptic depression	GO BP	1.97E-02
	long-term potentiation	KEGG	1.30E-03
	long-term depression	KEGG	3.96E-04

Table S4 (continued).

The significant long-term memory-related gene subsets enriched at the positive pole of the dog fourth polarized gene-eigenvector.

Category	Description	Source	P-value
LTP induction	regulation of AMPA receptor activity	GO BP	1.41E-04
	AMPA glutamate receptor complex	GO CC	5.62E-02
	activation of AMPA receptors	Reactome	1.35E-02
	ionotropic glutamate receptor signaling pathway	GO BP	3.05E-04
	ionotropic glutamate receptor activity	GO MF	1.13E-03
	glutamate binding	GO MF	4.20E-02
	voltage-gated calcium channel complex	GO CC	5.95E-03
	voltage-gated calcium channel activity	GO MF	2.90E-02
	calcium ion transmembrane transport	GO BP	2.84E-03
	regulation of postsynaptic cytosolic calcium ion concentration	GO BP	6.32E-03
Other LTP mechanisms	cellular response to calcium ion	GO BP	5.01E-02
	calcium ion binding	GO MF	1.89E-04
	regulation of protein phosphorylation	GO BP	9.10E-02
	positive regulation of protein phosphorylation	GO BP	5.93E-02
	protein autophosphorylation	GO BP	4.19E-02
	positive regulation of protein autophosphorylation	GO BP	4.56E-02
	peptidyl-threonine phosphorylation	GO BP	5.92E-02
	peptidyl-tyrosine phosphorylation	GO BP	8.64E-04
	positive regulation of peptidyl-serine phosphorylation	GO BP	1.18E-02
	positive regulation of peptidyl-tyrosine phosphorylation	GO BP	2.41E-02
	peptidyl-serine autophosphorylation	GO BP	7.21E-02
	cAMP biosynthetic process	GO BP	8.00E-02
	response to cAMP	GO BP	7.32E-02
	cellular response to cAMP	GO BP	2.10E-02
	cAMP signaling pathway	KEGG	2.84E-04
	cGMP-PKG signaling pathway	KEGG	8.34E-02
	protein-protein interactions at synapses	Reactome	2.31E-02

Table S5.

The details of the significant myelination-related gene subsets enriched at the poles of the human fourth polarized gene-eigenvector.

Category	Description	Source	P-value
Myelination	negative regulation of myelination	GO BP	3.40E-02
	positive regulation of myelination	GO BP	2.47E-02
	myelin assembly	GO BP	3.20E-02
	myelination	GO BP	4.62E-02
Myelin-forming cell	regulation of oligodendrocyte differentiation	GO BP	5.22E-02
	Schwann cell differentiation	GO BP	4.31E-02
	Schwann cell development	GO BP	7.74E-03
Myelin lipid metabolism	phospholipid biosynthetic process	GO BP	1.90E-02
	phospholipid homeostasis	GO BP	3.40E-02
	positive regulation of phospholipid transport	GO BP	6.95E-02
	phospholipid transporter activity	GO MF	1.57E-02
	phospholipid metabolism	Reactome	3.70E-02
	glycerophospholipid biosynthetic process	GO BP	5.31E-02
	glycerophospholipid metabolism	KEGG	6.81E-02
	glycerophospholipid biosynthesis	Reactome	2.90E-02
	glycosphingolipid metabolic process	GO BP	2.97E-02
	glycosphingolipid metabolism	Reactome	6.66E-02
Cholesterol metabolism	cholesterol biosynthetic process	GO BP	8.19E-02
	cholesterol transport	GO BP	6.71E-02
	negative regulation of cholesterol transport	GO BP	7.27E-02
	regulation of intracellular cholesterol transport	GO BP	6.71E-02
	cholesterol homeostasis	GO BP	6.31E-02
	regulation of cholesterol biosynthetic process	GO BP	2.77E-02
	positive regulation of cholesterol metabolic process	GO BP	7.25E-02
	regulation of cholesterol biosynthesis by SREBP (SREBF)	Reactome	5.95E-02
	cholesterol biosynthesis	Reactome	2.27E-02
Fatty acid metabolism	long-chain fatty acid metabolic process	GO BP	8.55E-02
	fatty acid metabolic process	GO BP	7.20E-02
	fatty acid elongation	GO BP	6.74E-02
	very long-chain fatty acid biosynthetic process	GO BP	8.04E-02
	negative regulation of fatty acid biosynthetic process	GO BP	2.65E-02
	positive regulation of fatty acid biosynthetic process	GO BP	4.94E-03
	fatty acid homeostasis	GO BP	2.57E-02
	fatty acid biosynthesis	KEGG	6.68E-03
	fatty acid elongation	KEGG	2.90E-02
	fatty acid metabolism	KEGG	6.65E-02
	fatty acids	Reactome	3.82E-02
	fatty acid metabolism	Reactome	6.21E-02

Table S6.

The details of the significant myelination-related gene subsets enriched at the poles of the dog fourth polarized gene-eigenvector.

Category	Description	Source	P-value
Myelination	central nervous system myelination	GO BP	7.72E-04
	myelination in peripheral nervous system	GO BP	1.04E-01
	regulation of myelination	GO BP	4.53E-02
	negative regulation of myelination	GO BP	8.25E-02
	myelin sheath abaxonal region	GO CC	5.83E-02
	myelin sheath adaxonal region	GO CC	5.04E-02
	myelin sheath	GO CC	8.08E-03
Myelin-forming cell	oligodendrocyte development	GO BP	9.15E-02
	regulation of oligodendrocyte differentiation	GO BP	9.85E-02
	Schwann cell development	GO BP	1.89E-02
Myelin lipid metabolism	phospholipid metabolic process	GO BP	9.98E-02
	phospholipid transport	GO BP	6.85E-02
	glycerophospholipid metabolic process	GO BP	1.76E-02
	phosphatidylcholine biosynthetic process	GO BP	8.20E-02
	sphingolipid metabolism	Reactome	7.65E-02
	glycosphingolipid biosynthesis - ganglio series	KEGG	1.50E-02
Cholesterol metabolism	cholesterol biosynthetic process	GO BP	6.23E-02
	cholesterol metabolic process	GO BP	1.09E-02
	regulation of intracellular cholesterol transport	GO BP	1.01E-02
	regulation of cholesterol biosynthetic process	GO BP	5.07E-02
	negative regulation of cholesterol biosynthetic process	GO BP	3.50E-02
	cholesterol metabolism	KEGG	4.61E-02
Fatty acid metabolism	long-chain fatty acid metabolic process	GO BP	2.50E-02
	fatty acid elongation	GO BP	5.21E-02
	negative regulation of fatty acid biosynthetic process	GO BP	5.87E-02
	negative regulation of fatty acid metabolic process	GO BP	1.01E-01
	fatty acid homeostasis	GO BP	4.23E-02
	fatty acid biosynthesis	KEGG	1.26E-02

Table S7.

The details of the significant cochlea development-related gene subsets enriched at the poles of the human fourth polarized gene-eigenvector.

Description	Source	P-value
cochlea morphogenesis	GO BP	8.24E-05
inner ear morphogenesis	GO BP	4.75E-02
inner ear development	GO BP	1.52E-03
inner ear receptor cell differentiation	GO BP	8.95E-03
auditory receptor cell stereocilium organization	GO BP	7.63E-02
sensory perception of sound	GO BP	4.66E-03
detection of mechanical stimulus involved in sensory perception of sound	GO BP	5.83E-02

Table S8.

The details of the significant cochlea development-related gene subsets enriched at the poles of the dog fourth polarized gene-eigenvector.

Description	Source	P-value
cochlea development	GO BP	8.75E-02
inner ear receptor cell differentiation	GO BP	3.86E-02
inner ear receptor cell development	GO BP	6.33E-02
vestibulocochlear nerve formation	GO BP	8.83E-02
auditory receptor cell stereocilium organization	GO BP	3.18E-02
sensory perception of sound	GO BP	6.36E-02

Table S9.

Contingency table for the comparison of the occurrences of MPCSs and other motifs at the top of the motif-eigenvector. A total of 1403 motifs can be divided into four categories according to whether they are MPCSs and whether they occur in the top 100. The fixed marginal totals are based on the MPCSs output by the MATCH program with the minSUM option.

	Motifs in the top 100	Motifs not in the top 100	Total
MPCSs	n_{11}	n_{10}	97
Other motifs (not MPCSs)	n_{01}	n_{00}	1306
Total	200	1203	1403

Table S10.

The details of the comparison of the frequency of MPCSs and other motifs occurring in the top 100 at the two poles by one-sided Fisher's exact test. The analysis is based on the MPCSs output by the MATCH program with the minSUM option.

Fisher's exact test H_0: OR = 1 ↔ H_1: OR > 1									
			Number of motifs in the top 100	Number of motifs not in the top 100	P-value	Significance	Estimate of odds ratio	95% lower confidence bound
Level 4	dog	MPCSs	34	63	6.88E-08	***	3.70	2.47
		other motifs (not MPCSs)	166	1140				
	dingo	MPCSs	35	62	1.77E-08	***	3.89	2.61
		other motifs (not MPCSs)	165	1141				
	red fox	MPCSs	23	74	6.72E-03	**	1.98	1.26
		other motifs (not MPCSs)	177	1129				
	dhole	MPCSs	31	66	2.97E-06	***	3.16	2.08
		other motifs (not MPCSs)	169	1137				
	wolf	MPCSs	21	76	2.66E-02	*	1.74	1.08
		other motifs (not MPCSs)	179	1127				
Level 5	dog	MPCSs	23	74	6.72E-03	**	1.98	1.26
		other motifs (not MPCSs)	177	1129				
	dingo	MPCSs	24	73	3.10E-03	**	2.11	1.35
		other motifs (not MPCSs)	176	1130				
	red fox	MPCSs	31	66	2.97E-06	***	3.16	2.08
		other motifs (not MPCSs)	169	1137				
	dhole	MPCSs	25	72	1.35E-03	**	2.24	1.44
		other motifs (not MPCSs)	175	1131				
	wolf	MPCSs	30	67	9.42E-06	***	2.99	1.97
		other motifs (not MPCSs)	170	1136				
Significance codes: 0 '***' 0.001 '**' 0.01 '*' 0.05 '.' 0.1 ' ' 1									

Table S11.

The details of the comparison of the frequency of MPCSs and other motifs occurring among the top 100 at the two poles by one-sided Fisher's exact test. The analysis is based on the MPCSs output by the MATCH program with the minFP option.

Fisher's exact test<br>H_0: OR = 1 ↔ H_1: OR > 1								
			Number of motifs in the top 100	Number of motifs not in the top 100	P-value	Significance	Estimate of odds ratio	95% lower confidence bound
Level 4	dog	MPCSs	13	9	1.07E-06	***	9.20	4.10
		other motifs (not MPCSs)	187	1194				
	dingo	MPCSs	12	10	9.01E-06	***	7.60	3.40
		other motifs (not MPCSs)	188	1193				
	red fox	MPCSs	5	17	1.94E-01		1.79	0.63
		other motifs (not MPCSs)	195	1186				
	dhole	MPCSs	10	12	3.78E-04	***	5.21	2.30
		other motifs (not MPCSs)	190	1191				
	wolf	MPCSs	5	17	1.94E-01		1.79	0.63
		other motifs (not MPCSs)	195	1186				
Level 5	dog	MPCSs	4	18	3.85E-01		1.34	0.41
		other motifs (not MPCSs)	196	1185				
	dingo	MPCSs	6	16	8.05E-02	.	2.29	0.87
		other motifs (not MPCSs)	194	1187				
	red fox	MPCSs	10	12	3.78E-04	***	5.21	2.30
		other motifs (not MPCSs)	190	1191				
	dhole	MPCSs	4	18	3.85E-01		1.34	0.41
		other motifs (not MPCSs)	196	1185				
	wolf	MPCSs	7	15	2.76E-02	*	2.87	1.16
		other motifs (not MPCSs)	193	1188				
Significance codes: 0 '***' 0.001 '**' 0.01 '*' 0.05 '.' 0.1 ' ' 1								

Table S12.

The rank of 22 MPCSs in the fourth and fifth polarized motif-eigenvectors of five canids. MPCSs in the top 100 motifs at the two poles have been highlighted. Motifs present at the negative pole are marked by negative ranks.

			Level 4					Level 5				
Motif	TF	Consensus sequence	Dog	Dingo	Red fox	Dhole	Wolf	Dog	Dingo	Red fox	Dhole	Wolf
CACD_01	CACD	CCACRCCC	14	16	156	15	391	286	-410	13	246	33
E2F_03	E2F	TTTSGCGCGMNR	-55	-46	283	-63	232	-139	-167	-41	-139	-58
E2F_Q6_01	E2F	NKCGCGCSAAAN	-66	-55	325	-75	252	-156	-187	-54	-162	-70
MAZ_Q6	MAZ	GGGGAGGG	22	24	198	21	-579	409	-488	18	314	20
PAX6_01	PAX6	NNNNTTCACGCWTGANTKNNN	-46	-59	92	-57	84	-27	-43	-23	-30	-30
SP1_Q2_01	SP1	CCCCGCCCCN	20	17	183	22	349	434	616	21	300	61
TAXCREB_01	CREB	GGGGGTTGACGYANA	-87	-79	468	-101	388	-223	-259	-73	-243	-87
CKROX_Q2	CKROX	SCCCTCCCC	63	53	308	74	450	582	675	73	487	196
WT1_Q6	WT1	SMCNCCNSC	39	33	154	42	278	-444	-289	43	-575	200
PAX4_03	PAX4	NNNNNYCACCCB	90	54	-15	122	-13	9	9	37	7	343
MYOGNF1_01	NFIA	CRSCTGTTBNNTTTGGCACGSN GCCARCH	-33	-25	-67	-37	-180	144	92	-141	198	-182
PAX4_01	PAX4	NGNVGTCANGCGTGNNSNNYN	-72	-111	-149	-85	-144	274	227	-142	299	-159
SMAD3_Q6	SMAD3	TGTCTGTCT	-88	-87	-62	-105	-70	103	97	-333	114	435
POU3F2_01	POU3F2	ATGMATWWATTCAT	-339	-490	-90	-289	-53	78	87	186	78	336
HNF3B_01	HNF3B	KGNANTRTTTRYTTW	-312	571	-111	-472	-91	88	96	195	93	254
CART1_01	CART1	NNNTAATTNNCATTANCN	-613	-445	-374	-508	-331	379	400	448	358	588
KID3_01	KID3	CCACN	319	305	-265	358	-250	204	222	178	187	441
PAX4_02	PAX4	NAAWAATTANS	283	-538	-195	416	-125	145	183	133	162	128
PAX6_Q2	PAX6	CTGACCTGGAACTM	-421	-349	475	-493	322	-377	-431	-326	-353	-495
PLZF_02	PLZF	KTNNWTNGNNGNTAAAGYTTKA TYWGTTC	-307	-327	-203	-314	-199	236	228	550	211	-333
POU1F1_Q6	POU1F1	ATGAATAAWT	691	365	-467	-605	-411	463	470	558	467	-595
MINI19_B	-	NNNNGVCNCCAYNCMRSNGSM	-461	-452	-143	-433	-154	137	137	234	132	472

Table S13.

Summary of the genome versions that are used in this study.

Common Name	Scientific Name	Assembly	Accession	Source
dog (Boxer1)	Canis lupus familiaris	CanFam3.1	GCA_000002285.2	Ensembl (release 102)
dog (Boxer2)	Canis lupus familiaris	Dog10K_Boxer_Tasha	GCA_000002285.4	Ensembl (release 106)
dog (Basenji)	Canis lupus familiaris	Basenji_breed-1.1	GCA_004886185.1	Ensembl (release 102)
dog (Great Dane)	Canis lupus familiaris	UMICH_Zoey_3.1	GCA_005444595.1	Ensembl (release 102)
dog (Labrador Retriever)	Canis lupus familiaris	ROS_Cfam_1.0	GCA_014441545.1	Ensembl (release 106)
dog (German Shepherd)	Canis lupus familiaris	UU_Cfam_GSD_1.0	GCF_011100685.1	NCBI (Annotation Release 106)
dingo (dingo1)	Canis lupus dingo	ASM325472v1	GCA_003254725.1	Ensembl (release 102)
dingo (dingo2)	Canis lupus dingo	UNSW_AlpineDingo_1.0	GCF_012295265.1	NCBI (Annotation Release 101)
red fox	Vulpes vulpes	VulVul2.2	GCA_003160815.1	Ensembl (release 102)
dhole	Cuon alpinus	-	GWHAAAC00000000	iDog (http://bigd.big.ac.cn/idog)
gray wolf	Canis lupus	-	GWHAAAB00000000	iDog (http://bigd.big.ac.cn/idog)
human	Homo sapiens	GRCh38.p14	GCF_000001405.40	NCBI (Annotation Release 110)
chimpanzee	Pan troglodytes	Pan_tro_3.0	GCA_000001515.5	Ensembl (release 102)
Sumatran orangutan	Pongo abelii	PPYG2	-	Ensembl (release 102)
western lowland gorilla	Gorilla gorilla gorilla	gorGor4	GCA_000151905.3	Ensembl (release 102)

Table S14.

Important genes in the enriched gene sets relating to long-term memory, myelination, and cochlea development. These genes rank high in the fourth gene-eigenvector of dog/human, whereas rank lower in wolf/chimpanzee.

Category	Gene	Rank in dog	Rank in human	Biological processes
Long-term memory	*ABAT*	956	482	GABAergic synapse
	ADCY4	1945	735	cAMP biosynthetic process<br>cAMP signaling pathway<br>glutamatergic synapse<br>cholinergic synapse<br>GABAergic synapse
	ADRB2	-367	-1095	cAMP signaling pathway
	APOE	916	1009	long-term memory<br>negative regulation of long-term synaptic potentiation
	CFTR	156	679	cellular response to cAMP
	DMTN	1084	1971	cellular response to cAMP
	GATM	-1667	-544	learning or memory
	GNB3	1096	864	glutamatergic synapse<br>serotonergic synapse<br>GABAergic synapse<br>dopaminergic synapse
	GNG13	744	-1413	glutamatergic synapse<br>serotonergic synapse<br>neurotransmitter receptors and postsynaptic signal transmission
	GPD1	1906	1879	cellular response to cAMP
	PDE2A	1942	1721	cAMP-mediated signaling<br>cellular response to cAMP
	PLCB3	492	1618	long-term potentiation<br>long-term depression
	PLK2	-826	-875	memory<br>long-term synaptic potentiation<br>long-term synaptic depression
	PPP2CA	-390	-859	long-term depression
	SLC8A2	165	413	memory<br>long-term synaptic potentiation

Table S14 (continued).

Important genes in the enriched gene sets relating to long-term memory, myelination, and cochlea development. These genes rank high in the fourth gene-eigenvector of dog/human, whereas rank lower in wolf/chimpanzee.

Category	Gene	Rank in dog	Rank in human	Biological processes
Myelination	*ACSL1*	-1390	-1552	long-chain fatty acid metabolic process<br>fatty acid biosynthesis<br>fatty acid metabolism<br>fatty acid metabolism
	APOC1	712	1239	negative regulation of cholesterol transport<br>negative regulation of fatty acid biosynthetic process<br>cholesterol metabolism
	APOC3	644	561	cholesterol homeostasis<br>negative regulation of fatty acid biosynthetic process<br>cholesterol metabolism
	APOE	916	1009	negative regulation of cholesterol biosynthetic process<br>cholesterol homeostasis<br>fatty acid homeostasis<br>cholesterol metabolism
	ARV1	-310	-732	cholesterol biosynthetic process<br>cholesterol transport<br>regulation of intracellular cholesterol transport<br>cholesterol biosynthesis
	CFTR	156	679	cholesterol biosynthetic process<br>cholesterol transport
	EIF2B4	-838	-844	myelination<br>oligodendrocyte development
	MPZ	1776	869	myelination<br>myelin sheath
	MVK	-1982	-1043	cholesterol biosynthetic process<br>regulation of cholesterol biosynthesis by SREBP (SREBF)<br>cholesterol biosynthesis
	SLC27A2	-1226	-1243	long-chain fatty acid metabolic process
Cochlea development	*COL11A2*	1088	204	sensory perception of sound
	SCRIB	860	-613	auditory receptor cell stereocilium organization
